# Engineering Hierarchically Nano‐Structured Cu Foams: Dynamic Hydrogen Bubble Templated Binder‐Free Freestanding Electrodes for Energy Applications

**DOI:** 10.1002/smll.202509389

**Published:** 2025-12-26

**Authors:** Mina Attia, Chen Zhao, Miriam Lindner, Philipp Hawe, Christina Roth

**Affiliations:** ^1^ Department of Electrochemical Process Engineering Faculty of Engineering Science University of Bayreuth Universitätsstraße 30 95447 Bayreuth Germany

**Keywords:** binder‐free nano‐structures, CO_2_ reduction, cu electrodeposition, dynamic hydrogen bubble templating, free‐standing Cu foams, power‐to‐value

## Abstract

The intelligent design of hierarchical metallic structures with optimized performance for targeted applications, such as energy devices, sensors, and catalysis, remains a significant challenge. In this study, electrochemically generated hydrogen bubbles are employed as dynamic negative templates for copper electrodeposition. This so‐called dynamic hydrogen bubble templating approach (DHBT) yields highly porous hierarchical copper foams adorned with surface nano‐structures. A comprehensive investigation of DHBT synthesis parameters is provided, organized into four categories: (1) deposition current density and time; (2) current modes, namely direct, pulsed, reversed, and alternating regimes; (3) physical conditions, including stirring and temperature; and (4) bath composition. The results demonstrate that morphological descriptors, such as pore size and density, foam thickness, electrochemically active surface area (ECSA), and nanoscale surface features, can be systematically and reproducibly tuned by varying these DHBT parameters. As a proof of concept, a simple three‐step protocol for the fabrication of copper foam gas diffusion electrodes (GDEs) is presented. The resulting GDEs show promising CO_2_ reduction performance, achieving C_2+_ products Faradaic efficiencies of approximately 50% at ‐1.1 V versus reversible hydrogen electrode (RHE) and partial current densities of up to 104 mA cm^−2^ at ‐2.5 V versus RHE, with good operational stability tested for 12 h.

## Graphical abstract

The competition between H_2_ evolution (HER) and Cu electrodeposition during the dynamic hydrogen bubble templating (DHBT) crucially influences the synthesized foam morphology. Generally, when HER is dominating, numerous small H_2_ bubbles form, resulting in compact Cu foams. Conversely, when Cu electrodeposition outcompetes, fewer and larger H_2_ templates form, building up open Cu foam morphologies.

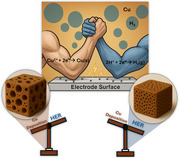

## 1 Introduction

Porous metallic structures have attracted much research interest in academia and industry due to their unique properties and versatile uses. Their high electric conductivity, large surface area, and light mass render them functional for many relevant applications, including thermal management, sensors, and energy applications.^[^ ^1^, ^2^ ^]^ Particularly, nano‐structured porous metal‐based materials are considered essential components in electrochemical systems such as fuel cells, metal‐air batteries, and electrolyzers. In these applications, the porous nano‐structures serve as catalytically active yet dimensionally stable electrodes, effectively lowering the energy barriers for the transformation of reactants and intermediates, thereby enhancing overall system performance.^[^ ^2^ ^]^ To illustrate this, a typical electrocatalytic process can be broken down into five key steps: (1) diffusion of reactants from the bulk electrolyte to the electrode surface, (2) adsorption of reactants onto the electrode, (3) charge transfer between the electrode and the reactants/intermediates, (4) desorption of the resulting products from the electrode surface, and (5) diffusion of these products into the bulk electrolyte.^[^ ^1^ ^]^ On the one hand, diffusion characteristics can be optimized through thoughtful design of the device and catalyst. For example, highly porous gas diffusion electrodes integrated into flow cell systems can achieve technically relevant current densities and conversion efficiencies, effectively overcoming the mass transport limitations typically encountered in traditional planar electrode configurations.^[^ ^2^, ^3^ ^]^ On the other hand, the adsorption, desorption, and charge transfer behaviors of reactants and intermediates are largely influenced by the nature and distribution of active sites on the electrocatalyst surface. Porous nano‐structures generally exhibit enhanced performance in these aspects due to their abundance of active sites and surface defects, which introduce confinement effects and modulate adsorption and charge transfer characteristics.^[^ ^1^ ^]^ According to the International Union of Pure and Applied Chemistry (IUPAC), porous materials are classified based on pore size into three categories: microporous (with porous structures smaller than 2 nm), mesoporous (with pores between 2 and 50 nm), and macroporous (with pores larger than 50 nm).^[^ ^1^, ^4^ ^]^ Additionally, a material is considered “hierarchical” when it incorporates at least two of these pore size domains, and a porous structure is commonly termed a foam when a substantial volume fraction of the solid is occupied by gas‐filled pores or dispersed bubbles.^[^ ^4^ ^]^

**Figure 1:**
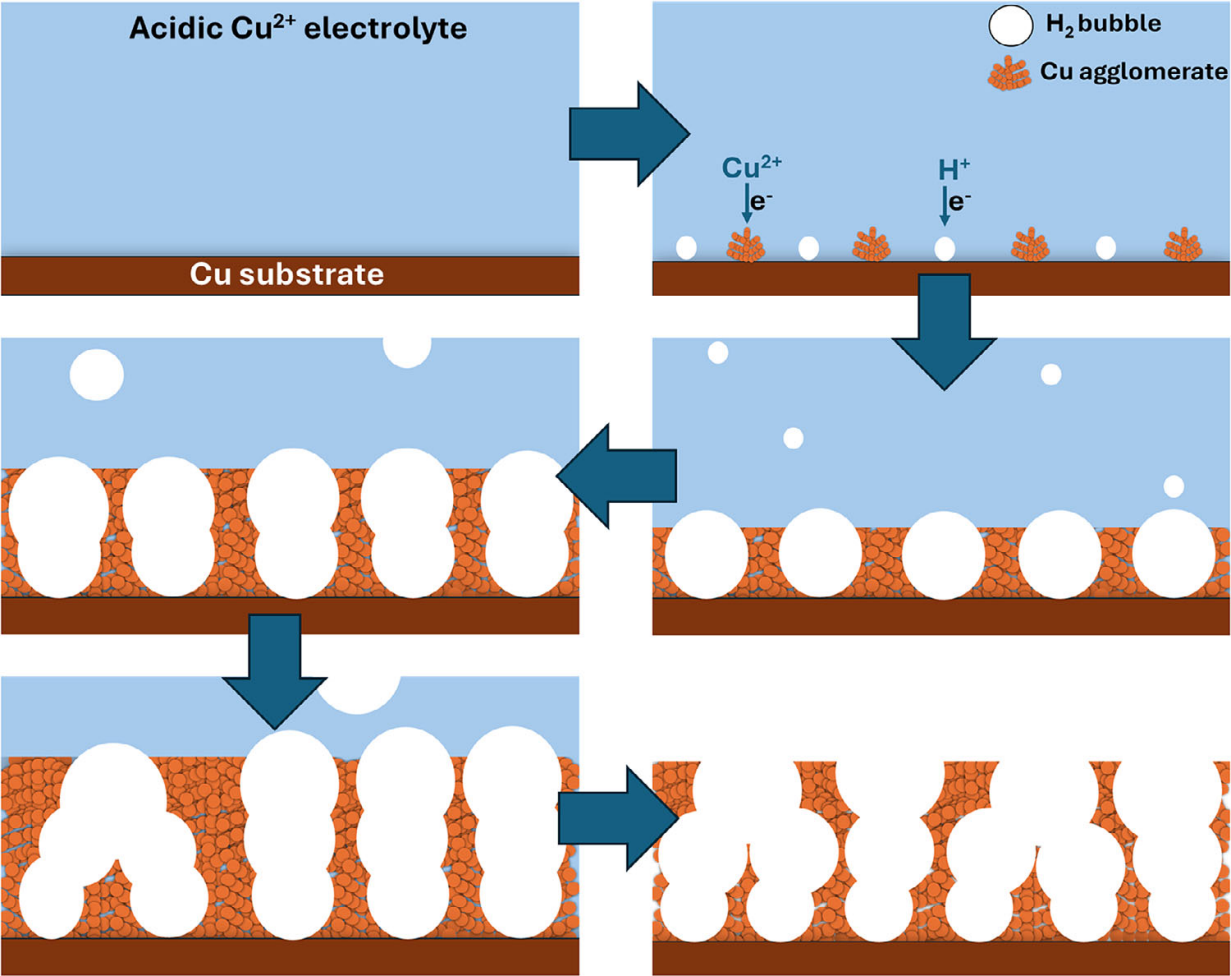
A possible schematic representation of the different stages of a Cu foam synthesis via the DHBT method.

Numerous methods have been reported in the literature for fabricating metal foams, including selective dissolution, templating, combustion, and the sol‐gel technique. These approaches are usually multi‐step, complicated, and exclusive methods that suffer from limitations in affording suitable precursors, applying relatively higher temperatures and pressures, working under inert environments, and/or the necessity of additional purification steps.^[^ ^5^, ^6^, ^7^, ^8^, ^9^, ^10^, ^11^, ^12^ ^]^ In contrast, the dynamic hydrogen bubble templating method (DHBT) represents a more recent advancement. It offers a single‐step, inclusive, versatile, and scalable strategy for the controlled synthesis of hierarchical metal foams, without the need for external organic or inorganic templates or subsequent purification.^[^ ^5^, ^13^ ^]^ Electrochemical experiments are preferentially conducted within the solvent's electrochemical stability window to prevent the decomposition of solvent molecules. Therefore, during the electrodeposition of both noble and base metals, such as Cu, Ag, Au, Pt, Zn, Co, Fe, and Ni from their aqueous salt solutions, the simultaneous occurrence of the parasitic H_2_ evolution reaction (HER) poses a significant challenge. This parasitic reaction can lead to Faradaic efficiency losses and compromise the integrity of the deposited metal layer.^[^ ^5^, ^13^ ^]^ Interestingly, the DHBT method turns this challenge into an advantage. By applying high cathodic overpotentials or currents, HER is deliberately induced at elevated rates, generating hydrogen bubbles that act as dynamic templates for metal deposition with macro‐, meso‐, and micropore dimensions. Additionally, the generated H_2_ bubbles of different sizes, whose formation and behavior are governed by the DHBT parameters, create local stirring and alter the hydrodynamic conditions at the electrode surface. This results in the formation of self‐supporting nano‐structures within the metal foam walls with diverse morphologies, eliminating the need for binders or additional fixation steps for the use of metallic nano‐particles.^[^ ^5^, ^13^, ^14^ ^]^ **Figure** **1** provides a possible schematic representation of the DHBT electrodeposition of a Cu foam sample. It illustrates the sequential stages of the DHBT process, starting with the nucleation of H_2_ bubbles alongside Cu electrodeposition. As the process continues, the bubbles grow and coalesce, acting as dynamic negative templates for Cu deposition, ultimately leading to the formation of a porous Cu foam structure.

Among metal foams, Cu foams are particularly noteworthy due to their involvement in a wide range of applications, including nitrate removal, CO_2_ reduction reaction (CO_2_RR), nitrate‐to‐ammonia conversion, volatile organic compounds removal, lithium‐ion and zinc‐based alkaline batteries, oxygen and hydrogen evolution reactions (OER and HER), enhancement of thermal conductivity in carbon‐based composites, supercapacitors, and sensors, etc.^[^ ^15^, ^16^, ^17^, ^18^, ^19^, ^20^, ^21^, ^22^, ^23^, ^24^, ^25^, ^26^, ^27^, ^28^, ^29^ ^]^ The aim of this work is to provide a comprehensive study into the engineering of Cu foams via the DHBT method, with a particular emphasis on elucidating how various DHBT parameters influence hydrogen bubble evolution and growth dynamics, as well as Cu electrodeposition behavior, and consequently, the resulting Cu foam morphology. The DHBT parameters investigated in this work are categorized into four principal groups:

(1) Current‐time series: examining the most fundamental DHBT parameters, namely, deposition time and applied continuous cathodic direct current density.
(2) Current mode series: investigating the effect of applying different current regimes, direct current (DC), pulsating current (PC), reversing current (RC), and alternating current (AC) during DHBT.
(3) Physical effect series: assessing the impact of physical variables such as stirring, temperature, electrode orientation, and post‐synthesis annealing.
(4) Chemical effect series: evaluating the impact of various chemical parameters, including Cu^2+^ concentration, pH, and the presence of additives. Two categories of additives were investigated: (a) additives influencing hydrogen bubble evolution, such as surfactants, and (b) additives affecting Cu electrodeposition behavior, such as complexing agents.

Our results demonstrate that the morphology of the Cu foams can be effectively tuned by adjusting the DHBT parameters. Therefore, the intelligent design of Cu foams for a particular application, e.g., electrocatalytic CO_2_RR, along with tailoring the process performance and selectivity, can be affordable, which is an outlook for our current research. Herein, in order to demonstrate the applicability of DHBT to energy–storage and conversion devices, a simple three–step protocol for the synthesis of Cu foam GDEs is presented, and yields electrodes with promising CO_2_RR activity.

## 2 Results and Discussion

### 2.1 Current‐Time Series

The impact of the two most fundamental DHBT parameters, deposition current density and time, was examined by fabricating Cu foams under varying deposition current densities and times, followed by morphological analysis of the resulting samples. Two DHBT cathodic current densities, 1 A cm^−2^ and 2 A cm^−2^, were employed. For the 1 A cm^−2^ condition, electrodeposition was conducted for 10, 20, and 30 s, whereas for the 2 A cm^−2^ condition, durations of 5, 10, and 15 s were used. Synthesizing samples at a constant current density with varying durations allowed for isolating the effect of DHBT time and visualizing the different stages of DHBT. Each sample produced at 1 A cm^−2^ was matched with a corresponding sample at 2 A cm^−2^ that received the same total charge density, ensuring that the influence of charge was excluded when assessing the effect of DHBT current density and thereby providing a fair comparison.

**Figure 2:**
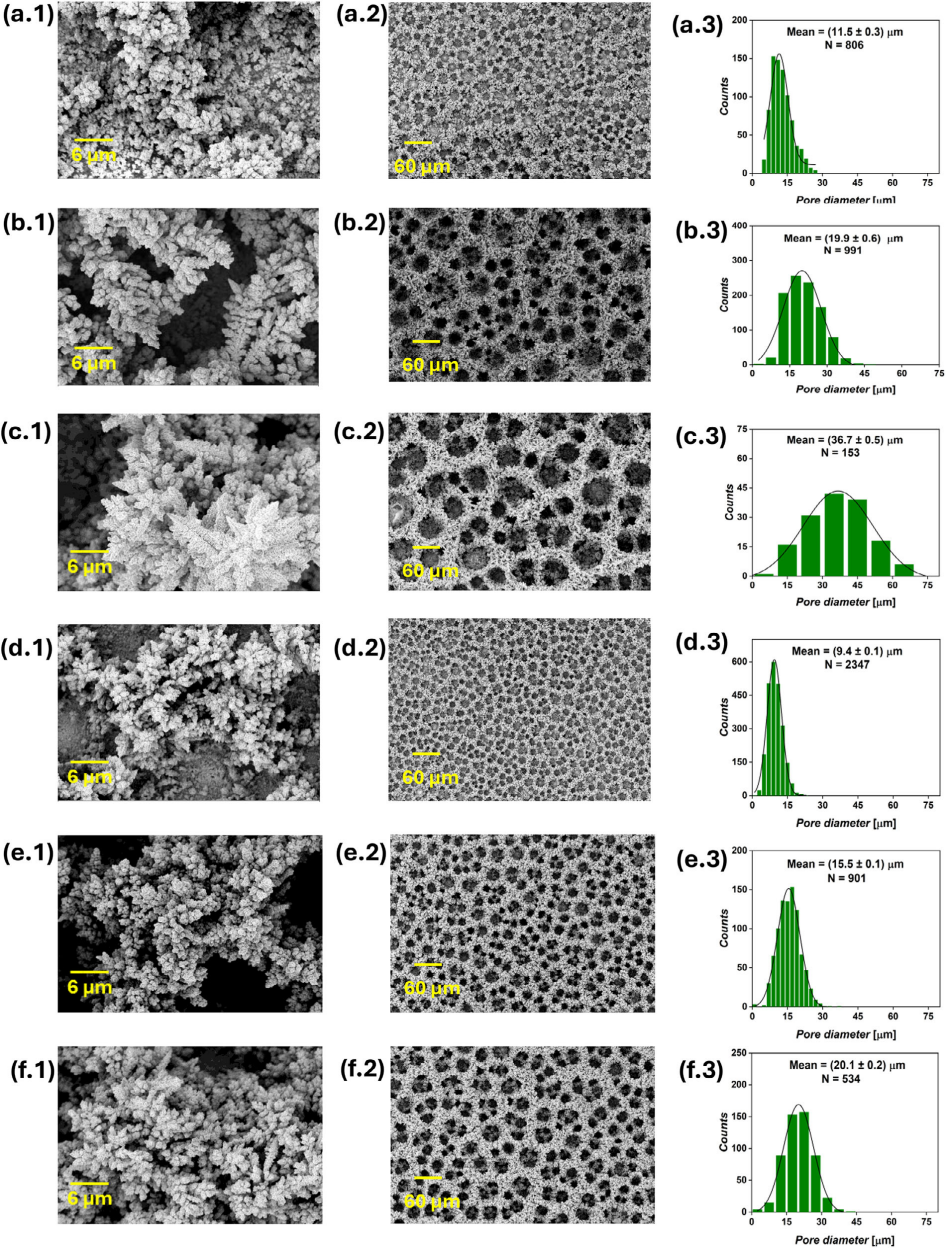
SEM images of the current‐time DHBT series, showing the surface nano‐dendrites at 3000x magnification (first column), foam structure at 200x magnification (second column), and pore diameter distribution extracted from the 200x images (third column) of the (1 A cm^−2^ 10 s) a), (1 A cm^−2^ 20s) b), (1Acm^−2^ 30s) c), (2Acm^−2^ 5s) d), (2 A cm^−2^ 10 s) e), and (2 A cm^−2^ 15 s) f), where *N* is the number of pores from which the pore diameter distribution was constructed.

Foam structures were observed in all the current‐time series samples, as shown in **Figure** **2**, confirming that the applied cathodic bias was sufficient to simultaneously sustain both hydrogen evolution and Cu electrodeposition at significant rates. As electrolysis proceeds, the surface pore diameter progressively expands, as observed in top‐view SEM images taken at 200x magnification. This indicates that extending the DHBT duration enhances the coalescence of H_2_ bubbles, leading to a hierarchical structure with smaller cavities near the substrate and larger ones toward the solution side. Increasing the DHBT current density led to a higher pore density and smaller pore diameter, as evident by comparing the SEM images of Cu foam samples with equal charges, such as (1Acm^−2^ 10 s) versus (2Acm^−2^ 5 s), (1Acm^−2^ 20 s) versus (2Acm^−2^ 10 s), etc. This suggests that higher DHBT currents promote the formation of more HER nucleation sites on the substrate surface, in contrast to lower currents, where surface irregularities particularly drive H_2_ evolution. All six Cu foam samples exhibited surface nano‐dendrites, though their sizes vary, as seen in high‐magnification SEM images. At a constant current density, increasing the DHBT time results in larger nano‐dendrites on the top surface. Conversely, higher DHBT current densities led to smaller nano‐dendrite sizes. Pore diameter distribution histograms were generated from the SEM images to determine the average pore diameter. Additionally, SEM images taken at the cross‐section of the Cu foams were used to determine the foam thickness (Figure S1, Supporting Information). Details on how pore diameters and foam thicknesses were measured from SEM images, together with the statistical analyses, are given in Section 5. A positive correlation between pore diameter and nano‐dendrite size can be made, and can be explained by the intensified stirring effect at the electrode surface, which occurs when larger H_2_ bubbles are generated. These bubbles enhance the mass transport of Cu^2+^ ions from the bulk electrolyte to the electrode surface, thereby reducing the dimensions of the diffusion layer and increasing the limiting current density. The rise in limiting current density lowers the overpotential for Cu electrodeposition,^[^ ^30^ ^]^ leading to the formation of larger nano‐dendrites. Conversely, smaller bubbles result in reduced electrolyte stirring, lower limiting current density, and smaller nano‐dendrites. Experimental evidence supporting this hypothesis is provided in Section 2.3, where external mass convection was applied during DHBT. In this work, the term “compact structure” refers to a Cu foam morphology distinguished by high pore density, small pore diameter, reduced foam thickness, and finely branched surface nano‐structures. This is contrasted with the “open structure,” which features lower pore density, larger pore diameter, increased thickness, and larger surface nano‐structures. Among the time‐current series samples, the sample (2 A cm^−2^ 5 s) exhibited the most compact structure, characterized by the smallest pore diameter (9.4 ± 0.1 µm), highest pore density (≈3541 pore mm^−2^), and lowest foam thickness (23.0 ± 1.5 µm). In contrast, the sample (1Acm^−2^ 30s) exhibited the most open structure, characterized by the lowest pore density (≈346 pore mm^−2^), largest mean pore diameter (36.7 ± 0.5 µm), and greatest foam thickness (62.0 ± 4.7 µm).

Equation (1) was used to calculate the DHBT Faradaic efficiency towards Cu electrodeposition (*FE*_*Deposition*_), where *F* is Faraday's constant, *n* is the number of electrons transferred to electrodeposit one Cu atom, *m*_*Deposited*_ is the mass of Cu electrodeposited, *Q* is the DHBT total charge, and *M*_*Cu*_ is Cu atomic mass

(1) $$FE_{\mathrm{Depo}\mathrm{siti}\mathrm{on}}=\frac{m_{\mathrm{Depo}\mathrm{sited}}\cdot n\cdot F}{Q\cdot M_{\mathrm{Cu}}}\cdot 100\%$$

**Figure 3:**
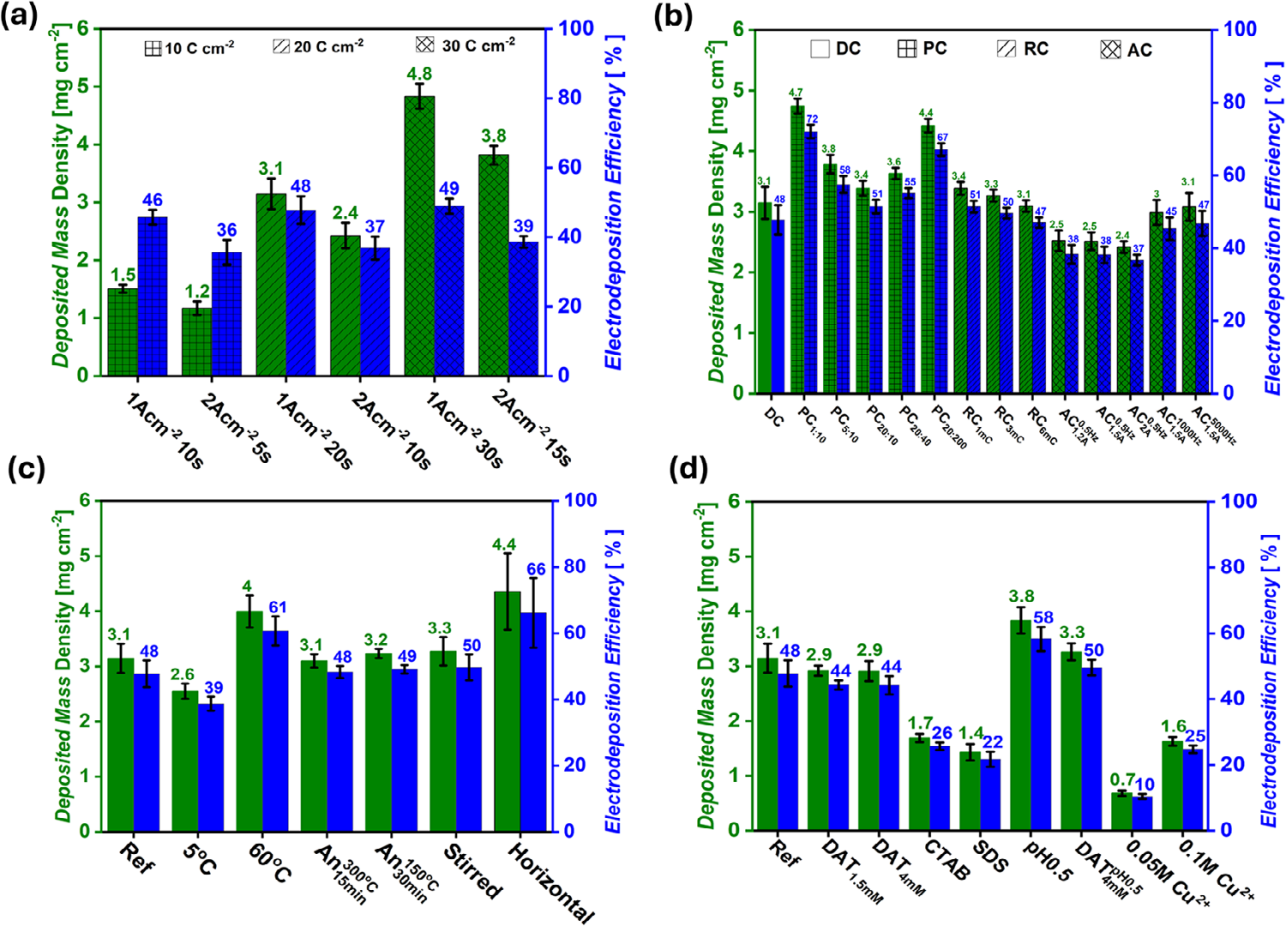
Electrodeposited mass densities and electrodeposition Faradaic efficiencies of the current‐time a), current mode b), physical DHBT effects c), and chemical DHBT effect series d).

**Figure** **3**a shows the gravimetric and electrodeposition Faradaic efficiency analysis of the current‐time series. At constant DHBT current densities (1 A cm^−2^ or 2 A cm^−2^), increasing the DHBT time led to a gradual increase in electrodeposited Cu mass density. For instance, at 1 A cm^−2^, the Cu mass densities for samples (1 A cm^−2^ 10 s), (1 A cm^−2^ 20 s), and (1 A cm^−2^ 30 s) were 1.5 ± 0.1, 3.1 ± 0.3, and 4.8 ± 0.2 mg cm^−2^, respectively, agreeing with the increasing DHBT charge over time. These correspond to Faradaic efficiencies of 46 ± 2, 48 ± 4, and 49 ± 2%, indicating a strong influence of DHBT duration on deposited Cu mass, but only a minor effect on Faradaic efficiency. From an engineering perspective, high Faradaic efficiencies for the metal deposition process are favored. In contrast, increasing the DHBT current density from 1 A cm^−2^ to 2 A cm^−2^ reduced the Cu mass densities to 1.2 ± 0.1, 2.4 ± 0.2, and 3.8 ± 0.2 mg cm^−2^ with corresponding Faradaic efficiencies of 36 ± 4%, 37 ± 3%, and 39 ± 2% for the samples (2 A cm^−2^ 5 s), (2 A cm^−2^ 10s), and (2Acm^−2^ 15s), respectively. The gravimetric results support the SEM findings, suggesting that higher current densities promote H_2_ evolution, thereby lowering the Faradaic efficiency for Cu deposition.

The electrochemically active surface area (ECSA) was determined by extracting the double‐layer capacitance (*C*_*DL*_) of the Cu foams using cyclic voltammetry (CV) and electrochemical impedance spectroscopy (EIS) measurements conducted at non‐Faradaic potentials. The ECSA was then calculated according to Equation 2, where *C*_*ref*_ denotes the double‐layer capacitance of a flat Cu surface of 1 cm^2^ area, which was experimentally determined. All ECSA values were normalized to the Cu foam geometric area and reported in the unit of ${\mathrm{cm}}_{\mathrm{ECSA}}^{2}$.${\mathrm{cm}}_{\mathrm{geo}}^{-2}$. The details of the ECSA measurement experiments and the determination of the reference value (*C*_*ref*_) are described in Section 5. Representative CV and EIS data used for ECSA estimation are shown in Figures S2 and S3 (Supporting Information)

(2) $$ECSA=\frac{C_{DL}}{C_{ref}}$$

**Figure 4:**
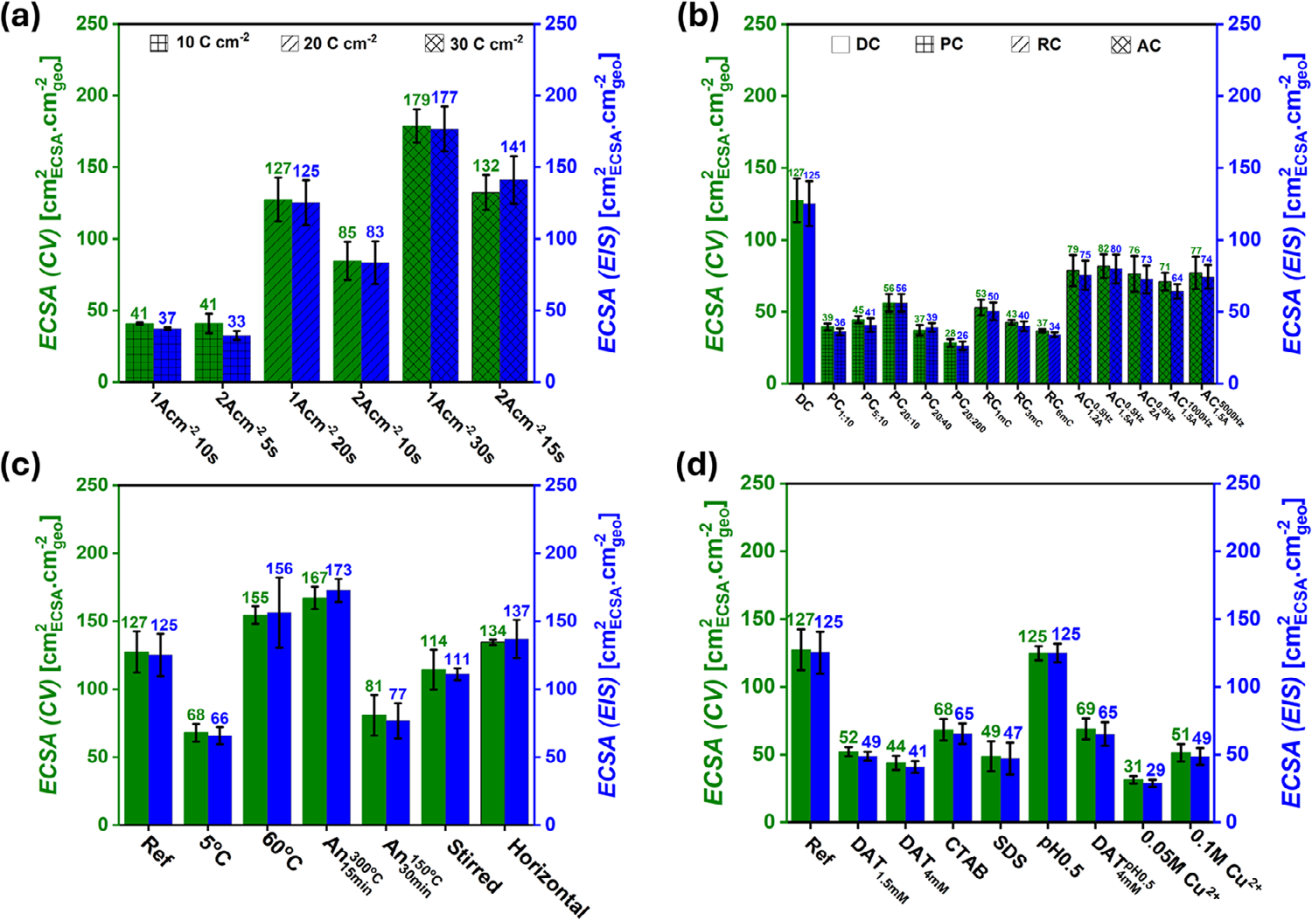
Electrochemically active surface area determined by cyclic voltammetry (green axis) and electrochemical impedance spectroscopy (blue axis) of Cu foam samples of 1 cm^2^ geometric area for the series: current–time a), current modes b), physical DHBT effects c), and chemical DHBT effects (d).

Across all samples, the ECSA values obtained via CV and EIS were found to be in close agreement, as can be seen in **Figure** **4**, and consequently, only CV‐derived ECSA values will be mentioned within the text. Consistent with SEM and gravimetric analyses, increasing the DHBT current density while maintaining a constant electrodeposition charge resulted in a decrease in ECSA. For example, at a DHBT charge density of 20 C cm^−2^, increasing the applied current density from 1 to 2 A cm^−2^ resulted in a decline in the ECSA from 127 ± 15 to 85 ± 13 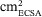.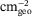. Conversely, extending the DHBT duration led to an increase in ECSA. For instance, at 1 A cm^−2^, the ECSA increased from 41 ± 1 to 179 ± 12 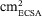.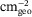 when the DHBT time was increased from 10 to 30 s of deposition, as shown in Figure 4a.

The findings of the current‐time series align with previous studies, indicating that extending the DHBT duration produces more open foams.^[^ ^5^ ^]^ In contrast, increasing the DHBT current density promoted the formation of more active sites for bubble formation, thereby enhancing pore density. The ability to achieve graded structures simply by adjusting the DHBT current density or duration makes DHBT an ideal and versatile method for preparing electrocatalyst materials with precisely controlled pore sizes. Additionally, open‐structured Cu foams are highly advantageous as current collectors in batteries and supercapacitors, where their excellent conductivity and relatively large surface area contribute to enhanced electrochemical performance. In contrast, compact metallic structures have demonstrated superior electrocatalytic activity in certain applications, such as the reduction of CO_2_ to C_1_ products on Ag foams,^[^ ^31^ ^]^ suggesting that compact Cu foams may similarly outperform open porous foams in the conversion of CO_2_ to C_2 +_ products.

### 2.2 Current Mode Series

**Figure 5:**
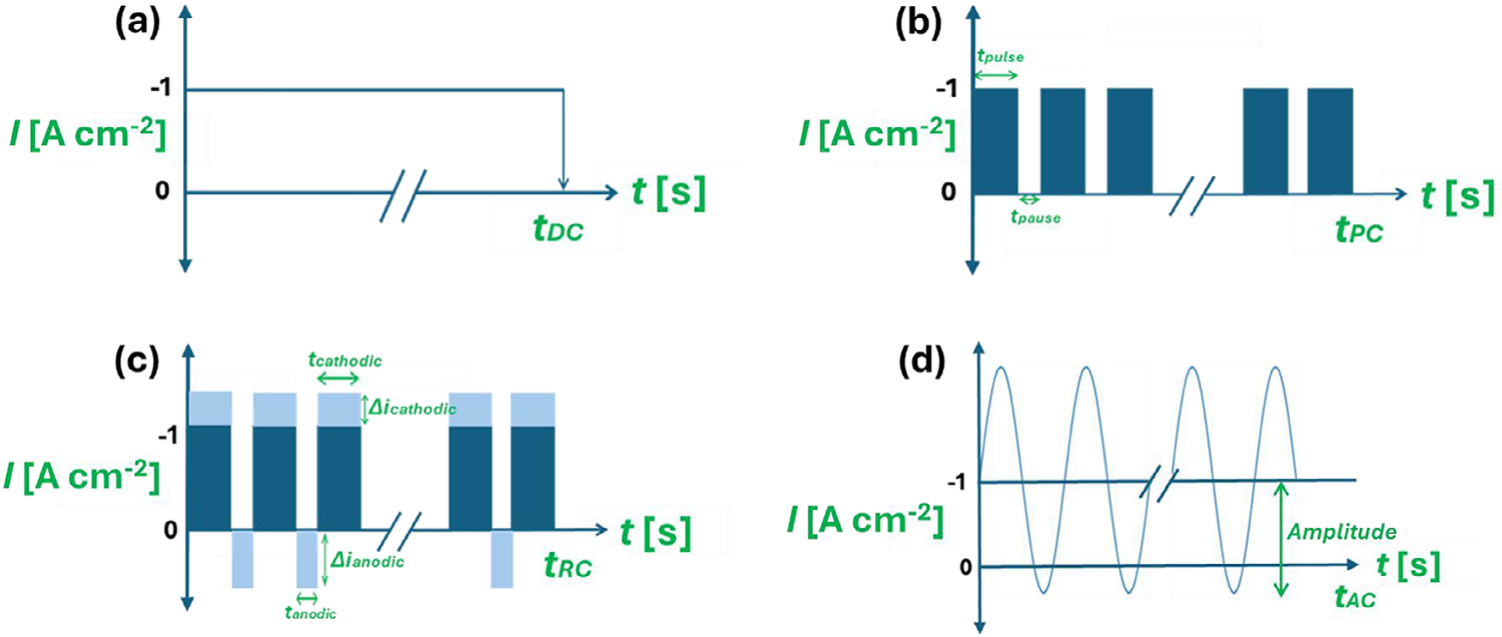
Schematic representation of the DC a), PC b), RC c), and AC d) DHBT modes, where *t*_*DC*_, *t*_*PC*_, *t*_*RC*_, and *t*_*AC*_ are the total times of the DC, PC, RC, and AC electrodeposition, *t*_*pulse*_, *t*_*pause*_, *t*_*cathodic*_, and *t*_*anodic*_ are the PC pulse, PC pause, RC cathodic pulse, and RC anodic pulse times, Δ*i*_cathodic_ and Δ*i*_anodic_ are the additional current densities in the cathodic and anodic directions in case of RC mode with respect to the PC mode, respectively.

Four distinct current modes for Cu electrodeposition have been reported in the literature: direct,^[^ ^12^ ^]^ pulsating,^[^ ^32^ ^]^ reversing,^[^ ^33^, ^34^ ^]^ and alternating current,^[^ ^35^ ^]^ and were employed in this study for synthesizing Cu foams via the DHBT method. As illustrated in **Figure** **5**, a continuous DC signal is applied for a defined duration (*t*_*DC*_) in the DC mode.

Conversely, the PC‐DHBT involves applying short cathodic pulses of duration *t*_*pulse*_, separated by pause intervals *t*_*pause*_, both in the ms time scale, and repeated over a set number of cycles. The RC mode alternates between anodic and cathodic pulses, with respective durations *t*_*anodic*_ and *t*_*cathodic*_, and the cathodic charge density is maintained significantly higher to ensure net deposition. The RC total charge can be essentially tuned by adjusting the anodic and cathodic current densities, denoted as *i*_*anodic*_ and *i*_*cathodic*_. The anodic charge density per single anodic pulse is denoted as Δ*C*, which is the product of *t*_*anodic*_ and *i*_*anodic*_. In our work, to make a comparison between the PC and RC modes, the same pulse separation (i.e., *t*_*pulse*_ = *t*_*cathodic*_ and *t*_*pause*_ = *t*_*anodic*_), and cycle count were applied for both modes. Additionally, an extra cathodic current density (Δ*i*_*cathodic*_) with respect to the PC pulse current density (*i*_*pulse*_) has to be applied to cancel the anodic charge density (Δ*C*) out and keep the total charge density of both RC and PC foams the same, i.e., *i*_*cathodic*_ = *i*_*pulse*_ + Δ*i*_*cathodic*_. In the AC mode, an AC wave is superimposed on a DC offset. The key parameters in this mode are the frequency and amplitude of the AC signal. Previous research has demonstrated that, to achieve markedly different AC structures compared to those formed under a pure DC signal, the amplitude of the AC component must surpass the DC offset.^[^ ^35^ ^]^ The AC signal half‐waves cancel each other out, and the total applied charge can be simply calculated as the product of the electrodeposition DC offset and time.

#### 2.2.1 Pulsating and Reversing Current Modes

Five PC samples were synthesized using the same pulse current density of 1 A cm^−2^ and a total cathodic charge density of 20 C cm^−2^, but with varying pulse and pause durations. In a constant‐pause set (pause time fixed at 10 ms), three different pulse durations,1, 5, and 20 ms, were individually tested. In a constant‐pulse set (pulse time fixed at 20 ms), three pause durations, 10, 40, and 200 ms, were individually examined. Notably, the sample synthesized with 20 ms pulse and 10 ms pause appears in both sets. These experimental conditions enabled the isolation of pulse and pause duration effects on the resulting morphology, yielding three distinct pulse‐to‐pause ratios: 0.1, 0.5, and 2. The five synthesized PC samples were labeled according to their pulse and pause durations as follows: PC_1:10_, PC_5:10_, PC_20:10_, PC_20:40_, and PC_20:200_, and were received corresponding pulse‐pause cycles of 20 000, 4000, 1000, 1000, and 1000, and average current densities ($I_{avg}^{PC}$) of 0.09, 0.33, 0.67, 0.33, and 0.09 A cm^−2^, as calculated from Equation (3)

(3) $$I_{\mathrm{avg}}^{\mathrm{PC}}=\frac{i_{\mathrm{pulse}}}{1+\frac{t_{\mathrm{pause}}}{t_{\mathrm{pulse}}}}$$

**Figure 6:**
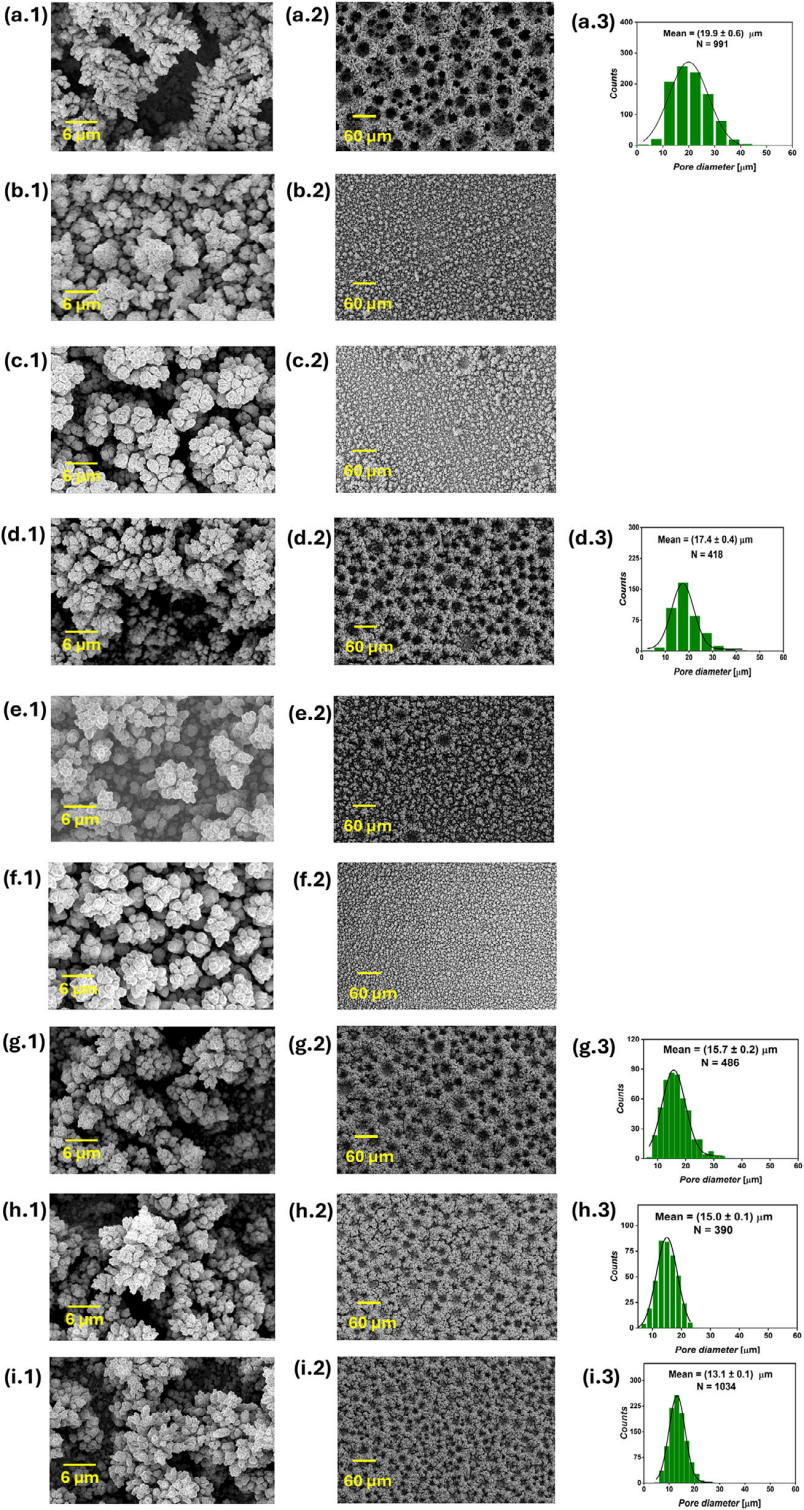
SEM images of PC and RC samples, showing the surface nano‐structures at 3000x magnification (first column), foam structure at 200x magnification (second column), and pore diameter distribution extracted from the 200x images (third column) of the DC reference a), PC_1:10_ b), PC_5:10_ c), PC_20:10_ d), PC_20:40_ e), PC_20:200_ f), RC_1mC_ g), RC_3mC_ h), and RC_6mC_ i), where *N* is the number of pores from which the pore diameter distribution was obtained. For the samples without a displayed pore diameter distribution, there were insufficient pore counts to generate a reliable distribution.

SEM analysis of the five PC samples is presented in **Figure** **6** and Figure S4 (Supporting Information). Among them, only the PC_20:10_ sample, corresponding to a pulse‐to‐pause ratio of 2, exhibited a well‐defined, honeycomb‐like interconnected structure. This sample showed a smaller average pore diameter (17.4 ± 0.4 µm) compared to the DC reference sample (19.9 ± 0.6 µm). At a pulse‐to‐pause ratio of 0.5 (PC_5:10_ and PC_20:40_), isolated foam islands with dish‐like craters were observed, which lacked uniform inter‐connectivity. The samples synthesized at a pulse‐to‐pause ratio of 0.1 (PC_1:10_ and PC_20:200_) showed almost no honeycomb‐like structure formation at all, producing instead agglomerations of Cu deposits. Foam thickness followed a volcano‐like trend across the PC samples, PC_1:10_, PC_5:10_, PC_20:10_, PC_20:40_, and PC_20:200_, with respective thicknesses of 19.7 ± 3.7, 23.8 ± 5.3, 46.2 ± 5.2, 25.7 ± 3.5, and 19.0 ± 1.6 µm. All PC samples were significantly thinner than the DC reference, which measured 50.6 ± 5.3 µm. Distinct differences in surface nano‐structures were evident at higher magnification: the DC sample featured nano‐dendrites, while PC samples exhibited cauliflower‐like agglomerates. The electrodeposited Cu mass density and the electrodeposition Faradaic efficiency exhibited similar trends across the PC samples. The Cu mass densities for PC_1:10_, PC_5:10_, PC_20:10_, PC_20:40_, and PC_20:200_ were 4.7 ± 0.1, 3.8 ± 0.2, 3.4 ± 0.1, 3.6 ± 0.1, and 4.4 ± 0.1 mg cm^−2^, corresponding to Faradaic efficiencies of 72 ± 2, 58 ± 2, 51 ± 2, 55 ± 1, and 67 ± 2%, respectively, as presented in Figure 3b. These values are notably higher than those of the DC reference sample, which showed a deposited mass density of 3.1 ± 0.3 mg cm^−2^ and electrodeposition Faradaic efficiency of 48 ± 4%. The PC results suggest that introducing current pauses during DHBT inhibits hydrogen bubble coalescence and results in higher mass density of the sample. The hindered H_2_ bubble growth is evidenced by the increased Faradaic efficiency for Cu deposition, implying reduced efficiency for H_2_ evolution, smaller pore diameter in PC_20:10_, absence of interconnected foam structures in the other PC samples, increased electrodeposited Cu mass density, and reduced thicknesses of the PC samples. Clearly, this hindrance of H_2_ bubble coalescence became more pronounced with decreasing the pulse‐to‐pause ratio. The high mass density, combined with enhanced meso‐ and microporosity and distinct surface nano‐structures, may lead to significantly different performance and operational stability of the Cu foams in various applications, including electrocatalysis, sensing, and fuel cells.

For all RC samples, the cathodic and anodic pulse durations were fixed at 20 and 10 ms, respectively, with a constant total cathodic charge density of 20 C cm^−2^. The RC pulse separation mirrored that of the PC_20:10_ sample, allowing for direct comparison with both the DC and PC modes. Three RC Cu foams were synthesized by varying the anodic charge density per pulse (Δ*C*), with values of 1, 3, and 6 mC cm^−2^. These were achieved by applying anodic current densities of 0.1, 0.3, and 0.6 A cm^−2^, respectively. To maintain a net cathodic charge density of 20 C cm^−2^, the cathodic pulse current densities were adjusted to 1.05, 1.15, and 1.3 A cm^−2^, correspondingly, effectively compensating for the anodic charge input. The average current density during DHBT for the three RC foams ($I_{avg}^{RC}$) and the reference PC_20:10_ sample was 0.67 A cm^−2^, as calculated from Equation (4). Figure 6 presents the top‐view SEM images of the RC Cu foams, with the cross‐sectional SEM images provided in Figure S4 (Supporting Information). The RC mode significantly influenced both pore diameter and foam thickness

(4) $$I_{\mathrm{avg}}^{\mathrm{RC}}=\frac{i_{\mathrm{cath}\mathrm{odic}}\;t_{\mathrm{cath}\mathrm{odic}}-i_{\mathrm{anod}\mathrm{ic}}\;t_{\mathrm{anod}\mathrm{ic}}}{t_{\mathrm{cath}\mathrm{odic}}+t_{\mathrm{anod}\mathrm{ic}}}$$

Compared to the PC_20:10_ sample, which displayed a mean pore diameter of 17.4 ± 0.4 µm, the RC foams exhibited a progressive reduction in pore diameter to 15.7 ± 0.2, 15.0 ± 0.1, and 13.1 ± 0.1 µm for Δ*C* values of 1, 3, and 6 mC cm^−2^, respectively. Similarly, foam thickness decreased from 46.2 ± 5.2 µm in PC_20:10_ to 39.1 ± 2.1, 29.4 ± 9.1, and 24.8 ± 5.3 µm in the RC_1mC_, RC_3mC_, and RC_6mC_ samples, respectively. The RC_6mC_ exhibited a higher pore density (≈936 pore mm^−2^) compared to the PC_20:10_ (≈631 pore mm^−2^). At the nanoscale, the RC foams retained the nano cauliflower‐like morphology observed in PC_20:10_, which remained distinctly different from the dendritic features seen in the DC sample. Moreover, a slight decrease in both the electrodeposited Cu mass density and the electrodeposition Faradaic efficiency was observed with increasing Δ*C* values, declining from 3.4 ± 0.1 mg cm^−2^ and 51 ± 2% in the PC_20:10_ sample to 3.1 ± 0.1 mg cm^−2^ and 47 ± 1% in the RC_6mC_ sample. These RC results indicate enhanced suppression of H_2_ evolution during DHBT when the working electrode charge is periodically reversed. Under identical pulse separation conditions, the RC mode led to increased pore density, reduced pore diameter, and decreased foam thickness, collectively contributing to a higher compactness of the electrodeposited Cu structures. The distinct surface nano‐structures observed in PC and RC samples, compared to DC foams, can be attributed to the different hydrodynamic conditions near the electrode surface during DHBT. While uninterrupted DHBT leads to the formation of surface nano‐dendrites of varying sizes, depending on the intensity of bubble‐induced stirring, as observed in the current‐time series, sudden and consecutive interruptions of the cathodic signal (via pauses or anodic pulses) have been reported to allow the diffusion layer to partially relax toward bulk conditions. This results in a thinner diffusion layer, thereby increasing the limiting current density and decreasing the overpotential,^[^ ^36^ ^]^ resulting in a fast growth of Cu agglomerates. However, due to the periodic and abrupt interruption of electrodeposition, the nano‐structures are not able to grow axially into dendrites. Instead, they tend to curl up, forming agglomerates of cauliflower‐like surface nano‐structures. Since unstable Cu particles are more likely to dissolve during anodic pulses, RC samples may offer improved operational stability compared to foams produced under PC and DC conditions.

The ECSA results of the PC and RC Cu foams are presented in Figure 4b. The PC and RC samples clearly exhibited significantly lower ECSA values compared to the DC reference sample, which showed a higher ECSA of 127 ± 15 ${\mathrm{cm}}_{\mathrm{ECSA}}^{2}$.${\mathrm{cm}}_{\mathrm{geo}}^{-2}$. A volcano‐like trend is again evident in the ECSA values of the PC samples. As the pulse‐to‐pause ratio increased from 0.1 (for the PC_1:10_ sample) to 2 (for the PC_20:10_ sample), the ECSA rose from 39 ± 2 to 56 ± 6 ${\mathrm{cm}}_{\mathrm{ECSA}}^{2}$.${\mathrm{cm}}_{\mathrm{geo}}^{-2}$. Subsequently, decreasing the pulse‐to‐pause ratio by extending the pause time to 200 ms, while maintaining a constant pulse duration of 10 ms, led to a reduction in ECSA to 28 ± 3 ${\mathrm{cm}}_{\mathrm{ECSA}}^{2}$.${\mathrm{cm}}_{\mathrm{geo}}^{-2}$ for the PC_20:200_ sample. The ECSA reults of the PC_20:10_ and RC samples, which shared identical pulse separation and pulse pair counts, revealed that applying the RC mode resulted in a pronounced decrease in ECSA. Increasing Δ*C* from 0 mC cm^−2^ (for the PC_20:10_ sample) to 6 mC cm^−2^ (for the RC_6mC_ counterpart) was accompanied by a gradual decline in ECSA from 56 ± 6 to 37 ± 1 ${\mathrm{cm}}_{\mathrm{ECSA}}^{2}$.${\mathrm{cm}}_{\mathrm{geo}}^{-2}$. The ECSA results are again consistent with the morphological features observed in the SEM images, particularly regarding sample openness and compactness. For instance, the DC sample, being more open than its PC and RC counterparts, exhibited the highest ECSA values. Increasing the pulse‐to‐pause ratio clearly enhanced the openness of the resulting Cu structures, leading to higher ECSA values compared to those obtained at low pulse‐to‐pause ratios. Conversely, the increased compactness associated with rising Δ*C* values resulted in a decline in the ECSA.

#### 2.2.2 Alternating Current Modes

**Figure 7:**
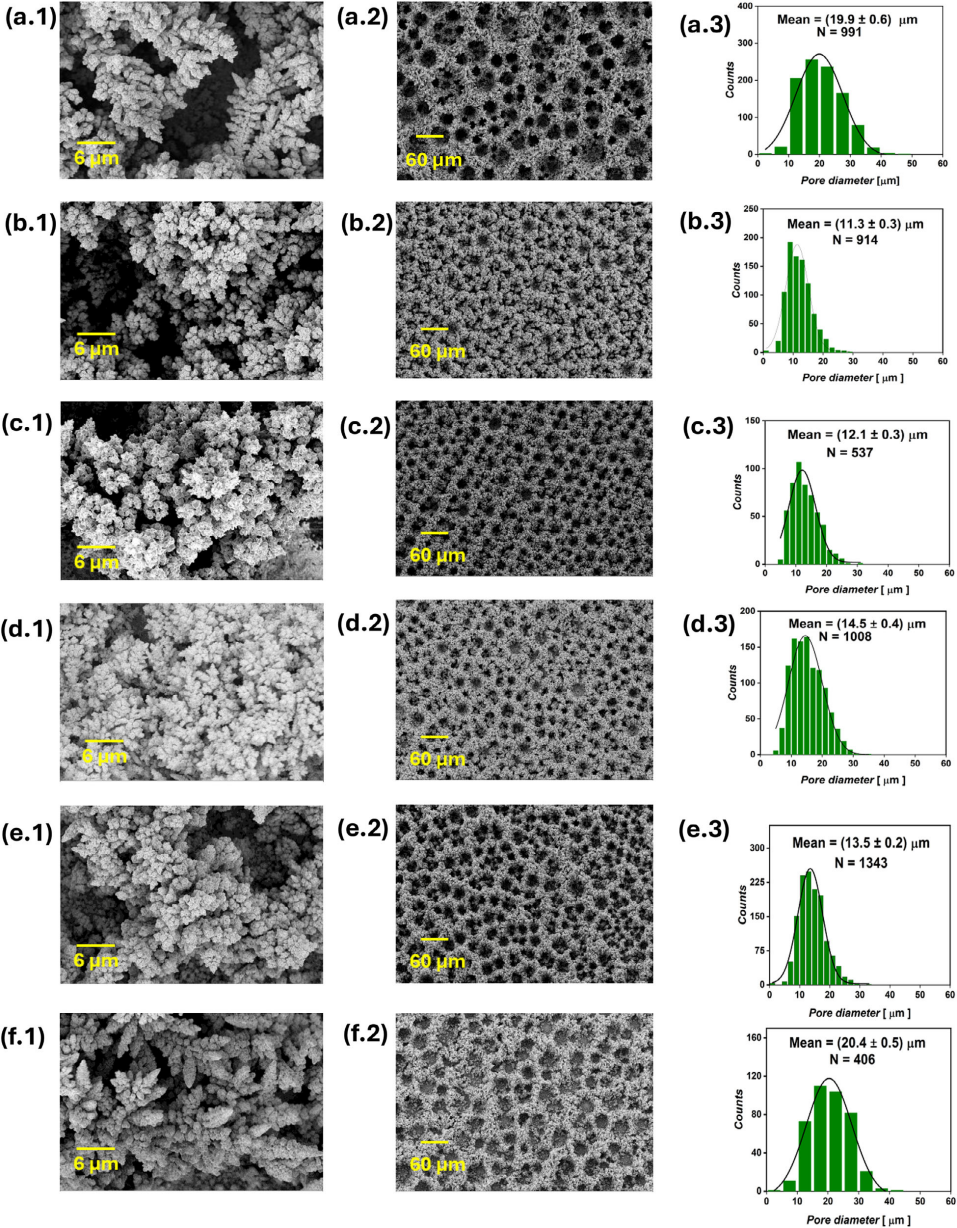
SEM images of the AC foams, showing the surface nano‐structures at 3000x magnification (first column), foam structure at 200x magnification (second column), and pore diameter distribution extracted from the 200x images (third column) of the DC reference sample a), ${\mathrm{AC}}_{1.2A}^{0.5\mathrm{Hz}}$ b), ${\mathrm{AC}}_{1.5A}^{0.5\mathrm{Hz}}$ c), ${\mathrm{AC}}_{2A}^{0.5\mathrm{Hz}}$ d), ${\mathrm{AC}}_{1.5A}^{1000\mathrm{Hz}}$ e), and ${\mathrm{AC}}_{1.5A}^{5000\mathrm{Hz}}$ f), where *N* is the number of pores from which the pore diameter distribution was obtained.

The impact of performing DHBT using an AC waveform superimposed on a DC offset was systematically investigated. Prior studies indicate that to produce AC–derived structures that differ markedly from those formed under a pure DC signal, the AC amplitude must exceed the DC offset.^[^ ^35^ ^]^ Guided by this criterion, five distinct AC‐DHBT conditions were explored. In all experiments, a cathodic DC current density of 1 A cm^−2^ was applied for 20 s, with the total charge density fixed at 20 C cm^−2^. The AC‐generated foams were compared to a Cu foam synthesized using a pure continuous cathodic DC signal of 1 A cm^−2^ applied for 20 s. Five AC foams were synthesized and labeled according to the applied AC amplitude and frequency as ${\mathrm{AC}}_{1.2A}^{0.5\mathrm{Hz}}$, ${\mathrm{AC}}_{1.5A}^{0.5\mathrm{Hz}}$, ${\mathrm{AC}}_{2A}^{0.5\mathrm{Hz}}$, ${\mathrm{AC}}_{1.5A}^{1000\mathrm{Hz}}$, and ${\mathrm{AC}}_{1.5A}^{5000\mathrm{Hz}}$ with corresponding applied cycle counts of 10, 10, 10, 20 000, and 100 000. **Figure** **7** and Figure S5 (Supporting Information) present the SEM analysis of the AC foams. Low‐magnification SEM images revealed that all AC samples exhibited honeycomb‐like structures. At a constant AC frequency of 0.5 Hz, increasing the AC amplitude resulted in larger pore diameters, with mean values of 11.3 ± 0.3, 12.1 ± 0.3, and 14.5 ± 0.4 µm for ${\mathrm{AC}}_{1.2A}^{0.5\mathrm{Hz}}$, ${\mathrm{AC}}_{1.5A}^{0.5\mathrm{Hz}}$, and ${\mathrm{AC}}_{2A}^{0.5\mathrm{Hz}}$ foams, respectively. When the AC amplitude was maintained constant at 1.5 A cm^−2^, raising the frequency from 0.5 to 5000 Hz increased the pore diameter from 12.1 ± 0.3 to 20.4 ± 0.5 µm. The measured thicknesses were 47.3 ± 3.8, 37.4 ± 2.0, 43.0 ± 3.1, 40.7 ± 5.5, and 44.0 ± 4.1 µm for ${\mathrm{AC}}_{1.2A}^{0.5\mathrm{Hz}}$, ${\mathrm{AC}}_{1.5A}^{0.5\mathrm{Hz}}$, ${\mathrm{AC}}_{2A}^{0.5\mathrm{Hz}}$, ${\mathrm{AC}}_{1.5A}^{1000\mathrm{Hz}}$, and ${\mathrm{AC}}_{1.5A}^{5000\mathrm{Hz}}$, correspondingly. The DC reference sample exhibited a mean pore diameter of 19.9 ± 0.6 µm and a thickness of 50.6 ± 5.3 µm. All AC samples, except ${\mathrm{AC}}_{1.5A}^{5000\mathrm{Hz}}$, exhibited higher pore densities than the DC reference. For example, the ${\mathrm{AC}}_{1.2A}^{0.5\mathrm{Hz}}$ foam showed a pore density of approximately 1379 pore mm^−2^, compared to about 748 pore mm^−2^ for the DC foam. Samples electrodeposited at AC frequencies of 0.5 and 1000 Hz exhibited fine surface nano‐dendrites, whereas the ${\mathrm{AC}}_{1.5A}^{5000}$ sample developed larger and more branched nano‐dendrites. Figure 3b displays the gravimetric analysis of AC Cu electrodeposits along with the corresponding calculated electrodeposition Faradaic efficiencies. The samples ${\mathrm{AC}}_{1.2A}^{0.5\mathrm{Hz}}$, ${\mathrm{AC}}_{1.5A}^{0.5\mathrm{Hz}}$, and ${\mathrm{AC}}_{2A}^{0.5\mathrm{Hz}}$ exhibited comparable electrodeposited mass densities of 2.5 ± 0.2, 2.5 ± 0.1, 2.4 ± 0.1 mg, corresponding to Faradaic efficiencies of 38 ± 1, 38 ± 2, and 37 ± 1 %, respectively. In comparison, the ${\mathrm{AC}}_{1.5A}^{1000\mathrm{Hz}}$, ${\mathrm{AC}}_{1.5A}^{5000\mathrm{Hz}}$, and DC samples showed higher mass densities of 3.0 ± 0.2, 3.1 ± 0.2 and 3.1 ± 0.3 mg cm^−2^, with Faradaic efficiencies of 45 ± 3, 47 ± 3, and 48 ± 4%, correspondingly.

All the AC samples showed significantly lower ECSA values of around 75 ${\mathrm{cm}}_{\mathrm{ECSA}}^{2}$.${\mathrm{cm}}_{\mathrm{geo}}^{-2}$ compared to the DC counterpart (127 ± 15 ${\mathrm{cm}}_{\mathrm{ECSA}}^{2}$.${\mathrm{cm}}_{\mathrm{geo}}^{-2}$), as shown in Figure 4b. The AC‐DHBT results indicate that, within the low to moderate frequency range (0.5–1000 Hz), the high current amplitudes reached during the cathodic half‐cycles emulate the effect of applying elevated current densities in DC mode, as shown in the current–time DHBT series. This leads to the formation of compact structures characterized by smaller pore diameters and higher pore densities. However, during AC‐DHBT, a portion of the applied charge is consumed by the charging and discharging processes of the electrode double‐layer capacitance.^[^ ^37^ ^]^ As capacitive contributions increase, the effective cathodic current density becomes attenuated. This attenuation mimics the effect of reducing DHBT currents in the DC current–time series, resulting in larger pore diameters and lower pore densities. Since capacitive effects become more pronounced with increasing AC signal amplitude or frequency, this can explain the gradual increase in pore diameter and corresponding decrease in pore density observed at higher AC parameters. Additionally, the formation of surface cauliflower‐like nano‐structures appears to require a sudden, periodic drop in cathodic current, as observed in the PC and RC samples. In contrast, the relatively smoother transition between cathodic and anodic regimes during AC‐DHBT generates hydrodynamic conditions similar to those in DC‐DHBT, resulting in nano‐dendrites. Again, the compact AC foams, in particular those obtained at 0.5–1000 Hz, can be promising candidates for electrocatalytic applications, such as CO_2_RR to valuable products.

### 2.3 Physical DHBT Effect Series

Various physical parameters were systematically investigated to assess their influence on the morphology of the DHBT‐synthesized Cu foams. To confirm the role of Cu^2+^ diffusion layer thickness on the resulting surface nano‐dendrites, the effect that was previously observed and discussed in the current–time and current mode series, a DHBT synthesis was conducted under vigorous electrolyte stirring at approximately 1350 revolution per minute (rpm) using a magnetic stirrer to reinforce the stirring effect at the electrode surface. To examine the impact of electrode orientation, a custom‐designed DHBT cell was fabricated to enable horizontal alignment of the working and counter electrodes with a collateral electrical connection (Figures S6 and S7, Supporting Information). The effect of electrolyte temperature was studied by performing DHBT at two controlled temperatures: 5 °C using an ice bath, and 60 °C using a heated water bath. Furthermore, the influence of post‐DHBT thermal treatment was explored under two annealing conditions: 300 °C for 15 min and 150 °C for 30 min. The two annealed samples were referred to as ${\mathrm{An}}_{15\min}^{300^{\circ }C}$ and ${\mathrm{An}}_{30\min}^{150^{\circ }C}$, respectively. All samples in this series were electrodeposited using a constant continuous cathodic current density of 1 A cm^−2^ for 20 s and were compared against a reference sample deposited under identical current density and time conditions, but at room temperature in a quiescent solution with vertical electrode alignment and without post‐deposition annealing, as described in detail in Section 5, and presented in Figure S8 (Supporting Information).

**Figure 8:**
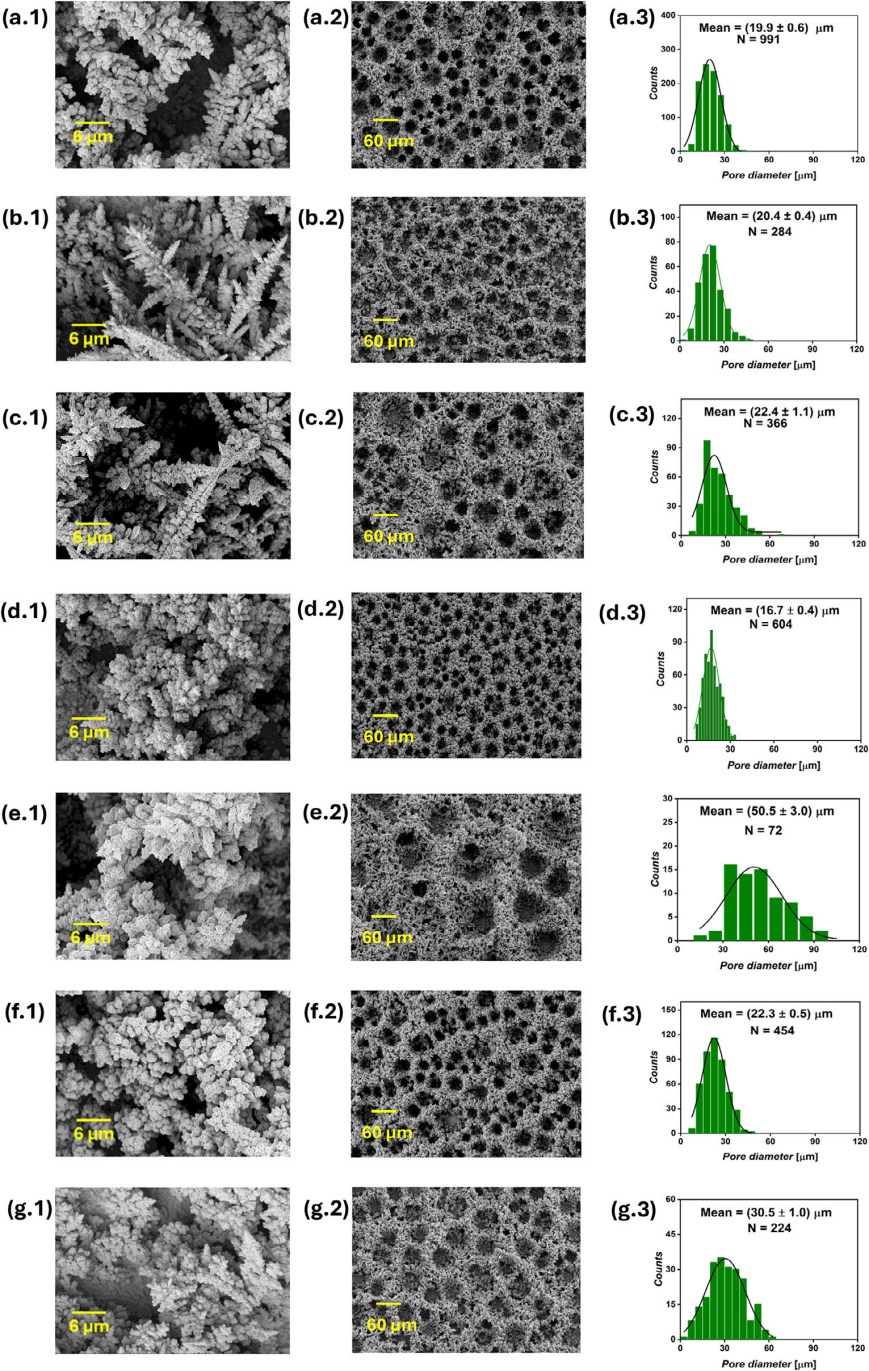
SEM images of the Physical DHBT effect series, showing the surface nano‐structures at 3000x magnification (first column), foam structure at 200x magnification (second column), and pore diameter distribution extracted from the 200x images (third column) for the reference sample a), stirred sample b), horizontally aligned sample c), sample produced at 5 °C d), sample produced at 60 °C e), sample annealed at 300 °C and 15 min f), and sample annealed at 150 °C and 30 min g), where *N* is the number of pores from which the pore diameter distribution was obtained.

Low‐ and high‐magnification top‐view SEM images of the foam structures resulting from variations in the physical parameters of the DHBT process are shown in **Figure** **8**, while the corresponding cross‐sectional SEM images are presented in Figure S9 (Supporting Information). Gravimetric analysis of the physical DHBT effect series is presented in Figure 3c. Introducing mechanical stirring to the DHBT bath had only a minor effect on the overall pore diameter and foam thickness compared to the reference sample. Specifically, the average pore diameters were 19.9 ± 0.6 and 20.4 ± 0.4 µm, and foam thicknesses were 50.6 ± 5.3 and 49.4 ± 5.3 µm, electrodeposited Cu masses were 3.1 ± 0.3 and 3.3 ± 0.3 mg cm^−2^, and deposition Faradic efficiencies were 48 ± 4 and 50 ± 4% for the reference and stirred samples, respectively. However, in contrast to the relatively unchanged foam skeleton, stirring the DHBT bath had a pronounced effect on increasing the surface nano‐dendrite size. This observation supports the hypothesis that enhanced mass transport of Cu^2+^ ions to the electrode surface, i.e., reducing the diffusion layer thickness, increases the limiting current density, decreases the electrodeposition overpotential, and promotes the fast growth of larger nano‐dendrite structures. In the quiescent DHBT process, the stirring effect arises only from the natural dynamics of hydrogen bubble formation and detachment, as observed in the previous DHBT series.

The horizontal alignment of electrodes results in a larger pore diameter (22.4 ± 1.1 µm), increased foam thickness (64.1 ± 8.1 µm), larger electrodeposited Cu mass density (4.4 ± 0.7 mg), corresponding electrodeposition Faradaic efficiency (66 ± 11%), and larger nano‐dendrite structures compared to the vertically aligned reference sample. This effect can be attributed to gravitational forces acting against the buoyant rise of hydrogen bubbles. As a result, larger bubbles must form and coalesce before detaching from the cathode surface. Again, it is clear that large H_2_ bubble size enhances local convection and facilitates the transport and flow of Cu^2+^ ions from the bulk electrolyte to the electrode. This increased ion flux promotes the growth of larger nano‐dendrites. These findings are consistent with previous studies, where horizontal placement of the working electrode during HER, a side reaction during the reduction of K_3_Fe(CN)_6_, led to improved mass transport of K_3_Fe(CN)_6_ to the electrode surface.^[^ ^38^ ^]^ In a simulation study investigating the evolution and growth of H_2_ bubbles on both vertical and horizontal micro‐electrodes,^[^ ^39^ ^]^ various forces, including contact pressure, surface tension, buoyancy, thermal Marangoni effects, hydrostatic pressure, and shear lift, were taken into account. The results showed that a significant increase in bubble diameter was required for detachment in the horizontal configuration compared to the vertical one, agreeing with the experimental DHBT findings in our work.

For the Cu foam samples electrodeposited at 5°C, room temperature, and 60 °C, a progressive increase in all morphological descriptors was observed. The average pore diameters were 16.7 ± 0.4, 19.9 ± 0.6, and 50.5 ± 3.0 µm, with foam thicknesses of 38.2 ± 4.1, 50.6 ± 5.3, and 69.6 ± 7.8 µm for the three samples, respectively. Similarly, the electrodeposited Cu mass densities were 2.6 ± 0.1, 3.1 ± 0.3, and 4.0 ± 0.3 mg cm^−2^, yielding Faradaic efficiencies of 39 ± 2, 48± 4, and 61 ± 4% at the three temperatures, correspondingly. These results suggest that increasing the DHBT bath temperature leads to larger hydrogen bubble templates, consistent with a previous study with the same findings, attributing this to enhanced HER rates at elevated temperatures.^[^ ^40^ ^]^ However, in our study, the observed increase in electrodeposition Faradaic efficiency with temperature indicates a reduction in concurrent HER activity. Therefore, the larger H_2_ bubble sizes can be attributed to temperature‐related reasons, such as reducing electrolyte viscosity at elevated temperatures, which promotes bubble coalescence. The nano‐dendrite size increased with increasing DHBT bath temperature, which can be attributed to both the stirring effect and reduced Cu electrodeposition overpotential at elevated temperatures. It is noteworthy that the increase in nano‐dendrite size at 60 °C occurred essentially via increasing the size of the dendrite needles. In contrast, under DHBT bath stirring and horizontal electrode alignment, the dendrite size increase primarily occurred along the branch axis. This indicates that enhanced stirring does not uniformly influence hydrodynamic conditions, as different physical parameters contribute to mass transport in distinct ways.

Before annealing, the mass densities of both samples designated for annealing were nearly identical to that of the reference sample (≈3.2 mg cm^−2^), as expected due to the use of identical DHBT parameters. Following annealing, both samples exhibited a slight mass density increase of ≈0.2 mg cm^−2^, likely due to the formation of Cu oxides during the thermal treatment in air. Additionally, annealing led to a noticeable expansion in the foam dimensions. The average pore diameters increased to 22.3 ± 0.5 and 30.5 ± 1.0 µm, while the foam thicknesses reached 71.7 ± 4.8 and 68.5 ± 3.7 µm for the ${\mathrm{An}}_{15\;\min}^{300^{\circ }C}$ and ${\mathrm{An}}_{30\;\min}^{150^{\circ }C}$ samples, respectively. In contrast to the expanded foam skeleton, the surface nano‐dendrites significantly decreased in size, which can be attributed to recrystallization processes induced by annealing.^[^ ^41^ ^]^

The ECSA results of the physical effect series are presented in Figure 4c. Horizontal alignment of the substrate during DHBT led to an increased ECSA 134 ± 2 ${\mathrm{cm}}_{\mathrm{ECSA}}^{2}$.${\mathrm{cm}}_{\mathrm{geo}}^{-2}$, compared to the reference sample (127 ± 15 ${\mathrm{cm}}_{\mathrm{ECSA}}^{2}$.${\mathrm{cm}}_{\mathrm{geo}}^{-2}$). A gradual increase in ECSA was also observed with increasing DHBT bath temperature, yielding values of 68 ± 7, 127 ± 15, and 155 ± 6 ${\mathrm{cm}}_{\mathrm{ECSA}}^{2}$.${\mathrm{cm}}_{\mathrm{geo}}^{-2}$ for samples prepared at 5 °C, room temperature, and 60 °C, respectively. Stirring the DHBT bath resulted in an ECSA of 114 ± 15 ${\mathrm{cm}}_{\mathrm{ECSA}}^{2}$.${\mathrm{cm}}_{\mathrm{geo}}^{-2}$. Annealing produced distinct effects on ECSA relative to the non‐annealed reference. The sample ${\mathrm{AC}}_{15\;\min}^{300^{\circ }C}$ exhibited an increased ECSA of 167 ± 8 ${\mathrm{cm}}_{\mathrm{ECSA}}^{2}$.${\mathrm{cm}}_{\mathrm{geo}}^{-2}$, while the sample ${\mathrm{AC}}_{30\;\min}^{150^{\circ }C}$ showed a reduced ECSA of 81 ± 15 ${\mathrm{cm}}_{\mathrm{ECSA}}^{2}$.${\mathrm{cm}}_{\mathrm{geo}}^{-2}$. The difference in ECSA between the annealed samples arises from the predominant microstructural mechanisms triggered by temperature. At 150 °C, partial surface recrystallization can occur, resulting in surface smoothing and reduction of active site density. At 300 °C, complete surface recrystallization and reconstruction take place, increasing surface defects, the number of active sites, and the overall surface area.^[^ ^42^ ^]^

### 2.4 Chemical DHBT Effect Series

In this series, the effect of Cu^2+^ concentration, electrolyte pH, adding surfactants, such as sodium dodecane‐1‐sulfonate (commonly known as sodium dodecyl sulfate, SDS), and hexadecyltrimethylazanium bromide (commonly known as cetyltrimethylammonium bromide, CTAB), and adding Cu‐complexing agents, such as 3,5‐diamino‐1,2,4‐triazole (DAT) was investigated. All samples were electrodeposited at a continuous cathodic current density of 1 A cm^−2^ applied for 20 s, and referenced to a sample produced at the same current density and time but from the standard electrolyte mentioned in Section 5.

**Figure 9:**
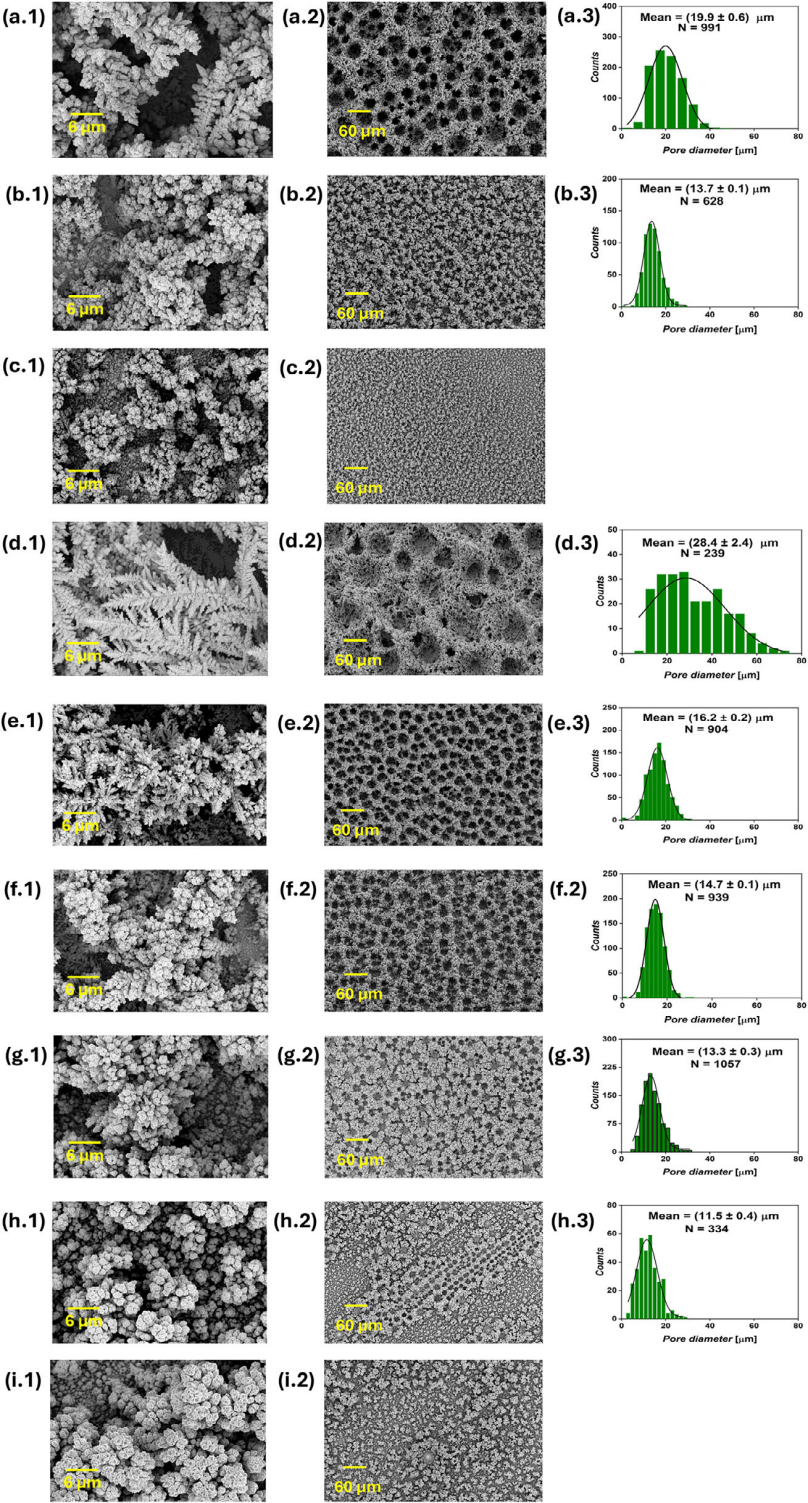
SEM images of the Chemical DHBT effect series, showing the surface nano‐structures at 3000x magnification (first column), foam structure at 200x magnification (second column), and pore diameter distribution extracted from the 200x images (third column) of the reference sample a), samples prepared at Cu^2+^ concentrations of 0.1 M b) and 0.05 M c), sample electrodeposited at pH 0.5 d), samples synthesized in the presence of 0.25 mM of CTAB e) and SDS f), samples synthesized in the presence of DAT at concentrations of 1.5 mM g) and 4 mM h), and sample prepared with 4mM DAT at pH 0.5 (i), where *N* is the number of pores from which the pore diameter distribution was obtained. For the samples without a displayed pore diameter distribution, there were insufficient pore numbers to generate a reliable distribution.

SEM analysis (**Figure** **9** and Figure S10, Suppporting Information) shows that reducing Cu^2+^ concentration from 0.2 to 0.1 M or 0.05 M produced non‐uniform Cu agglomerates with reduced interconnectivity and smaller nano‐dendrites. The sample thickness decreased correspondingly from 50.6 ± 5.3 µm (0.2 M Cu^2+^) to 30.3 ± 2.0 µm (0.1 M Cu^2+^) and 22.9 ± 10.2 (0.05 M Cu^2+^). The average pore diameter at 0.1 M (13.7 ± 0.1 µm) was significantly lower than that of the reference sample (19.9 ± 0.6 µm). Conversely, increasing electrolyte pH from ‐0.5 (for the reference sample) to 0.5 yielded larger foam thickness (65.5 ± 6.9 µm), pore diameter (28.4 ± 2.4 µm), and nano‐dendrite size. The relatively broader pore diameter distribution of the pH 0.5 sample indicates a lower pore uniformity for this sample. Gravimetric analysis Figure 3d revealed that the Cu mass density and electrodeposition Faradaic efficiency decreased with lowering Cu^2+^ concentrations from 3.1 ± 0.3 mg cm^−2^ and 48 ± 4% (reference) to 1.6 ± 0.1 mg cm^−2^ and 25 ± 1% (0.1 M Cu^2+^) and 0.7 ± 0.05 mg cm^−2^ and 10 ± 1% (0.05 M Cu^2+^) but increased to 3.8 ± 0.2 mg cm^−2^ and 58 ± 4% by elevating the electrolyte pH from about ‐0.5 to 0.5. These results indicate enhanced H_2_ evolution at lower Cu^2+^ concentrations, indicated by the lower electrodeposition Faradaic efficiency, creating numerous H_2_ bubbles which possess a very small size, providing minimal stirring, producing smaller nano‐dendrites, and eliminating the intended interconnected foam structure. At pH 0.5, reduced H_2_ evolution, indicated by higher electrodeposition Faradaic efficiency, generates fewer, larger bubbles that create stronger stirring effects and larger nano‐dendrites. Importantly, the emergence of the Cu honeycomb‐like structure exclusively at the highest Cu^2 +^ concentration supports the assumption that achieving interconnected foam structures via DHBT requires sufficiently high rates of both Cu electrodeposition and HER, given the dependence of reaction rates on reactant concentrations.^[^ ^5^ ^]^ Furthermore, despite a tenfold decrease in H^+^ concentration from 3 to 0.3 M, which was performed to raise the DHBT electrolyte pH from approximately ‐0.5 to 0.5, the foam structure persisted. This observation corroborates the assumption that, during DHBT, HER can proceed via the reduction of both H^+^ ions and water molecules, as described by Equations (5) and (6)^[^ ^5^ ^]^

(5) $$2H_{2}O+2e^{-}⟶H_{2}\left(g\right)+2{\mathrm{OH}}^{-}$$

(6) $$2H^{+}+2e^{-}⟶H_{2}\left(g\right)$$

However, the increase in the electrodeposition Faradaic efficiency, pore diameter, and dendrite size at pH 0.5 indicates that a hindrance in HER at lower proton concentrations still exists.

The addition of either SDS or CTAB at a concentration of 0.25 mM to the DHBT bath resulted in a significant structural transformation. Compared to the additive‐free reference sample, surfactants reduced the average pore diameter and foam thickness to 16.2 ± 0.2 and 30.5 ± 1.6 µm for CTAB, and 14.7 ± 0.1 and 33.3 ± 2.6 µm for SDS, respectively. The pore densities exhibited by the SDS‐ (1063 pore mm^−2^) and CTAB‐meditated (1364 pore mm^−2^) samples were significantly higher than the reference counterpart (748 pore mm^−2^). Additionally, the electrodeposited Cu mass density and Faradaic efficiency decreased to 1.7 ± 0.1 mg cm^−2^ and 26 ± 1% for CTAB, and to 1.4 ± 0.1 mg cm^−2^ and 22 ± 2% for SDS. These results are consistent with the known effect of surfactants in lowering electrolyte surface tension, which promotes HER and leads to smaller H_2_ bubble sizes, increased bubble density, and reduced electrodeposited mass density, as illustrated schematically in Figure S11 (Supporting Information).^[^ ^43^ ^]^ The high compactness and small surface nano‐dendrites of the surfactant‐mediated Cu foams support the fact that increased HER rates lead to smaller bubble templates and reduced local stirring. Interestingly, although both SDS and CTAB reduced nano‐dendrite sizes, their resulting morphologies differed significantly. The dendrites of the CTAB‐mediated sample are more branched with numerous small and well‐defined dendrite needles compared to the nano‐dendrites of the SDS‐mediated counterpart. This suggests that SDS and CTAB influence Cu^2 +^ diffusion dynamics differently, though both slow down the transport from the bulk electrolyte to the electrode surface.

In contrast to surfactants, DAT caused less significant changes in the electrodeposited Cu mass and electrodeposition Faradaic efficiency compared to the additive‐free sample, indicating a limited impact on the amount of H_2_ evolution. However, remarkable morphological changes were observed. At a DAT concentration of 1.5 mM, for the sample denoted as DAT_1.5 mM_, the mean pore diameter and foam thickness decreased to 13.3 ± 0.3 and 17.3 ± 2.0 µm, respectively, both smaller than those of the additive‐free reference, with cracks within the DAT‐mediated sample walls. When the DAT concentration was increased to 4 mM, for the sample denoted as DAT_4mM_, only isolated foam islands were formed, with a mean pore diameter of 11.5 ± 0.4 µm, and a sample thickness of 16.6 ± 4.1 µm. Further, when the electrolyte pH was raised to 0.5 at 4 mM DAT, for the sample referred to as ${\mathrm{DAT}}_{4\mathrm{mM}}^{\mathrm{pH}\;0.5}$, the foam structure disappeared entirely, and only Cu agglomerates were observed. This agrees with previous studies showing that the effect of DAT as a Cu‐complexing agent becomes more pronounced at higher pH values.^[^ ^44^ ^]^ At 1.5 mM DAT, smaller nano‐dendrites were observed, whereas the samples DAT_4mM_ and ${\mathrm{DAT}}_{4\mathrm{mM}}^{\mathrm{pH}\;0.5}$ exhibited cauliflower‐like nano‐structures. This suggests that DAT complexation influences Cu electrodeposition through a mechanism similar to the abrupt, periodic cathodic interruptions observed in the PC and RC DHBT regimes. The ability to tailor Cu foam dimensions and nano‐structures using additives such as DAT (at low concentrations), SDS, and CTAB renders the resulting Cu foams promising candidates for electrocatalytic applications, including CO_2_RR, which is one of the key focuses of our current work. Moreover, for applications where foam structures are unnecessary and binder‐free nano‐particles of different shapes are preferred, samples produced at 4 mM DAT or 0.05 M Cu^2+^ concentrations present ideal candidates.

The ECSA results of the chemical effect series are shown in Figure 4d. The reference sample exhibited an ECSA of 127 ± 15 ${\mathrm{cm}}_{\mathrm{ECSA}}^{2}$.${\mathrm{cm}}_{\mathrm{geo}}^{-2}$, which declined upon the addition of any investigated additive to 52 ± 3 ${\mathrm{cm}}_{\mathrm{ECSA}}^{2}$.${\mathrm{cm}}_{\mathrm{geo}}^{-2}$ (at 1.5 mM DAT), 44 ± 5 ${\mathrm{cm}}_{\mathrm{ECSA}}^{2}$.${\mathrm{cm}}_{\mathrm{geo}}^{-2}$ (at 4 mM DAT and pH‐0.5), 69 ± 8 ${\mathrm{cm}}_{\mathrm{ECSA}}^{2}$.${\mathrm{cm}}_{\mathrm{geo}}^{-2}$ (at 4 mM DAT and pH0.5), 49 ± 11 ${\mathrm{cm}}_{\mathrm{ECSA}}^{2}$.${\mathrm{cm}}_{\mathrm{geo}}^{-2}$ (SDS), and 68 ± 8 ${\mathrm{cm}}_{\mathrm{ECSA}}^{2}$.${\mathrm{cm}}_{\mathrm{geo}}^{-2}$ (CTAB). Lowering the Cu^2+^ concentration also reduced the ECSA to 51 ± 6 ${\mathrm{cm}}_{\mathrm{ECSA}}^{2}$.${\mathrm{cm}}_{\mathrm{geo}}^{-2}$ (0.1 M) and 31 ± 3 ${\mathrm{cm}}_{\mathrm{ECSA}}^{2}$.${\mathrm{cm}}_{\mathrm{geo}}^{-2}$ (0.05 M). In contrast, increasing the DHBT electrolyte pH from ‐0.5 to 0.5 resulted in an ECSA comparable to the reference sample of 125 ± 5 ${\mathrm{cm}}_{\mathrm{ECSA}}^{2}$.${\mathrm{cm}}_{\mathrm{geo}}^{-2}$.

The morphology descriptors investigated in this work for all the studied model Cu foam samples are summarized in **Table** **1**, and a brief presentation of the effect of individual DHBT parameters on the Cu foam morphology is provided in Table S1 (Supporting Information). The relationship between the foam thickness and pore diameter is provided in Figure S12 (Supporting Information), excluding the annealed samples, as they possess morphological changes unrelated to DHBT conditions. A clear linear correlation between the two parameters is observed within the pore diameter range of ≈10–30 µm. This suggests that, within this regime, the underlying nature of the DHBT process remains consistent. The role of the different DHBT parameters appears to be essentially governing the extent to which the process proceeds, rather than altering its fundamental nature. Beyond 30 µm, the relationship deviates from linearity for the (1 A cm^−2^ 30 s) sample and the sample prepared at 60 °C, potentially indicating a change in the DHBT behavior at larger bubble size or the presence of outliers due to experimental uncertainties.

**Table 1:** Summarized morphology results of the Cu foam samples synthesized in this study: pore diameter, thickness, pore density, mass density, and ECSA (measured via CV).

Sample	Diameter [µm]	Thickness [µm]	Pore density [pore mm^−2^]	Mass density [mg cm^−2^]	ECSA
1Acm^−2^ 10s	11.5 ± 0.3	24.8 ± 1.7	1824	1.5 ± 0.1	41 ± 1
1Acm^−2^ 20s, DC, Ref	19.9 ± 0.6	50.6 ± 5.3	748	3.1 ± 0.3	127 ± 15
1Acm^−2^ 30s	36.7 ± 0.5	62.0 ± 4.7	346	4.8 ± 0.2	179 ± 12
2Acm^−2^ 5s	9.4 ± 0.1	23.0 ± 1.5	3541	1.2 ± 0.1	41 ± 7
2Acm^−2^ 10s	15.5 ± 0.1	26.2 ± 2.4	1360	2.4 ± 0.2	85 ± 13
2Acm^−2^ 15s	20.1 ± 0.2	55.6 ± 4.6	806	3.8 ± 0.2	132 ± 12
PC_1:10_	–	19.7 ± 3.7	–	4.7 ± 0.1	39 ± 2
PC_5:10_	–	23.8 ± 5.3	–	3.8 ± 0.2	45 ± 2
PC_20:10_	17.4 ± 0.4	46.2 ± 5.2	631	3.4 ± 0.1	56 ± 6
PC_20:40_	–	25.7 ± 3.5	–	3.6 ± 0.1	37 ± 4
PC_20:200_	–	19.0 ± 1.6	–	4.4 ± 0.1	28 ± 3
RC_1mC_	15.7 ± 0.2	39.1 ± 2.1	733	3.4 ± 0.1	53 ± 5
RC_3mC_	15.0 ± 0.1	29.4 ± 9.1	588	3.3 ± 0.1	43 ± 2
RC_6mC_	13.1 ± 0.1	24.8 ± 5.3	936	3.1 ± 0.1	37 ± 1
${\mathrm{AC}}_{1.2A}^{0.5\mathrm{Hz}}$	11.3 ± 0.3	47.3 ± 3.8	1379	2.5 ± 0.2	79 ± 11
${\mathrm{AC}}_{1.5A}^{0.5\mathrm{Hz}}$	12.1 ± 0.3	37.4 ± 2.0	1215	2.5 ± 0.1	82 ± 8
${\mathrm{AC}}_{2}^{0.5\mathrm{Hz}}$	14.5 ± 0.4	43.0 ± 3.1	913	2.4 ± 0.1	76 ± 12
${\mathrm{AC}}_{1.5A}^{1000\mathrm{Hz}}$	13.5 ± 0.2	40.7 ± 5.5	1013	3.0 ± 0.2	71 ± 6
${\mathrm{AC}}_{1.5A}^{5000\mathrm{Hz}}$	20.4 ± 0.5	44.0 ± 4.1	613	3.1 ± 0.2	77 ± 11
5 °C	16.7 ± 0.4	38.2 ± 4.1	911	2.6 ± 0.1	68 ± 7
60 °C	50.5 ± 3.0	69.6 ± 7.8	54	4.0 ± 0.3	155 ± 6
Stirred	20.4 ± 0.4	49.4 ± 5.3	430	3.3 ± 0.3	114 ± 15
Horizontal	22.4 ± 1.1	64.1 ± 8.1	276	4.4 ± 0.7	134 ± 2
${\mathrm{An}}_{15\min}^{300^{\circ }C}$	22.3 ± 0.5	71.7 ± 4.8	514	3.1 ± 0.1	167 ± 8
${\mathrm{An}}_{30\min}^{150^{\circ }C}$	30.5 ± 1.0	68.5 ± 3.7	253	3.2 ± 0.1	81 ± 15
SDS	14.7 ± 0.1	33.3 ± 2.6	1063	1.4 ± 0.1	49 ± 11
CTAB	16.2 ± 0.2	30.5 ± 1.6	1364	1.7 ± 0.1	68 ± 8
0.1 M Cu^2 +^	13.7 ± 0.1	30.3 ± 2.0	948	1.6 ± 0.1	51 ± 6
0.05 M Cu^2 +^	–	22.9 ± 10.2	–	0.7 ± 0.05	31 ± 3
DAT_1.5mM_	13.3 ± 0.3	17.3 ± 2.0	797	2.9 ± 0.1	52 ± 3
DAT_4mM_	11.5 ± 0.4	16.6 ± 4.1	302	2.9 ± 0.2	44 ± 5
${\mathrm{DAT}}_{4\mathrm{mM}}^{\mathrm{pH}0.5}$	–	26.4 ± 5.2	–	3.3 ± 0.2	69 ± 8
pH 0.5	28.4 ± 2.4	65.5 ± 6.9	216	3.8 ± 0.2	125 ± 5

### 2.5 X‐Ray Analysis of the Model Cu Foam Samples

**Figure 10:**
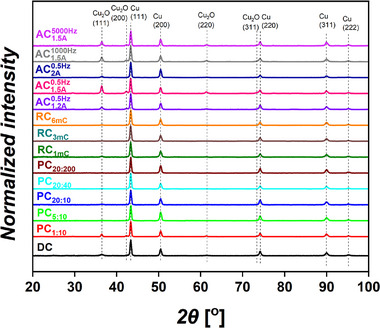
XRD patterns of the DHBT current mode series.

To investigate the crystal structure of the Cu foams, X‐ray diffraction (XRD) analysis was conducted. The Cu foams reported in this study exhibited thicknesses of up to ≈70 µm and were electrodeposited on Cu foils of 100 µm thickness, Therefore, the contribution of the substrate to the XRD results cannot be entirely excluded, since XRD is a bulk technique. Although the XRD pattern of the Cu substrate is remarkably different from the XRD patterns of the Cu foams (Figure S13, Supporting Information), XRD data need to be interpreted with caution. **Figure** **10** represents the XRD patterns of the current mode series. The XRD results of the other DHBT series are presented in Figures S13– S15 (Supporting Information).

All samples primarily exhibited metallic face‐centered cubic (fcc) Cu as the dominant phase, accompanied by a minor and distinct simple cubic Cu_2_O phase, likely caused by oxidative interaction with air after synthesis. No clear evidence of a CuO phase was detected by XRD. The Williamson–Hall method was employed to estimate the crystallite size and micro‐strain of the Cu foam samples. Instrumental broadening was evaluated using the XRD pattern of a LaB_6_ standard, and Equation (7) was applied to correct the measured broadening (β_*measured*_) for instrumental contributions (β_*instrumental*_), yielding the corrected broadening (β_*corrected*_). Subsequently, the Williamson–Hall plots were constructed based on Equation 8, where θ is the Bragg angle, *K* is the shape factor (typically 0.9), *L* is the crystallite size, λ is the X‐ray wavelength, and ϵ is the micro‐strain in the crystal lattice.^[^ ^45^ ^]^ The resulting crystallite size and micro‐strain values are summarized in Table S2 (Supporting Information).

In the current–time series, crystallite sizes ranged from 41 nm (1A cm^−2^/30 s) to 139 nm (2 Acm^−2^/5 s), and micro‐strain values ranged from 0.7 × 10^−3^ (2Acm^−2^/15s) to 1.7 × 10^−3^ ((1Acm^−2^/20s and (2Acm^−2^/5s)), with fcc Cu as the main phase. In the current mode series, relatively larger crystallite sizes were observed in PC samples deposited with small pulse‐to‐pause ratios. The smallest crystallite size (29 nm) was observed from RC_1mC_ sample, while other RC samples showed slightly larger sizes of 37 (RC_3 mC_) and 60 nm (RC_3 mC_). Micro‐strain values in the current mode series ranged from 0.5 × 10^−3^ (RC_1 mC_) to 1.8 × 10^−3^ (${\mathrm{AC}}_{1.5A}^{1000\mathrm{Hz}}$). Generally, AC samples exhibited relatively strong Cu_2_O reflections while still maintaining fcc Cu as the dominant phase. In the physical DHBT effect series, crystallite sizes ranged from 36 nm (${\mathrm{An}}_{30\;\min}^{150^{\circ }C}$) to 99 nm (60 °C), and the micro‐strain values from 0.3 × 10^−3^ (${\mathrm{An}}_{30\;\min}^{150^{\circ }C}$) to 1.3 × 10^−3^ (stirring‐modified sample). The 60 °C and ${\mathrm{An}}_{30\min}^{150^{\circ }C}$ samples exhibited the strongest Cu_2_O (111) reflections, although fcc Cu remained the dominant phase. In the Chemical DHBT effect series, DAT samples exhibited significantly smaller crystallite sizes and micro‐strain values. The negative micro‐strain values observed in the DAT samples have been previously reported in the literature for Al–Zn–0.4%Cu alloys and were attributed to compressive strain effects.^[^ ^46^ ^]^ In another study, negative micro‐strain has been interpreted as an indication that micro‐strain is either absent or has a minor contribution to peak broadening.^[^ ^47^ ^]^

(7) $$\beta_{corrected}^{2}=\beta_{measured}^{2}-\beta_{instrumental}^{2}$$

(8) $$\cos(\theta )\cdot \beta_{corrected}=\frac{K\cdot \lambda }{L}+4\varepsilon \cdot \sin(\theta )$$

X‐ray photoelectron spectroscopy (XPS) analysis was conducted on the as‐deposited Cu foam samples to reveal surface species and oxidation states. The O 1s spectra (Figure S16, Supporting Information) revealed prominent oxide‐related peaks for the Cu foam samples, with binding‐energy maxima between 531.2 and 529.5 eV, attributable to overlapping contributions from lattice and defective oxygen in Cu_2_O and CuO surface species. Notably, both DAT‐ and CTAB‐modified samples and the sample annealed at 300 °C exhibited increased overall oxide peak intensities relative to the other samples.

The Cu 2p spectra (Figure S17, Supporting Information) displayed principal peaks at binding energies of 932.2 eV (2p3/2) and 952.0 eV (2p1/2) for most samples, consistent with overlapping signals from metallic Cu and Cu_2_O and indicating the predominance of metallic copper and/or cuprous oxide phases at the surface. The DAT‐ and CTAB‐mediated samples showed additional, less intense shoulders at binding energies of 934.4 and 954.0 eV, consistent with CuO, and correlate with their enhanced oxide intensity in the O 1s region. These two samples also featured prominent shake‐up satellite features in the binding energy ranges of about 938–945 and 959–966 eV, diagnostic of surface CuO. The ${\mathrm{An}}_{15\;\min}^{300^{\circ }C}$ sample exhibited the opposite behavior, with principal peaks at 933.7 and 954.0 eV, from CuO, less intense shoulders at 932.2 and 952.0 eV from metallic and Cu_2_O, and very intense shake‐up satellite peaks, reflecting that annealing at 300 °C led to intensified surface oxidation to CuO. The Cu LMM Auger spectra (Figure S18, Supporting Information) showed a kinetic‐energy maximum at 917.0 eV for most samples, indicating the surface dominance of Cu_2_O, whereas the ${\mathrm{An}}_{15\min}^{300^{\circ }C}$ sample exhibited a maximum at a kinetic energy of 917.7 eV, confirming surface‐dominant CuO phase for that sample. The N 1s spectrum of the DAT‐deposited sample displayed a peak at 399.7 eV binding energy, which was absent in the reference additive‐free sample, attributed to superimposed contributions from amino and triazole nitrogen atoms from surface‐bound DAT molecules (Figure S19, Supporting Information). No Br, S, or Na signals were observed for the CTAB‐ and SDS‐mediated samples.

## 3 Application‐Driven DHBT Design of Cu Foam Structures

**Figure 11:**
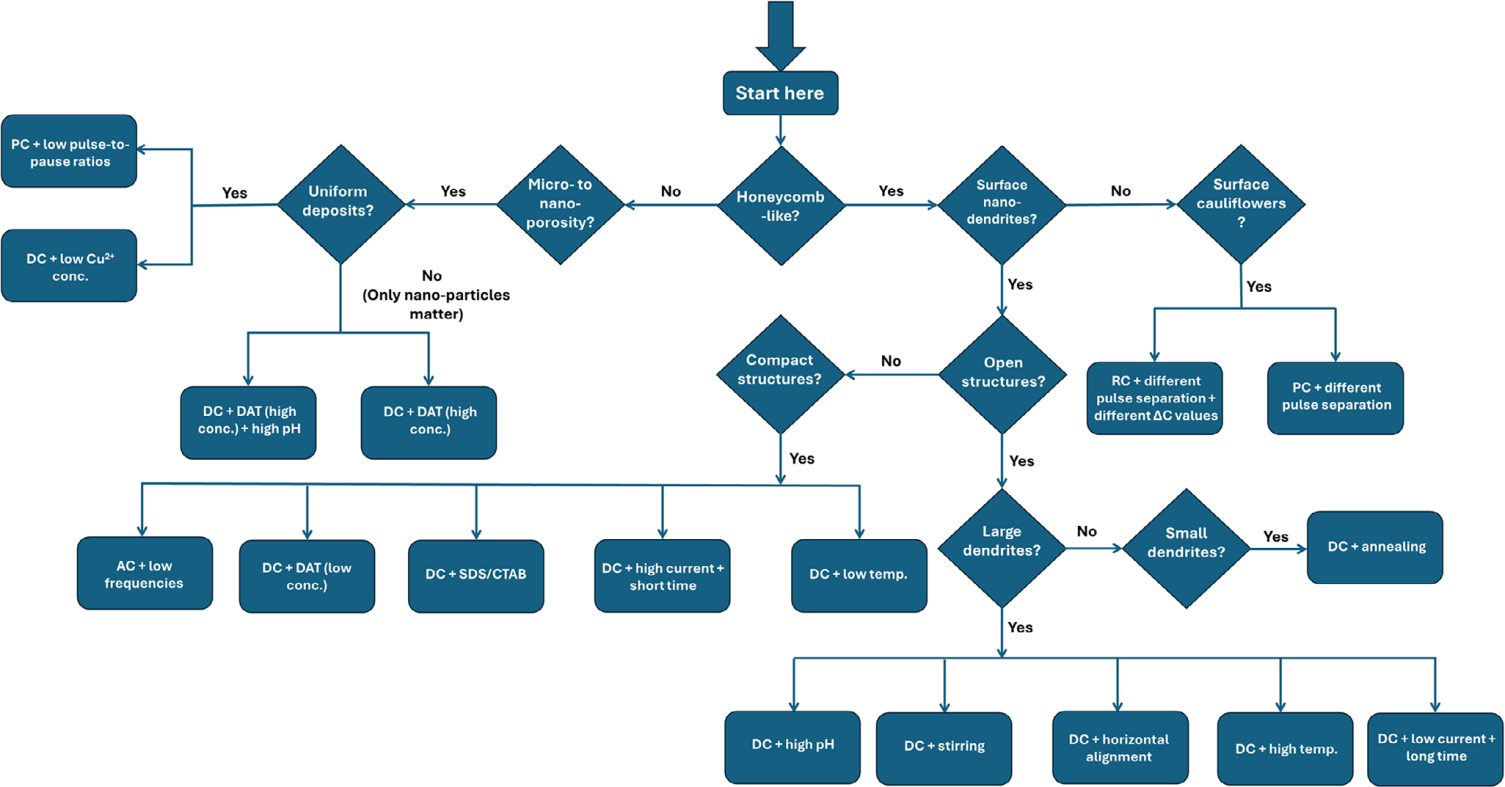
Flow chart for guiding the synthesis of Cu foam samples with tailored properties, based on the results obtained in this work.

**Figure** **11** presents a flow chart that guides the synthesis of Cu foams tailored to specific structural and compositional requirements based on the results of this work. The chart begins by distinguishing between two primary target morphologies: honeycomb‐like structures and only meso‐ to nano‐porous structures. For honeycomb‐like structures, the next decision point concerns the desired surface morphology, nano‐dendrites or nano‐cauliflowers. If cauliflower‐like nano‐structures are preferred, both RC and PC modes can be applied using higher pulse‐to‐pause ratios for PC or higher anodic‐to‐cathodic pulse ratios for RC. Additionally, a gradual control of pore diameter and foam thickness can be achieved by controlling the Δ*C* value in the RC mode. If nano‐dendrites are desired, the chart prompts the user to select either an open or compact desired foam structure. Open structures can be achieved using the DC mode, combined with long deposition times, low current densities, elevated temperatures, stirring (to promote dendrite growth, if large dendrites are targeted), and/or higher pH. The pH range studied in this work is between ‐0.5 and 0.5. If open foam structures are to be combined with small nano‐dendrites, post‐synthesis annealing can be applied. For compact metallic Cu foams, options include using the DC mode with low temperature, high current densities, short deposition times, and/or additives such as DAT (at low concentrations), SDS, or CTAB. One surfactant concentration of 0.25 mM and two DAT concentrations of 1.5 and 4 mM were investigated in this study. Alternatively, the AC mode at low frequencies can also be employed. If only meso‐ to nano‐porosity is desired, the chart asks whether uniformity is required. If uniformity is not critical, DAT at high concentrations with or without elevated pH can be used. If sample uniformity is essential, PC mode with low pulse‐to‐pause ratios or DC mode at low Cu^2+^ concentration is advised. It is important to note that these recommendations serve as guidelines only, not fixed protocols. A variability between laboratories was observed in the structure of the obtained Cu foam samples, which may stem from differences in potentiostat models, Cu foil preparation and etching procedures, as well as Cu foil suppliers. Therefore, while the flow chart offers a directional framework, systematic optimization under the onsite experimental conditions is essential. We strongly advise consulting the other Results and Discussion sections and Experimental section before following the flow chart. Suppose the goal is to synthesize an open, honeycomb‐like Cu foam with large nano‐dendrites, pores, and thickness, to be employed as a current collector for battery applications. Accordingly, following the flow chart: honeycomb‐like? → yes, surface nano‐dendrites? → yes, open structure? → yes, large dendrites? → yes, the chart recommendations include using the DC mode combined at low current densities, with the possibility to control the foam thickness and pore diameter simply via electrodeposition times.

### 3.1 DHBT‐Derived Cu Foam Gas Diffusion Electrodes for CO_2_ Reduction

To demonstrate the feasibility and operational simplicity of the DHBT method for fabricating electrodes suitable for energy applications, a streamlined three‐step protocol was developed for the synthesis of Cu foam gas diffusion electrodes (GDEs) for CO_2_RR. This approach was inspired by a previously reported procedure for Ag foam GDEs synthesis.^[^ ^3^ ^]^ Initially, PTFE/PP bilayer fabric substrates were sputter‐coated on the PTFE side with a 480 nm‐thick Ag layer. Subsequently, the DHBT process was employed to generate porous Cu foam structures directly on the Ag‐coated substrates. In the final step, ionomer infiltration was performed to impart hydrophobicity to the Cu foam GDEs, as described in detail and represented schematically in Section 5.

Six Cu foam GDE samples were synthesized to systematically investigate the influence of electrodeposition parameters and additives on the GDEs morphology and CO_2_RR performance. First, to compare the DC and PC DHBT modes, two electrodes were fabricated at a cathodic charge density of 30 C cm^−2^, one using DC and the other using PC, and were abbreviated as ${\mathrm{DC}}_{30C\;{\mathrm{cm}}^{-2}}$ and ${\mathrm{PC}}_{30C\;{\mathrm{cm}}^{-2}}$. Second, to assess the impact of charge density under PC conditions, two additional samples were prepared at charge densities of 60 and 90 C cm^−2^, referred to as ${\mathrm{PC}}_{60C\;{\mathrm{cm}}^{-2}}$ and ${\mathrm{PC}}_{90C\;{\mathrm{cm}}^{-2}}$. Third, the role of DAT was explored by incorporating 4 mM of DAT into the DHBT bath, referring to this sample as ${\mathrm{PC}}_{30C\;{\mathrm{cm}}^{-2}}^{\mathrm{DAT}}$. Finally, to evaluate the effect of lowering the DHBT bath temperature, a GDE was synthesized under the PC mode at a charge density of 30 C cm^−2^ at 4 °C using an ice bath, and was denoted as 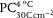.

**Figure 12:**
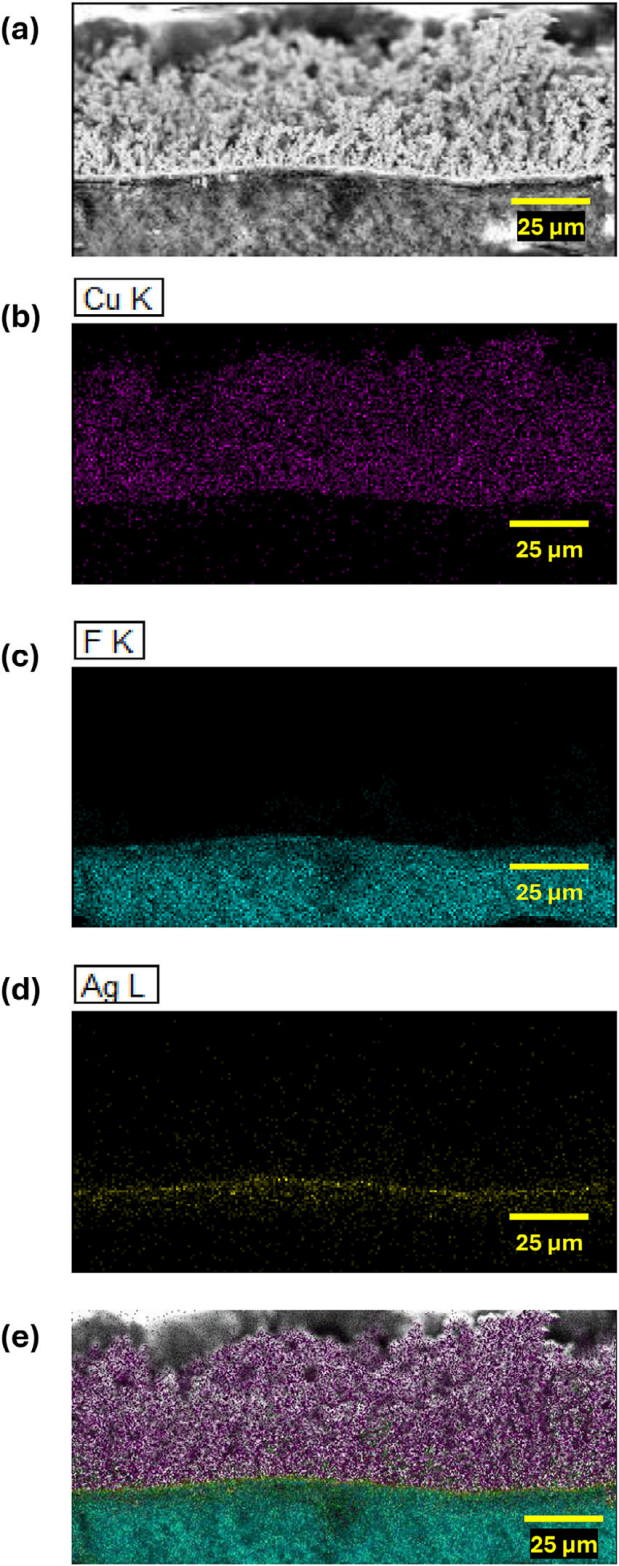
Cross‐sectional EDX elemental mapping of a Cu foam GDE, showing the distribution of Cu (catalytic layer), Ag (sputtered conductive interlayer), and F from the PTFE fabric substrate.

**Figure** **12** presents an energy‐dispersive X‐ray spectroscopy (EDX) cross‐sectional mapping of a Cu foam GDE synthesized via the described protocol before ionomer infiltration. The figure illustrates the stratified architecture comprising the Cu foam layer (top), the sputtered Ag interlayer (middle), and the underlying PTFE substrate (bottom), where PTFE was indicated via the fluorine signal. The successful transformation of DHBT‐derived model Cu foams (of 1 cm^−2^ geometric area) discussed earlier in this manuscript to GDEs, and the reproducibility of Cu foam formation on Ag‐sputtered PTFE, underscore the versatility and scalability of the DHBT method. Notably, the method demonstrates effectiveness not only on planar substrates but also on porous supports, which are highly relevant for energy storage and conversion applications. In the resulting GDE architecture, the Cu foam functions as the catalytic layer, while the PTFE and PP fabric layers serve as the micro‐ and macro‐porous diffusion layers, respectively.

The hydrophobicity of the Cu foam GDEs after ionomer infiltration was evaluated via contact angle measurements. Figure S20 (Supporting Information) displays representative contact angle images of the DC_30Ccm_^−2^ sample before and after ionomer treatment. Prior to infiltration, the sample exhibited complete wettability, with the water droplet immediately absorbed by the Cu foam. In contrast, the ionomer‐treated sample demonstrated a pronounced stable hydrophobic behavior, with a contact angle of ≈125°, confirming the GDE suitability for operation in CO_2_RR flow cell configurations.

**Figure 13:**
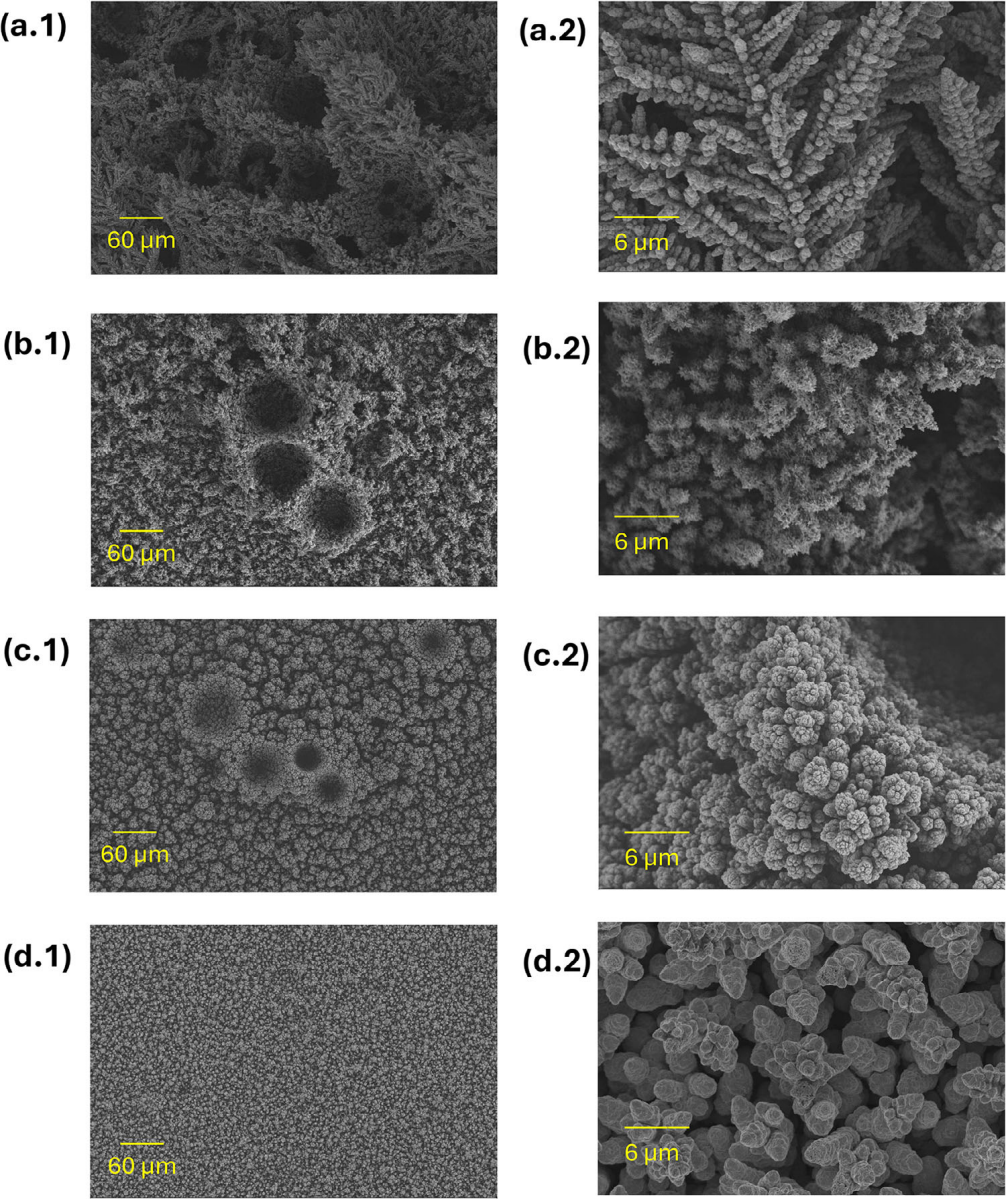
SEM images of Cu foam GDEs, showing the foam structure at 200x magnification (first column) and surface nano‐structures at 3000x magnification (second column) for the samples: ${\mathrm{DC}}_{30C\;{\mathrm{cm}}^{-2}}$ a), ${\mathrm{PC}}_{30C\;{\mathrm{cm}}^{-2}}$ b), ${\mathrm{PC}}_{30C\;{\mathrm{cm}}^{-2}}^{\mathrm{DAT}}$ c), and 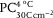 d).

**Figure** **13** shows SEM images of ${\mathrm{DC}}_{30C\;{\mathrm{cm}}^{-2}}$, ${\mathrm{PC}}_{30C\;{\mathrm{cm}}^{-2}}$, ${\mathrm{PC}}_{30C\;{\mathrm{cm}}^{-2}}^{\mathrm{DAT}}$, and 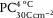 samples. Among them, only the ${\mathrm{DC}}_{30C\;{\mathrm{cm}}^{-2}}$ GDE displayed a uniform, well–connected foam network, whereas the ${\mathrm{PC}}_{30C\;{\mathrm{cm}}^{-2}}$ and ${\mathrm{PC}}_{30C\;{\mathrm{cm}}^{-2}}^{\mathrm{DAT}}$ samples showed isolated islands of foam structures. The addition of DAT produced noticeably smaller pores within these islands. Reducing the DHBT bath temperature suppressed foam formation and instead yielded Cu agglomerates for the sample 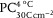.

At high magnification, the ${\mathrm{DC}}_{30C\;{\mathrm{cm}}^{-2}}$ sample exhibited surface nano–dendrites consistent with DC–deposited model foams. The ${\mathrm{PC}}_{30C\;{\mathrm{cm}}^{-2}}$ GDE featured cactus–like nano–structures, which differ from the nano‐cauliflower structures obtained from model PC samples. This contrast is plausibly attributable to the change in substrate from a planar Cu foil to porous Ag‐sputtered PTFE fabric. The ${\mathrm{PC}}_{30C\;{\mathrm{cm}}^{-2}}^{\mathrm{DAT}}$ sample exhibited surface nano‐cauliflowers similar to the model DAT‐mediated foams. The surface nano‐features of 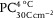 can be described as conical nano‐structures.

Gravimetric analysis and ECSA results of the Cu foam GDEs are provided in Figures S21 and S22 (Supporting Information). CO_2_RR performance was investigated in a flow cell setup, consisting of cathode and anode compartments separated by a Selemion anion exchange membrane, with 1 M KHCO_3_ circulated in both. The cathode featured separate electrolyte and gas chambers. The GDE catalytic layer faced the electrolyte, and its macro‐porous PP layer faced the CO_2_‐purged gas chamber. Outlet gas was switched via a three‐port valve to an online GC (every 15 min) or a bubble flow meter. Chronopotentiometric CO_2_RR was performed at 50, 100, 150, and 200 mA cm^−2^ for 1.5 h each. Flow cell details, chromatographic analysis, and CO_2_RR Faradaic efficiencies (FEs) calculations are provided in Section 5.6.

**Figure 14:**
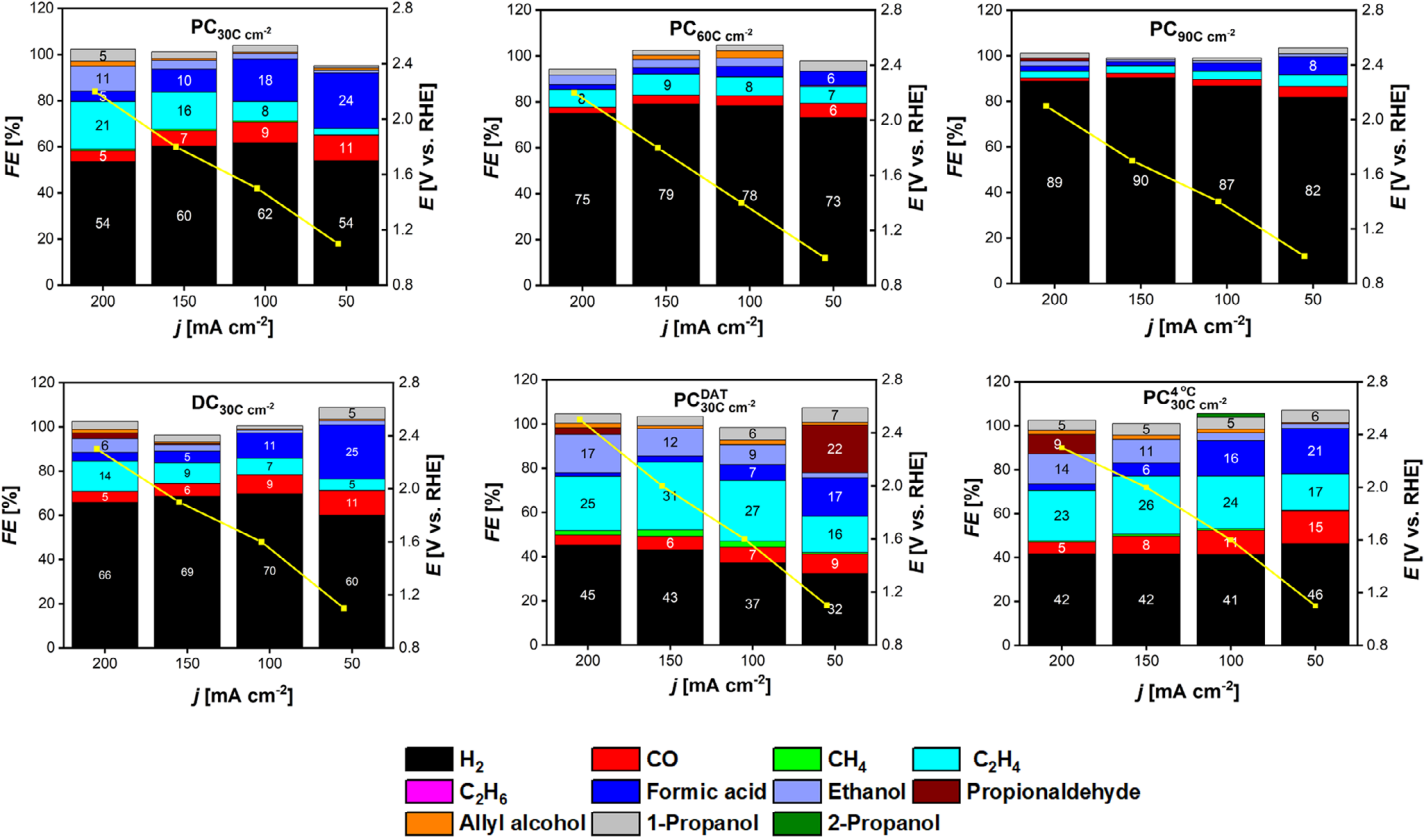
Faradic efficiencies of CO_2_RR products at 1.5 h of electrolysis for the Cu foam GDEs investigated in this work, showing the average potential recorded at each applied current density.

**Figure** **14** presents Faradaic efficiencies for CO_2_RR products measured at 1.5 h of electrolysis at the indicated applied current densities, together with the average potential recorded during each chronopotentiometric experiment. Representative examples of the gaseous product FEs over the 1.5 h period are shown in Figure S23 (Supporting Information), where FEs were found to increase gradually and start to approach a steady state after approximately 1.25 h for most samples.

The major detected products were H_2_, CO, formic acid, ethylene, ethanol, propionaldehyde, and 1‐propanol. Comparison of the GDEs fabricated at different DHBT charges (${\mathrm{PC}}_{30C\;{\mathrm{cm}}^{-2}}$, ${\mathrm{PC}}_{60C\;{\mathrm{cm}}^{-2}}$, and ${\mathrm{PC}}_{90C\;{\mathrm{cm}}^{-2}}$) indicates that lower DHBT charges are more beneficial for CO_2_RR activity. The performance of the ${\mathrm{PC}}_{60C\;{\mathrm{cm}}^{-2}}$ and ${\mathrm{PC}}_{90C\;{\mathrm{cm}}^{-2}}$ samples was dominated by parasitic H_2_ evolution (FEs: 73%–90%), whereas the ${\mathrm{PC}}_{30C\;{\mathrm{cm}}^{-2}}$ sample showed enhanced CO_2_RR with H_2_ FEs not exceeding 62%. The DC–derived GDE demonstrated inferior CO_2_RR activity relative to ${\mathrm{PC}}_{30C\;{\mathrm{cm}}^{-2}}$, with maximum H_2_ FEs of 70% and 62% observed at 100 mA cm^−2^ for the DC and PC counterparts, respectively. For the ${\mathrm{PC}}_{30C\;{\mathrm{cm}}^{-2}}$ sample, CO and formic acid FEs decreased gradually from 11% and 24% at 50 mA cm^−2^ to 5% and 5% at 200 mA cm^−2^. The ${\mathrm{DC}}_{30C\;{\mathrm{cm}}^{-2}}$ sample follows a similar trend (CO: 11% → 5%; formic acid: 25% → 4%) at the same current densities. In contrast, ethylene and ethanol exhibited negligible FEs at 50 mA cm^−2^, which increased with elevating the applied current densities to reach respective maxima of 21% and 11% for ${\mathrm{PC}}_{30C\;{\mathrm{cm}}^{-2}}$ and 14% and 6% for the DC counterpart at 200 mA cm^−2^. The samples ${\mathrm{PC}}_{30C\;{\mathrm{cm}}^{-2}}^{\mathrm{DAT}}$ and ${\mathrm{PC}}_{30C\;{\mathrm{cm}}^{-2}}^{4^{\circ }C}$ exhibited the best overall CO_2_RR performance, with corresponding suppressed parasitic H_2_ FEs of 37% and 41% at 100 mA cm^−2^, and enhanced C_2+_ performance, with ethylene FEs of 31% and 26% at 150 mA cm^−2^, and ethanol FEs of 17% and 14% at 200 mA cm^−2^. Propionaldehyde FEs of 22% at 50 mA cm^−2^ (for ${\mathrm{PC}}_{30C\;{\mathrm{cm}}^{-2}}^{\mathrm{DAT}}$) and 9% at 200 mA cm^−2^ (for ${\mathrm{PC}}_{30C\;{\mathrm{cm}}^{-2}}^{4^{\circ }C}$) were measured.

**Figure 15:**
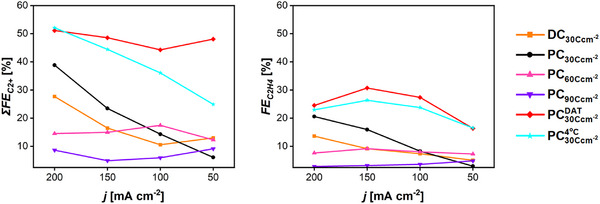
Total C_2+_ products (a) and ethylene (b) Faradic efficiencies measured at 1.5 h of electrolysis for the Cu foam GDEs.

**Figure** **15** presents the total C_2+_ products and ethylene selectivity for the six studied GDEs. The ${\mathrm{PC}}_{30C\;{\mathrm{cm}}^{-2}}^{\mathrm{DAT}}$, and ${\mathrm{PC}}_{30C\;{\mathrm{cm}}^{-2}}^{4^{o}C}$ samples showed the highest C_2+_ selectivity, with total C_2+_ FEs of 51%, and 52% at an applied current density of 200 mA cm^−2^ (≈2.4 V vs. RHE), corresponding to total partial current densities of 102, and 104 mA cm^−2^. These two samples also showed outperforming ethylene production at 150 mA cm^−2^ (≈2.0 V vs. RHE) with FE values of 31% and 26%, and ethylene partial current densities of 46.5, and 39 mA cm^−2^, respectively.

The Cu foam GDE findings suggest that meso– and nano–porosity, dominating the PC samples, are more beneficial for CO_2_RR C_2+_ selectivity with respect to macro‐porosity, dominating the DC samples. In the same context, previous studies showed that PC‐derived Ag foams displayed a better activity for CO_2_ reduction into CO than the DC‐derived counterparts electrodeposited at the same charge density.^[^ ^3^ ^]^ Accordingly, it can be suggested that highly macro‐porous and thicker foam architectures, e.g., for DC, PC_60C cm_^−2^, and PC_90C cm_^−2^, can suffer from internal mass–transport limitations that create concentration polarization between the external catalyst surface and the pore interior. Under high current densities, the effective CO_2_ concentration within deep or tortuous pores decreases, which shifts the reaction towards the more readily available water reduction in the pore regions depleted of CO_2_.^[^ ^48^ ^]^ This observation not only explains the outstanding performance of the PC GDE produced at 4^○^C, whose foam architecture is dominated only by nano‐ and meso‐pores, but also supports the view that CO_2_RR at GDEs can proceed via a two‐phase boundary, i.e., between the electrolyte (in which CO_2_ is dissolved) and the catalyst layer, rather than a pure three‐phase boundary involving gaseous CO_2_, liquid electrolyte, and catalytic layer.^[^ ^49^ ^]^

The enhanced C_2+_ performance of the ${\mathrm{PC}}_{30C\;{\mathrm{cm}}^{-2}}^{\mathrm{DAT}}$ sample can be attributed to chemical contributions. Amino‐based surface species, as indicated from XPS results of the DAT‐modified sample, have been reported to stabilize the *CO intermediate on the electrode surface and thereby facilitate the C‐C coupling, and enhance C_2+_ selectivity.^[^ ^50^ ^]^ Nevertheless, this hypothesis still requires direct confirmation for DAT and will be addressed in a future work. To evaluate the operational stability of the GDEs prepared via the presented protocol, a DAT‐modified GDE was synthesized and treated with 15 µL of the ionomer solution per each geometric 1 cm^−2^ of the GDE surface. The performance of this GDE was monitored during a 12 h chronopotentiometric experiment at a current density of 150 mA cm^−2^, with the CO_2_RR gaseous products analyzed every 15 min using the online GC, as presented in Figure S24 (Supporting Information). The measured potential remained nearly constant at approximately ‐1.5 V vs. RHE. Ethylene FE increased gradually and reached a plateau of about 39% after 2.5 h. This value remained essentially stable until the end of the experiment, decreasing only to 37% after 12 h. CO reached a FE plateau at about 9% after 1.25 h, which remained constant until the end of the experiment. Methane exhibited a maximum FE of 8.5% at 1.25 h, which declined gradually to approximately 4% after 12 h.

## 4 Conclusion

This study established a systematic correlation between various DHBT synthesis conditions and the resulting Cu foam morphologies. By tuning DHBT parameters, precise control over the hierarchical structure of the foam samples was achieved. Deposition current density and time had a significant impact: higher current densities with shorter durations produced more compact structures, while lower currents with longer times yielded more open structures. For instance, the (2Acm^−2^ 5s) sample showed respective average pore diameter and foam thickness of 9.4 ± 0.1 and 23.0 ± 1.5 µm, whereas the (1A cm^−2^ 30s) sample exhibited corresponding values of 36.7 ± 0.5 µm and 62.0 ± 4.7. This trend, which extended to surface nano‐dendrite size, was attributed to increased HER rates at higher current densities, which enhanced the number of bubble nucleation sites and reduced bubble sizes, leading to finer templating, lower local stirring, and slower Cu^2+^ diffusion dynamics.

Interrupting the cathodic signal in the PC and RC modes suppressed HER and altered Cu^2+^ diffusion hydrodynamics, resulting in foams with high mass densities, dominant meso‐ to nanoporosity, and surface cauliflower‐like nano‐structures. Foam structure in the PC samples was strongly influenced by the pulse‐to‐pause ratio. For instance, lower pulse‐to‐pause ratios produced isolated foam islands or no foam at all, with reduced thicknesses. Similarly, increasing the anodic charge in the RC mode further enhanced foam compactness by more effectively suppressing hydrogen evolution. AC‐DHBT at low and moderate frequencies (0.5–1000 Hz) yielded compact structures with smaller pores, moderate thickness, and finer nano‐dendrites.

Temperature and electrode alignment also influenced morphology: raising the bath temperature or using horizontal alignment increased bubble size and local stirring effects, producing larger pores and dendrites. Mechanical stirring of the DHBT bath yielded significantly enlarged nano‐dendrites, supporting the role of hydrodynamics in shaping surface features. Annealing expanded foam dimensions and refined nano‐structures via recrystallization.

A considerable Cu^2+^ concentration of 0.2 M was essential for driving Cu electrodeposition and HER concurrently and effectively achieving the characteristic honeycomb morphology, while reducing H^+^ concentration from 3 to 0.3 M confirmed that H_2_ bubbles can effectively originate from both proton and water reduction, as the honeycomb‐like structure was preserved. Additives like SDS, CTAB, and DAT (at low concentrations) promoted more compactness, surfactants by enhancing HER nucleation, and DAT by slowing Cu deposition through its complexation effect. At high DAT concentrations, with or without increasing the electrolyte pH to 0.5, the effect of DAT became more pronounced, leading to the loss of the interconnected foam structure.

As a proof‐of‐concept, a simple three‐step protocol for the fabrication of Cu foam GDEs was provided. The synthesized GDEs demonstrated promising CO_2_ reduction performance, reaching C_2+_ FEs of ≈50% at ‐1.1 V vs. RHE, C_2+_ partial current densities of up to 104 mA cm^−2^ at ‐2.5 V vs RHE, and stable operation over 12 h for the DAT‐modified GDE.

These findings demonstrate that Cu foams can be intelligently tailored for specific applications. For example, highly porous foams are promising for battery, solar energy conversion, and sensing applications, while compact foams are well‐suited for electrocatalytic applications, which will be explored more thoroughly in our future work. The herein reported comprehensive synthesis‐structure correlation for Cu foams can provide guidance in finding the right recipe and structure for a plethora of different functions required in different applications.

## 5 Experimental Section

### DHBT Synthesis of Model Cu Foams

### Standard DHBT Procedure

Cu sheets (0.1 mm thickness, Goodfellow GmbH, 99.999% purity) were cut into 1 cm × 2 cm foils. Each foil was polished using 0.7 µm diamond paste, ultrasonicated in ethanol and Milli‐Q water, and etched in nitric acid solution (10 vol%) (Fisher Chemical GmbH, 65%). Thereafter, the active area of the Cu foil for DHBT was defined as 1 cm × 1 cm using Kapton tape. Then, the Cu foils were weighed. All solutions were prepared in Milli‐Q water. Unless otherwise stated, the following standard DHBT procedure was used for Cu foams electrodeposition. The electrolyte consisted of a mixed solution of CuSO_4_ (0.2 M) (Sigma–Aldrich, 99.995% purity) and H_2_SO_4_ (1.5 M) (Carl Roth, 95%–98%), with a calculated pH of approximately ‐0.5. Electrodeposition was carried out in an open single‐compartment cell using a two‐electrode configuration (Figure S8, Supporting Information). The cell was filled with the DHBT electrolyte (20 mL). A polished Pt foil (1 cm × 2 cm) served as the counter electrode, and the prepared Cu substrate as the working electrode. Both electrodes were positioned vertically with a separation of about 0.3–0.5 cm. The DHBT process was conducted at room temperature from a quiescent solution using Gamry 3000 reference potentiostat. Immediately after deposition, the Cu foam sample was removed from the DHBT bath, rinsed with Milli‐Q water, dried under a nitrogen stream, and weighed. The mass of the deposited Cu foam was determined by subtracting the initial substrate mass from the final mass after DHBT (Figure S25, Supporting Information).

### DHBT Synthesis of the Current–Time Series

In this series, six Cu foam samples were synthesized using the standard DHBT procedure described above. The samples were electrodeposited at the following combinations of continuous cathodic DC densities and deposition times: 1 A cm^−2^ for 10 s, 1 A cm^−2^ for 20 s, 1 A cm^−2^ for 30 s, 2 A cm^−2^ for 5 s, 2 A cm^−2^ for 10 s, and 2 A cm^−2^ for 15 s.

### DHBT Synthesis of the Current Mode Series

Five Cu foam samples were electrodeposited using the PC mode with different pulse / pause time combinations: (1/10 ms), (5/10 ms), (20/10 ms), (20/40 ms), and (20/200 ms). A constant cathodic DC density of 1 A cm^−2^ was applied during the pulse phase. The total cathodic charge densityfor each sample was fixed at 20 C cm^−2^, controlled by adjusting the number of pulse–pause cycles. The corresponding number of cycles applied for the five samples was 20000, 4000, 1000, 1000, and 1000, respectively.

For the RC DHBT, the cathodic and anodic pulse durations were fixed at 20 ms and 10 ms, respectively, with a total number of cathodic‐anodic pulse pairs of 1000 applied per sample. Three different cathodic / anodic pulse current density pairs were used: (1.05/0.1 A cm^−2^), (1.15 A/0.3 A cm^−2^), and (1.3 A/ 0.6 A cm^−2^). The total charge density passed in each case was maintained at 20 C cm^−2^.

For the AC DHBT, a cathodic DC offset of 1 A cm^−2^ was applied, and the deposition time was fixed at 20 s. Five different combinations of AC amplitudes and frequencies were tested: (1.2/ 0.5 Hz), (1.5/ 0.5 Hz), (2/0.5 Hz), (1.5/1000 Hz), and (1.5/ 5000 Hz). The total charge density was kept constant at 20 C cm^−2^ for all the AC samples.

PC, RC, and AC samples were referenced to a sample electrodeposited at a continuous cathodic DC density of 1 A cm^−2^ for 20 s with a similar charge density of 20 C cm^−2^.

### DHBT Synthesis of the Physical Effect Series

Several modifications were made to the standard DHBT procedure, with all samples electrodeposited at a constant continuous cathodic DC density of 1 A cm^−2^ for 20 s. The changes made in this series were made individually as follows:

(a) A sample was deposited under electrolyte stirring at 1350 rpm using a magnetic stirrer.
(b) Two samples were prepared at 5 and 60 °C, using ice and water baths, respectively, to assess the influence of thermal conditions.
(c) A custom‐designed cell was employed to achieve horizontal alignment of the working and counter electrodes, with their electrical connections made laterally via aluminum tape strips surrounded with insulating Kapton tape, leaving exposed ends for electric connection to the potentiostat. These strips were threaded through side apertures in the cell wall and bent upward, while a hydrophobic parafilm layer was tightly secured over the connectors using screws to prevent leakage, none of which was observed during operation. Figures S6 and S7 (Supporting Information) illustrate the horizontal DHBT cell design and the electrode connectors.
(d) Post‐DHBT annealing of the Cu foam samples was performed in air under two different conditions: 300 °C for 15 min and 150 °C for 30 min. After cooling to room temperature, the samples were re‐weighed to assess mass changes due to oxidation.

A reference sample for this series was prepared at a continuous cathodic DC density of 1 A cm^−2^ for 20 s with no modifications to the standard DHBT procedure.

### DHBT Synthesis of the Chemical Effect Series

In this series, modifications were made to the electrolyte composition described in the standard DHBT procedure. All samples were electrodeposited at a continuous cathodic DC density of 1 A cm^−2^ for 20 s. These were referenced to a Cu foam sample deposited under identical current density and time using the standard DHBT electrolyte. Each of the following electrolyte changes was introduced individually:

(a) Changes to CuSO_4_ concentration:
  (i) CuSO_4_ (0.05 M) and H_2_SO_4_ (1.5 M) (pH ≈ ‐0.5).
  (ii) CuSO_4_ (0.1 M) and H_2_SO_4_ (1.5 M) (pH ≈ ‐0.5).
(b) A change to H_2_SO_4_ concentration:
  (i) CuSO_4_ (0.2 M) and H_2_SO_4_ (0.15 M) (pH ≈ 0.5).
(c) Adding surfactants:
  (i) CuSO_4_ (0.2 M), H_2_SO_4_ (1.5 M), and SDS (0.25 mM) (pH ≈ −0.5).
  (ii) CuSO_4_ (0.2 M), H_2_SO_4_ (1.5 M), and CTAB (0.25 mM) (pH ≈ −0.5).
(d) Adding a complexing agent without and with pH increasing:
  (i) CuSO_4_ (0.2 M), H_2_SO_4_ (1.5 M), and DAT (4 mM) (pH ≈ −0.5).
  (ii) CuSO_4_ (0.2 M), H_2_SO_4_ (1.5 M), and DAT (1.5 mM) (pH ≈ −0.5).
  (iii) CuSO_4_ (0.2 M), H_2_SO_4_ (0.15 M), and DAT (4 mM) (pH ≈ 0.5).

### X‐Ray Analysis of the Cu Foam Samples

XRD patterns of Cu foams were collected on a Rigaku SmartLab diffractometer equipped with a rotating Cu anode of a wavelength of 1.54 Å at 45 kV / 200 mA and a HyPix‐3000 2D detector. Data acquisition was conducted in a range of 10 to 100° 2θ using a step size of 0.02° with a fixed incident angle of 0.35° and a scan rate of 8° min^−1^. Data were analyzed using OriginPro software, and the Williamson–Hall method was used to calculate the particle size and micro‐strain.

Elemental analysis was performed with a Bruker XFlash 6–100 energy‐dispersive X‐ray spectroscopy detector at an acceleration voltage of 5 keV.

XXPS was performed at room temperature under ultrahigh vacuum (≈10^−7^ Pa) using a SPECS PHOIBOS 100 instrument with an Al Kα X‐ray source (1486.74 eV). High‐resolution spectra were recorded with a step size of 0.1 eV, and survey spectra with a step size of 0.80 eV, at a dwell time of 0.05 s per step. Prior to XPS measurements, Cu foam samples were additionally washed to remove unbound additives or any residual H_2_SO_4_ that might exist within the pores. Accordingly, samples were rinsed in 0.1 M KHCO_3_ for at least 5 min to neutralize any trapped acid, rinsed in Milli–Q water for at least 5 min, and dried under a N_2_ stream. Survey and high‐resolution spectra were normalized, baseline‐corrected, and subjected to Shirley background subtraction using Fityk software.

### Scanning Electron Microscopy Imaging

SEM images were collected using the in‐lens and secondary electron detectors of a scanning electron microscope (Zeiss Ultra plus, Carl Zeiss, Germany), equipped with a high‐resolution field emission gun. Low‐ (200x) and high‐magnification (3000x) SEM images were collected at an acceleration voltage of 3.00 KV.

### Electrochemical Measurements in H‐Cell Configuration

ECSA measurements were performed at room temperature in a custom gas‐tight two‐compartment glass H‐cell assembled with three electrodes (Figure S26, Supporting Information). The WE and reference electrode (RE) shared the cathode compartment, while the CE was placed in the opposite compartment. The two compartments were separated by a Selemion anion exchange membrane (AEM) (AGC Chemicals Europe, England). A Cu foam of 1 cm^2^ geometric area served as the WE, a Pt mesh (mesh size 80, 2.5 cm × 3.5 cm, ALS, Japan) as the CE, and a commercial Ag/AgCl electrode (ALS, Japan) as the RE. All reported potentials were iR‐corrected and referenced to RHE. The electrolyte was 0.1 M KHCO_3_ (>99.7%, Carl Roth, Germany). Electrochemical control was provided by a Gamry Reference 3000 potentiostat (Gamry Instruments, USA). To convert unstable Cu oxide species to metallic Cu, the samples were conditioned by CV: the potential was cycled between ‐0.05 and ‐1.05 V vs. RHE at 100 mV s^−1^ for up to 20 cycles until stable current responses were obtained. Then, the ECSA of the Cu foam samples was measured using two independent electrochemical methods: CV and EIS. In the CV‐based method, cyclic voltammograms were recorded between 0.15 and 0.35 V versus RHE, a window free of Faradaic currents, at scan rates from 10 to 100 mV s^−1^. The sum of the anodic and cathodic charging currents (Δ*i*) was plotted versus scan rate (ν). The double‐layer capacitance (*C*_*DL*_) of the Cu foam was obtained as half the slope of that plot according to Equation (9). The ECSA was then calculated using Equation (2). *C*_*Ref*_ was determined from a polished and cleaned 1 cm^2^ Cu foil by extracting the double‐layer capacitance from the plot of the summed charging and discharging currents, and using this value as the reference capacitance

(9) $$C_{\mathrm{DL}}=\frac{1}{2}.\frac{d(\Delta i)}{d(\nu )}$$

**Figure 16:**
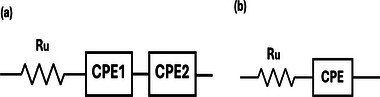
Equivalent circuit models used to fit the EIS data of the Cu foam (a) and Cu foil (b).

Electrochemical impedance spectra were recorded at a non‐Faradaic potential of 0.25 V versus RHE with a 5 mV perturbation across a frequency range of 10 kHz to 0.5 Hz. The spectra were fitted in Aftermath software to an equivalent circuit consisting of the uncompensated solution resistance (Ru) in series with two constant phase elements (CPEs), as illustrated in **Figure** **16**a. The high‐frequency CPE was attributed to double‐layer charging of the readily accessible outer surface and larger pores, while the low‐frequency CPE represented full electrode charging.^[^ ^51^ ^]^ The double‐layer capacitance (*C*_*DL*_) of the Cu foam was taken from the low‐frequency CPE and inserted into Equation (2) to compute the ECSA. *C*_*Ref*_ for the EIS‐based procedure was determined from impedance spectra acquired under identical conditions on a polished and cleaned Cu foil of 1 cm^2^ geometric area. These spectra were modeled with a simplified equivalent circuit consisting of Ru in series with a single CPE (Figure 16b), and the fitted double‐layer capacitance was taken as *C*_*Ref*_.^[^ ^52^ ^]^ The measured *C*_*ref*_ values were 0.0417 ± 0.0050 mF cm^−2^ (for CV‐based ECSA) and 0.0473 ± 0.0100 mF cm^−2^ (for EIS‐based ECSA) with the error bars indicating the standard deviation of three independent measurements.

### DHBT Synthesis of Cu Foam Gas Diffusion Electrodes

**Figure 17:**
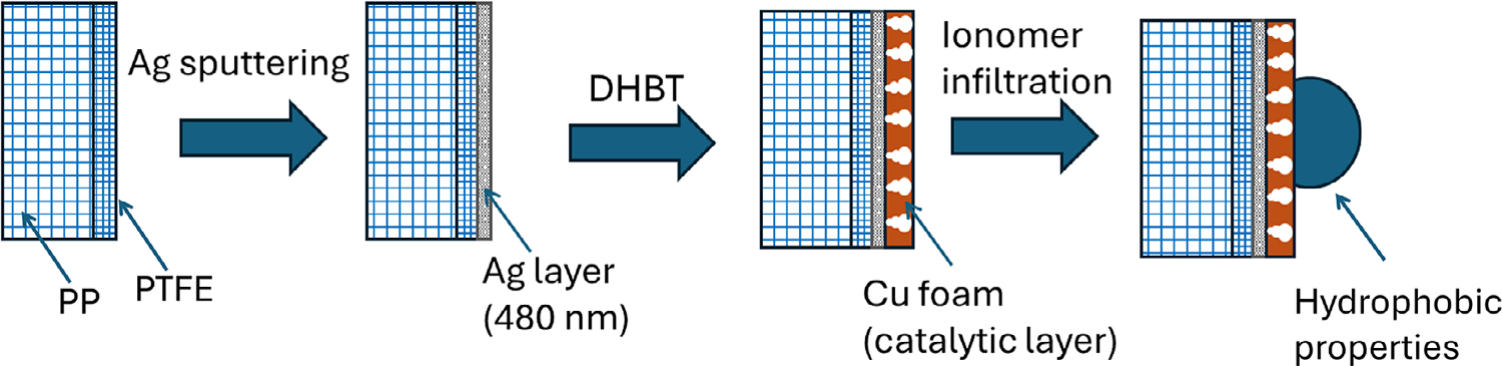
Schematic representation of the DHBT synthesis protocol employed to fabricate the Cu foam GDEs.

PTFE/PP bilayer fabric sheets (20 cm x 25 cm, 0.2 micron, Sterlitech, USA) were cut into 3 cm × 4 cm pieces and sputter‐coated on the PTFE side with a 480 nm Ag layer thickness at a target current of 25 mA using a VAC COAT Desk sputter‐coater to impart electrical conductivity. The coated substrates were weighed and then mounted in a custom 3D‐printed ABS holder, comprising a front window with an integrated top electrical contact and a solid back cover. The Ag‐coated fabric was clamped between the two parts using four corner insulating screws and was oriented so that the sputtered PTFE side faced the window, exposing an active area of 3 × 2.5 cm for DHBT, while the PP side rested against the back cover. This assembly served as the WE. The CE consisted of a sacrificial Cu mesh (50 mesh size, 0.23 mm wire diameter, Alfa Aesar) secured between two ABS windows with four insulating corner screws. The standard DHBT synthesis of the Cu foam GDEs was performed in a two‐electrode setup, in which the Ag‐sputtered PTFE substrate and Cu mesh, both placed in their holders, served as the WE and CE, respectively. **Figure** **17** provides a schematic representation of the Cu foam GDEs synthesis protocol. Also, the WE and CE holders, as well as the GDE DHBT setup, are presented in Figure S27 (Supporting Information).

Unless otherwise mentioned, the DHBT was carried out at room temperature in a quiescent electrolyte of 0.2 M CuSO_4_ and 1.5 M H_2_SO_4_ with a volume of 100 mL. A Gamry 3000 reference potentiostat equipped with a Gamry reference 30k booster was employed for DHBT. During DHBT, the PC mode was employed with a cathodic pulse current density of 0.667 A cm^−2^, pulse duration of 20 ms, and pause duration of 40 ms, repeated over 2250 cycles to achieve a total electrodeposition charge density of 30 C cm^−2^.

In addition to this standard procedure, the following modifications were individually implemented:

(a) To compare the PC and DC modes at the GDE level, a DC sample was electrodeposited at a continuous cathodic DC density of 0.667 A cm^−2^ for 45 s.
(b) To investigate the effect of electrodeposition charge density on the GDE performance, Cu foam GDEs were additionally fabricated at cathodic charge densities of 60 and 90 C cm^−2^, using 4500 and 6750 pulse–pause cycles, respectively, alongside the reference sample deposited at 30 C cm^−2^.
(c) DAT at a concentration of 4 mM was added to the DHBT bath.
(d) A sample was electrodeposited at 4 °C using an ice bath.

Following DHBT, the obtained Cu foam sample was immediately removed from the bath and rinsed sequentially with Milli‐Q water, 0.1 M KHCO_3_ solution to neutralize residual acid in the foam pores, and a final Milli‐Q water rinse. The sample was left to dry completely and then weighed.

For the hydrophobic treatment of the Cu‐foam GDE, a perfluorosulfonic acid ionomer solution (800 EW, 3 M) was prepared by dissolving 15 mg of the ionomer in 10 mL of a 95:5 (v/v) 1‐propanol/water mixture and stirring the mixture for 24 h using a magnetic stirrer to ensure complete dissolution. Unless otherwise stated, each geometric 1 cm^2^ of Cu‐foam GDE surface was then drop‐cast with 5 µL of the ionomer solution, and then was left to dry for a period of 24 h before any measurements.

### Electrochemical Characterization of Cu Foam GDEs in Flow Cell Setup

**Figure 18:**
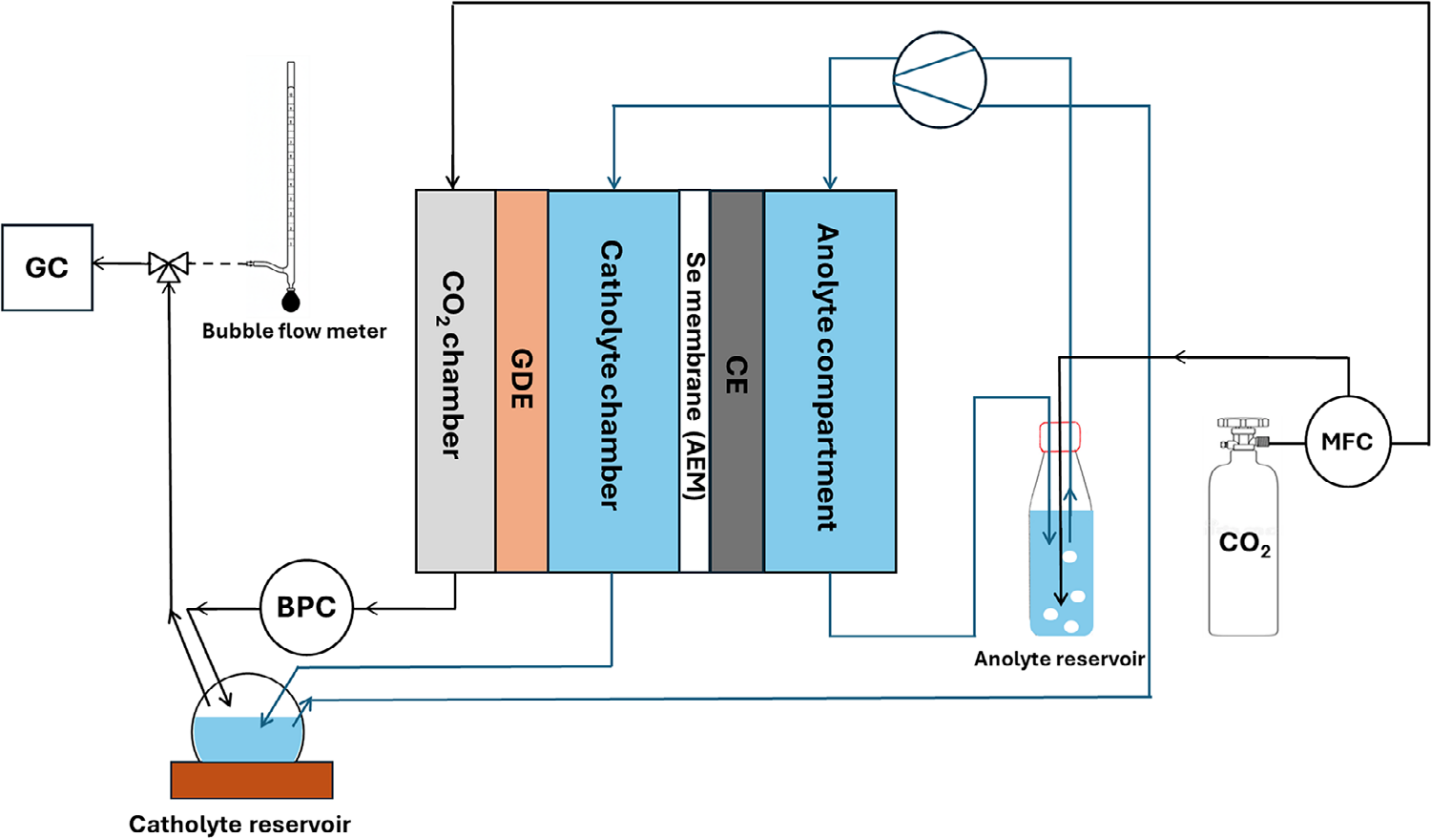
Flow cell configuration used for CO_2_RR measurements of the Cu foam GDEs.

Electrochemical characterization of the Cu‐foam GDEs was performed using the flow cell configuration illustrated in **Figure** **18** and Figure S28 (Supporting Information). This setup consists of distinct cathode and anode compartments separated by a Selemion anion exchange membrane (AGC Chemicals Europe, England), with 1 M KHCO_3_ serving as the electrolyte. A calibrated mass flow controller (MFC) (Bronkhorst, Netherlands) regulated the CO_2_ flow rate, while a dual‐channel peristaltic pump (Shenchen Pump YZ1515x) ensured independent circulation of catholyte and anolyte between their respective reservoirs and compartments. The cathode compartment comprised separate CO_2_ and electrolyte chambers, between which the Cu‐foam GDE was placed. The catalytic layer of the GDE faced the electrolyte chamber, whereas the PP macro‐porous layer was oriented toward the CO_2_ chamber. The CO_2_ chamber and anolyte reservoir were continuously supplied with CO_2_ using the MFC. A backpressure of 30 mbar was applied to the CO_2_ chamber outlet using a backpressure controller (BPC) (Bronkhorst, Netherlands). The BPC outlet gas stream was introduced into the headspace of the catholyte reservoir, from which the stream could be selectively diverted via a three‐port valve to the online gas chromatograph (GC) inlet or a bubble flow meter, ensuring reliable flow rate tracking between GC injection events. In all CO_2_ experiments, a Pt mesh (mesh size 80, 2.5 cm × 3.5 cm, ALS, Japan) was employed as the CE, a HydroFlex Reversible Hydrogen Electrode (Gaskatel GmbH, Germany) as the RE, and the Cu‐foam GDE as the WE. Unless otherwise specified, both cathode and anode compartments were supplied with CO_2_ at a flow rate of 20 mL min^−1^, and with the electrolyte at a flow rate of 50 mL min^−1^. Electrochemical measurements started with CVs in the potential range of ‐0.05 to ‐1.05 V versus RHE at a scan rate of 100 mV s^−1^, repeated for up to 20 cycles until stable and reproducible currents were achieved, thereby reducing the surface unstable oxides. For ECSA determination, the CO_2_ flow rate was reduced to 5 mL min^−1^, and the pump was temporarily halted to minimize mechanical disturbances affecting double‐layer charging and discharging. Then, ECSA CVs were recorded within a potential window of 0.15–0.35 V versus RHE at scan rates ranging from 10 to 100 mV s^−1^. Thereafter, Equations (2) and (9) were used to calculate the ECSA of the GDE.

Following ECSA measurements, the CO_2_ flow rate was restored to 20 mL min^−1^, and the electrolyte circulation resumed at 50 mL min^−1^. CO_2_ electrolysis was then conducted via chronopotentiometry at cathodic current densities of 50, 100, 150, and 200 mA cm^−2^, each for 1.5 h.

During CO_2_RR electrolysis, an online GC (Shimadzu GC‐2014) was used for the detection and quantification of CO_2_RR gaseous products every 15 minutes using serial HayeSep Q and HayeSep R columns and high‐purity argon (Grade Ar 5.0) as the carrier gas. The GC was equipped with a thermal conductivity detector (TCD) for H_2_ detection and a methanizer plus flame ionization detector (FID) for CO_2_, CO, and hydrocarbons detection. The GC was calibrated for the expected CO_2_RR gas products using two standard gas mixtures (Riessner‐Gase, Germany) covering the following mol% ranges: H_2_ (0.999–14.964 mol%), CO (0.114–2.003 mol%), CH_4_ (0.105–1.997 mol%), C_2_H_4_ (0.105–2.017 mol%), and C_2_H_6_ (0.00511–0.0513 mol%). After each electrolysis chronopotentiometric measurement, a catholyte sample was collected and analyzed for liquid products by a high‐performance liquid chromatograph (HPLC) (LC2010, Shimadzu) and a liquid gas chromatograph (LGC, Nexis GC‐2030, Shimadzu).

The HPLC and LGC were calibrated for the expected analytes. For HPLC calibration, mixed standard solutions of formic (⩾ 99.7%, Sigma–Aldrich) and acetic acid (⩾ 99.99%, Sigma–Aldrich), both over a concentration range of 0.04–2.00 mM, were used. For LGC calibration, mixed standard solutions of methanol (⩾99.9%, Merck) 0.13–5.08 mM; ethanol (⩾99.9%, Merck) 0.07–2.45 mM; acetone (⩾99.5%, Sigma–Aldrich) 0.06–2.49 mM; 1‐propanol (⩾99.5%, Merck) 0.04–1.70 mM; 2‐propanol (⩾99.9%, Sigma–Aldrich) 0.04–1.69 mM; allyl alcohol (⩾99%, Sigma–Aldrich) 0.05–1.87 mM; propionaldehyde (⩾98%, Sigma–Aldrich) 0.04–1.53 mM; and acetaldehyde (⩾99.5%, Sigma–Aldrich) 0.03–1.04 mM were used. All calibration curves were linear across their ranges. Figure S29 (Supporting Information) depicts examples of the chromatograms of standard mixtures injected into the online GC, LGC, and HPLC.

Faradaic efficiencies for each CO_2_RR product (*FE*_*i*_) were obtained by dividing the product partial current density (*j*_*i*_) by the total current density (*I*_*t*_) according to Equation 10. Partial current densities for gaseous and liquid products were calculated using Equations (11) and (12), respectively, with the following variables: gas product mole fraction (*x*_*i*_), number of electrons transferred (*z*_*i*_), molar flow rate (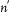), liquid product concentration (*c*_*i*_), catholyte volume (*V*_*c*_), and electrolysis time (*t*). The molar flow rate 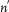 was calculated from the ideal gas law using pressure (*P*), volumetric gas flow rate (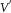), which was measured via the bubble flow meter, the general gas constant (*R*), and absolute temperature (*T*).

(10) $$FE_{i}=\frac{j_{i}}{I_{t}}\cdot 100\%$$

(11) $$j_{i}=x_{i}.z_{i}.F.n^{'}$$

(12) $$j_{i}=\frac{c_{i}.z_{i}.F.V_{c}}{t}$$

(13) $$n^{'}=\frac{P.V^{'}}{R.T}$$

### Contact Angle Measurements

To evaluate the hydrophobic properties of the GDE, the contact angle was measured using a DataPhysics optical contact angle device (Germany). A 10 µL droplet of Milli‐Q water was dispensed onto the dry Cu foam GDE. The recorded images were subsequently analyzed with the SCA 20 software to extract the contact angle values.

### Statistical Information

Gravimetric results (deposited Cu mass and deposition Faradaic efficiency) of the model Cu foam samples are reported as the mean of 15–20 independent replicates, with error bars indicating the standard deviation.

ECSA values of the model Cu foams are reported as the mean of 3–6 independent measurements, with error bars indicating the standard deviation.

For each DHBT condition, 2–4 independent SEM measurements were performed. Surface pores in the SEM images were annotated using Apeer.com online tools and processed by a Python script based on scikit‐image library to exclude edge‐touching pores and compute individual pore area and equivalent diameter. Typically, several hundred to a few thousand equivalent pore diameter values were extracted from the annotated images per DHBT condition, and used to plot the pore diameter distributions. The number of equivalent diameter values contributing to each distribution is indicated on the figure. The mean pore diameter is reported as the distribution expected value, and the associated error bars denote the standard error. The workflow of the Cu foams pore diameter analysis is shown in Figure S30 (Supporting Information).

Foam thickness is reported as the mean of six measurements taken at different positions on each sample, and the associated error is given as the standard deviation.

Two outliers were identified in the correlation between foam thickness and mean pore diameter; the analysis was performed both with and without these outliers, and the treatment of outliers was clearly stated in the text and indicated in Figure S12 (Supporting Information).

The Cu foam GDE results represent successful initial attempts to convert model Cu foams into a GDE architecture.

## Conflict of Interest

The authors declare no conflict of interest.

## Supporting information

Supporting Information

## Data Availability

The data that support the findings of this study are available from the corresponding author upon reasonable request.

## References

[1] S. M. Jung , D. J. Preston , H. Y. Jung , Z. Deng , E. N. Wang , J. Kong , Adv. Mater. 2016, 28, 1413. 26635235 10.1002/adma.201504774

[2] C. Du , P. Li , Z. Zhuang , Z. Fang , S. He , L. Feng , W. Chen , Coord. Chem. Rev. 2022, 466, 214604.

[3] H. Hoffmann , M. Kutter , J. Osiewacz , M. Paulisch‐Rinke , S. Lechner , B. Ellendorff , A. Hilgert , I. Manke , T. Turek , C. Roth , EES Catalysis 2024, 2, 286.

[4] A. D. McNaught , A. Wilkinson , IUPAC Compendium of Chemical Terminology, 2nd edition, Blackwell Scientific Publications, Oxford, 1997.

[5] S. Vesztergom , A. Dutta , M. Rahaman , K. Kiran , I. Zelocualtecatl Montiel , P. Broekmann , ChemCatChem 2021, 13, 1039. 10.1021/acsami.1c07829 34288647

[6] N. C. Bigall , A.‐K. Herrmann , M. Vogel , M. Rose , P. Simon , W. Carrillo‑Cabrera , D. Dorfs , S. Kaskel , N. Gaponik , A. Eychmüller , Angew. Chem., Int. Ed. 2009, 48, 9731. 10.1002/anie.200902543 19918827

[7] B. Tappan , M. Huynh , M. Hiskey , D. E. Chavez , E. P. Luther , J. T. Mang , S. F. Son , J. Am. Chem. Soc. 2006, 128, 6589. 16704258 10.1021/ja056550k

[8] D. Walsh , L. Arcelli , T. Ikoma , J. Tanaka , S. Mann , Nat. Mater. 2003, 2, 386. 12764358 10.1038/nmat903

[9] J. Erlebacher , M. J. Aziz , A. Karma , N. Dimitrov , K. Sieradzki , Nature 2001, 410, 450. 11260708 10.1038/35068529

[10] B. J. Plowman , L. A. Jones , S. K. Bhargava , Chem. Commun. 2015, 51, 4331. 10.1039/c4cc06638c 25649756

[11] T. Kottakkat , K. Klingan , S. Jiang , Z. P. Jovanov , V. H. Davies , G. A. M. El‑Nagar , H. Dau , C. Roth , ACS Appl. Mater. Interfaces 2019, 11, 14734. 30933468 10.1021/acsami.8b22071

[12] K. Klingan , T. Kottakkat , Z. P. Jovanov , S. Jiang , C. Pasquini , F. Scholten , P. Kubella , A. Bergmann , B. R. Cuenya , C. Roth , H. Dau , ChemSusChem 2018, 11, 3449. 30160827 10.1002/cssc.201801582

[13] M. Das , A. Biswas , T. Purkait , T. Boruah , S. Bhardwaj , S. K. Das , R. S. Dey , J. Mater. Chem. A 2022, 10, 13589.

[14] N. D. Nikolić , K. I. Popov , L. J. Pavlović , M. Pavlović , Surf. Coat. Technol. 2006, 201, 560.

[15] D. Cardoso , S. Eugénio , T. Silva , D. Santos , C. Sequeira , M. Montemor , RSC Adv. 2015, 5, 43456.

[16] W. Zhu , N. Hu , Q. Wei , L. Zhang , H. Li , J. Luo , C.‐T. Lin , L. Ma , K. Zhou , Z. Yu , Mater. Des. 2019, 172, 107709.

[17] G. Yang , J. Chen , P. Xiao , P. O. Agboola , I. Shakir , Y. Xu , J. Mater. Chem. A 2018, 6, 9899.

[18] D. Wang , J. Li , Y. Zhao , H. Xu , J. Zhao , Electrochim. Acta 2019, 316, 8.

[19] W. Fu , Y. Du , J. Jing , C. Fu , M. Zhou , Appl. Catal., B 2023, 324, 122201.

[20] Q. Hu , J. Qin , X.‐F. Wang , G.‐Y. Ran , Q. Wang , G.‐X. Liu , J.‐P. Ma , J.‐Y. Ge , H.‐Y. Wang , Front. Chem. 2021, 9, 786970. 34912785 10.3389/fchem.2021.786970 PMC8666423

[21] A. Abas , H. Sheng , Y. Ma , X. Zhang , Y. Wei , Q. Su , W. Lan , E. Xie , J. Mater. Sci.: Mater. Electron. 2019, 30, 10953.

[22] Y. Zheng , Y. Su , C. Pang , L. Yang , C. Song , N. Ji , D. Ma , X. Lu , R. Han , Q. Liu , Environ. Sci. Technol. 2021, 56, 1905. 34856794 10.1021/acs.est.1c05855

[23] G. Li , Y. Wang , H. Guo , Z. Liu , P. Chen , X. Zheng , J. Sun , H. Chen , J. Zheng , X. Li , J. Mater. Chem. A 2020, 8, 16920.

[24] Q. Chen , X. An , Q. Liu , X. Wu , L. Xie , J. Zhang , W. Yao , M. S. Hamdy , Q. Kong , X. Sun , Chem. Commun. 2022, 58, 517. 10.1039/d1cc06215h 34908040

[25] S. Min , X. Yang , A.‐Y. Lu , H. Li , Z. Idriss , M. N. Hedhili , K.‐W. Huang , L.‐J. Li , Nano Energy 2016, 27, 121.

[26] J. Zhou , F. Pan , Q. Yao , Y. Zhu , H. Ma , J. Niu , J. Xie , Appl. Catal., B 2022, 317, 121811.

[27] F. Chen , C. Chen , Q. Hu , B. Xiang , T. Song , X. Zou , W. Li , B. Xiong , M. Deng , Chem. Eng. J. 2020, 401, 126145.

[28] A. Dutta , M. Rahaman , N. C. Luedi , M. Mohos , P. Broekmann , ACS Catal. 2016, 6, 3804.

[29] Y. Wang , A. Dutta , A. Iarchuk , C. Sun , S. Vesztergom , P. Broekmann , ACS Catal. 2023, 13, 8169. 37342835 10.1021/acscatal.3c00716 PMC10278070

[30] E. Laborda , J. González , A. Molina , J. Solid State Electrochem. 2024, 28, 1259.

[31] H. Hoffmann , M. C. Paulisch‐Rinke , M. Gernhard , Y. Jännsch , J. Timm , C. Brandmeir , S. Lechner , R. Marschall , R. Moos , I. Manke , C. Roth , Commun. Chem. 2023, 6, 50. 36928610 10.1038/s42004-023-00847-z PMC10020469

[32] N. D. Nikolić , G. Branković , Electrochem. Commun. 2010, 12, 740.

[33] N. D. Nikolić , G. Branković , V. M. Maksimović , J. Electroanal. Chem. 2011, 661, 309.

[34] N. D. Nikolić , G. Branković , Mater. Lett. 2012, 70, 11.

[35] M. Maksimović , D. Totovski , A. Ivić , Surface Technol. 1983, 18, 233.

[36] T. Shao , C. Zhang , Pulsed Discharge Plasmas: Characteristics and Applications, Springer Nature, Cham, Switzerland 2023, ISBN 978‐981‐99‐1140‐0.

[37] F. Fasmin , R. Srinivasan , J. Electrochem. Soc. 2017, 164, H443.

[38] M. Fouad , G. Sedahmed , Electrochim. Acta 1973, 18, 55.

[39] S. Zhan , R. Yuan , X. Wang , W. Zhang , K. Yu , B. Li , Z. Wang , J. Wang , Phys. Fluids 2023, 35, 3.

[40] J. Niu , X. Liu , K. Xia , L. Xu , Y. Xu , X. Fang , W. Lu , Int. J. Electrochem. Sci. 2015, 10, 7331.

[41] S. Kang , Y. Obeng , M. Decker , M. Oh , S. M. Merchant , S. K. Karthikeyan , C. S. Seet , A. S. Oates , J. Electron. Mater. 2001, 30, 1506.

[42] D. Giziński , A. Brudzisz , J. S. Santos , F. Trivinho‐Strixino , W. J. Stępniowski , T. Czujko , Catalysts 2020, 10, 1338.

[43] Y. Li , W.‐Z. Jia , Y.‐Y. Song , X.‐H. Xia , Chem. Mater. 2007, 19, 5758.

[44] T. T. Hoang , S. Ma , J. I. Gold , P. J. Kenis , A. A. Gewirth , ACS Catal. 2017, 7, 3313.

[45] M. S. Hossain , S. Ahmed , Result. Mater. 2023, 20, 100492.

[46] S. I. Yusuf , S. J. Mohammad , M. H. Ali , J. Theoret. Appl. Phys. 2024, 18, 17.

[47] K. Maniammal , G. Madhu , V. Biju , Phys. E: Low‐Dimensio. Syst. Nanostructures 2017, 85, 214.

[48] S. Nitopi , E. Bertheussen , S. B. Scott , X. Liu , A. K. Engstfeld , S. Horch , B. Seger , I. E. Stephens , K. Chan , C. Hahn , J. K. Nørskov , T. F. Jaramillo , I. Chorkendorff , Chem. Rev. 2019, 119, 7610. 31117420 10.1021/acs.chemrev.8b00705

[49] D. Bohra , J. H. Chaudhry , T. Burdyny , E. A. Pidko , W. A. Smith , ChemRxiv 2020.

[50] X. Chen , J. Chen , N. M. Alghoraibi , D. A. Henckel , R. Zhang , U. O. Nwabara , K. E. Madsen , P. J. Kenis , S. C. Zimmerman , A. A. Gewirth , Nat. Catal. 2021, 4, 20.

[51] B. Serapinienė , L. Gudavičiūtė , S. Tutlienė , A. Grigucevičienė , A. Selskis , J. Juodkazytė , R. Ramanauskas , Coatings 2023, 13, 1335.

[52] J. Huang , Y. Gao , J. Luo , S. Wang , C. Li , S. Chen , J. Zhang , J. Electrochem. Soc. 2020, 167, 166503.

