# Supplementary material for: Engineering Hierarchically Nano‐Structured Cu Foams: Dynamic Hydrogen Bubble Templated Binder‐Free Freestanding Electrodes for Energy Applications
Source: Small. 2025 Dec 26;22(8):e09389. doi: 10.1002/smll.202509389 (PMC12877999; doi:10.1002/smll.202509389)
Supplement: Supplementary file 1 — Supporting Information [file SMLL-22-e09389-s001.docx]

Supporting Information

Engineering Hierarchically Nano-structured Cu Foams: Dynamic Hydrogen Bubble Templated Binder-free Freestanding Electrodes for Energy Applications

Mina Attia*, Chen Zhao, Miriam Lindner, Philipp Hawe & Christina Roth


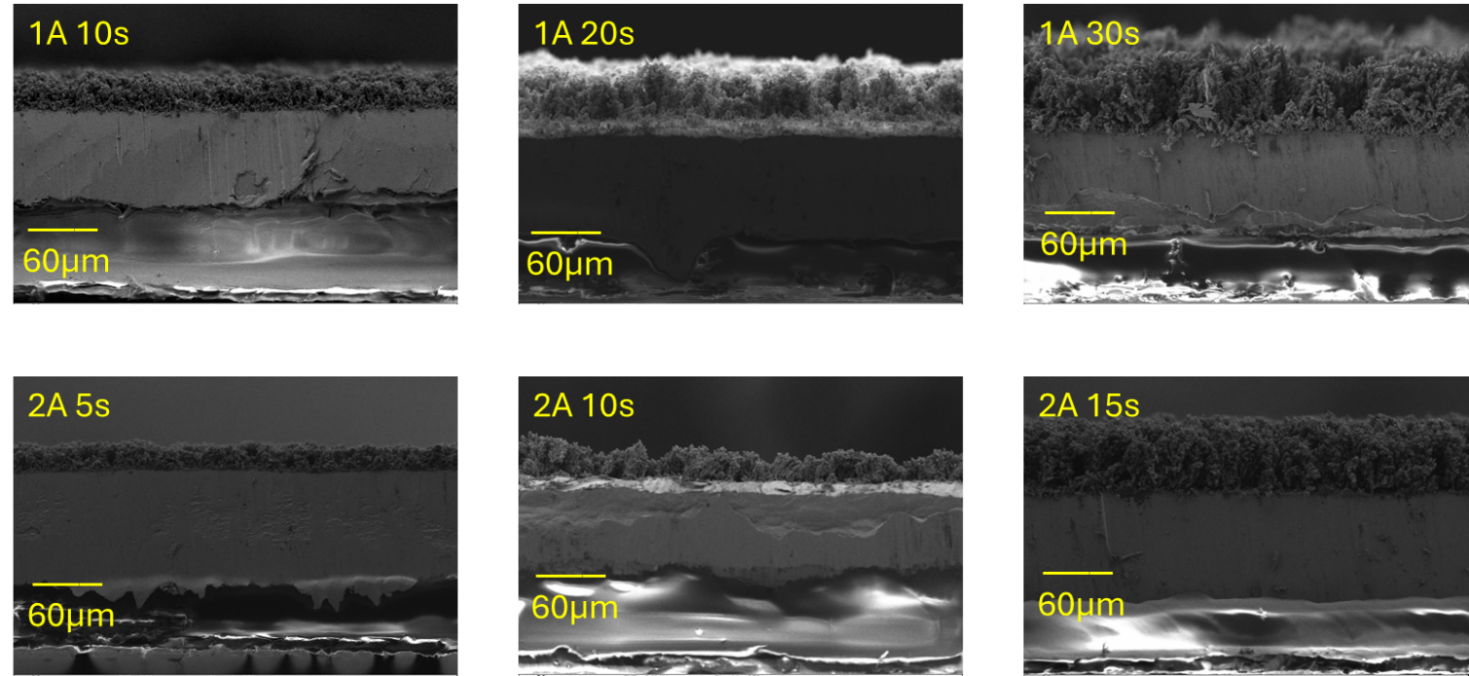


**Figure S1.** Examples of the cross-sectional SEM images of the current-time series


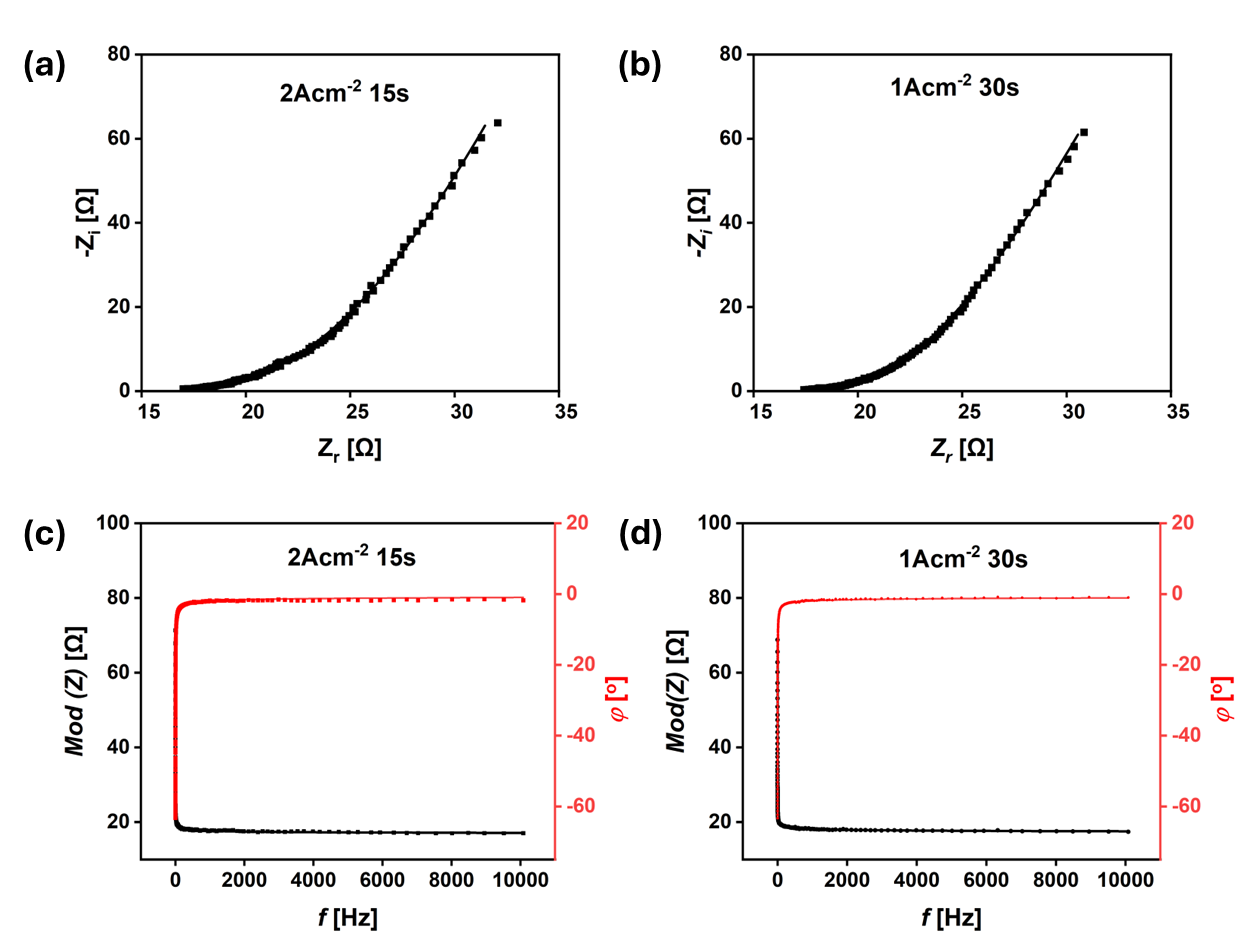


**Figure S2.** Representative fitted EIS results displayed as Nyquist plots (a and b) and Bode plots (c and d) for (2Acm^-2^ 15s) and (1Acm^-2^ 30s) samples, respectively. Experimental data are shown as scatter points, while the corresponding fitted curves are depicted as solid lines


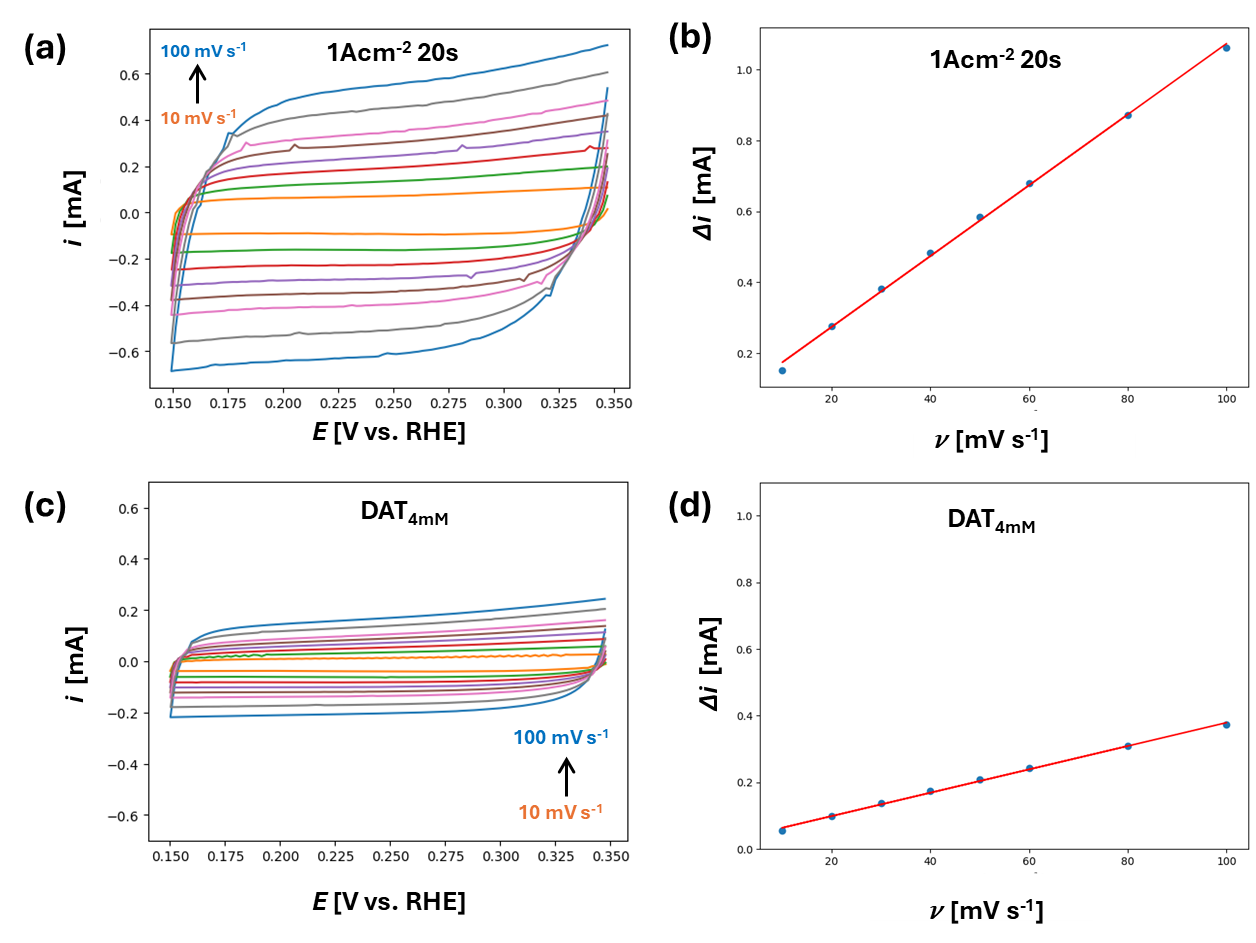


**Figure S3.** Examples of the CV curves (a and c) and the extracted plot of the summation of charging and discharging currents vs. scan rate (b and d) used for ECSA determination of the samples (1Acm^-2^ 20s) and DAT_4mM_, correspondingly


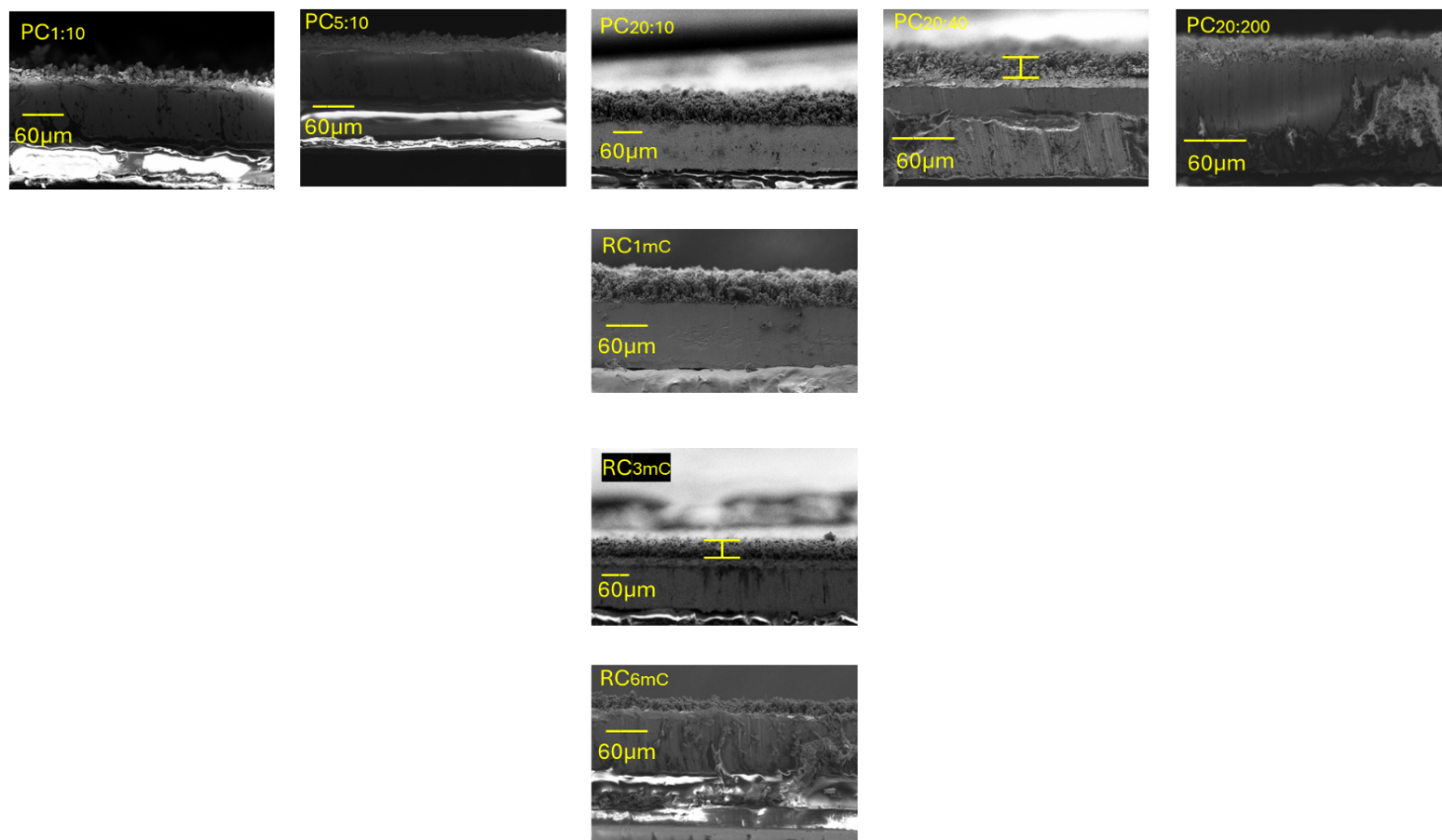


**Figure S4.**Examples of the cross-sectional SEM images of the PC and RC samples, each collected at an appropriate magnification


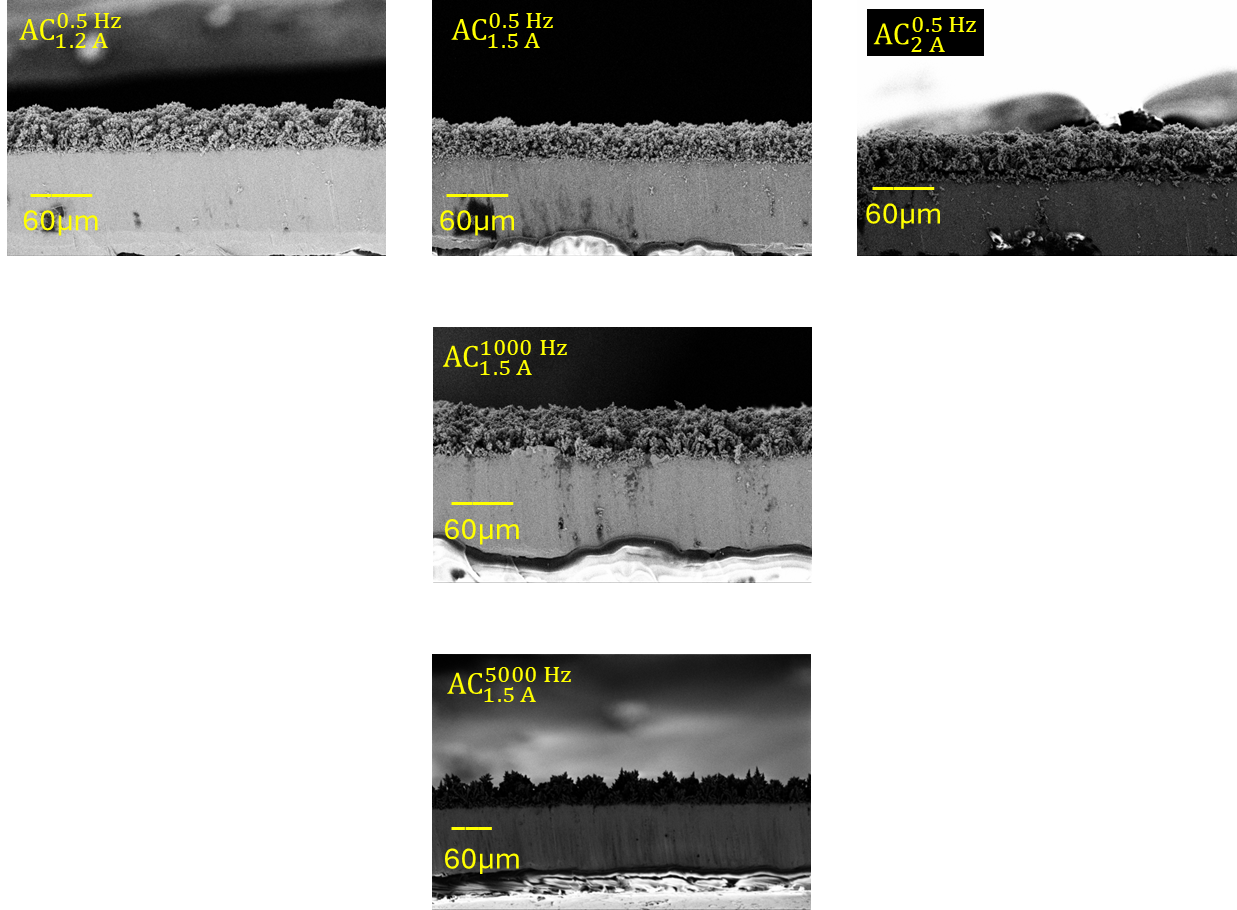


**Figure S5.** Examples of the cross-sectional SEM images of the AC samples, each collected at an appropriate magnification


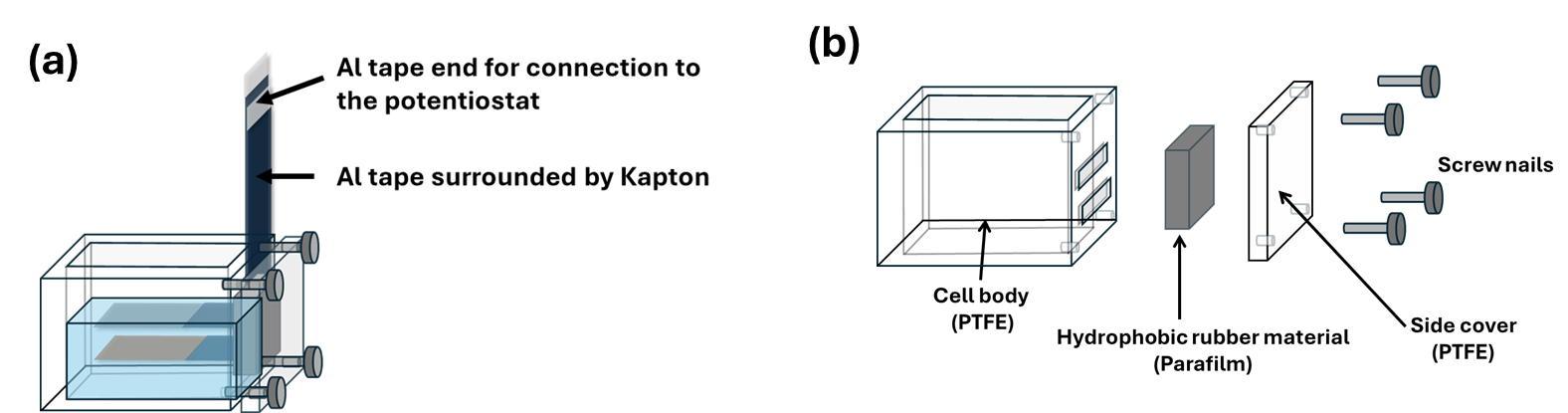


**Figure S6.**Schematic illustration of the horizontal DHBT setup (a) and the individual cell components (b)


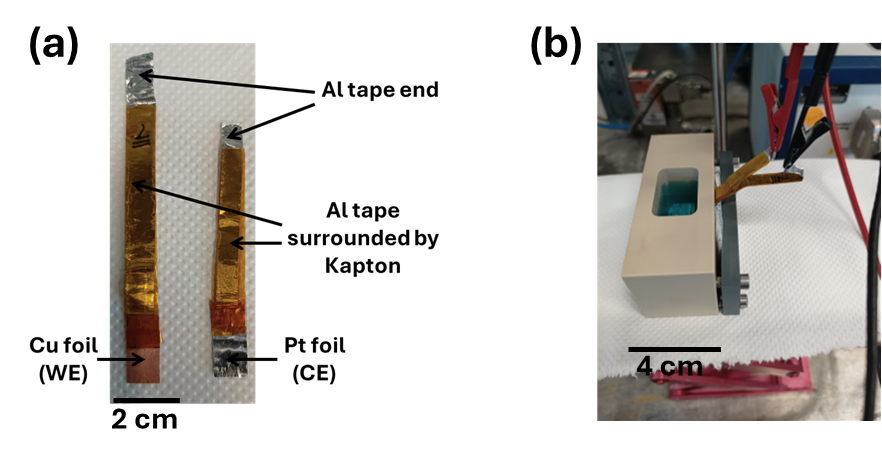


**Figure S7.** Digital photographs of the horizontal DHBT working and counter electrodes with electrical connectors (a), and the assembled cell setup (b)


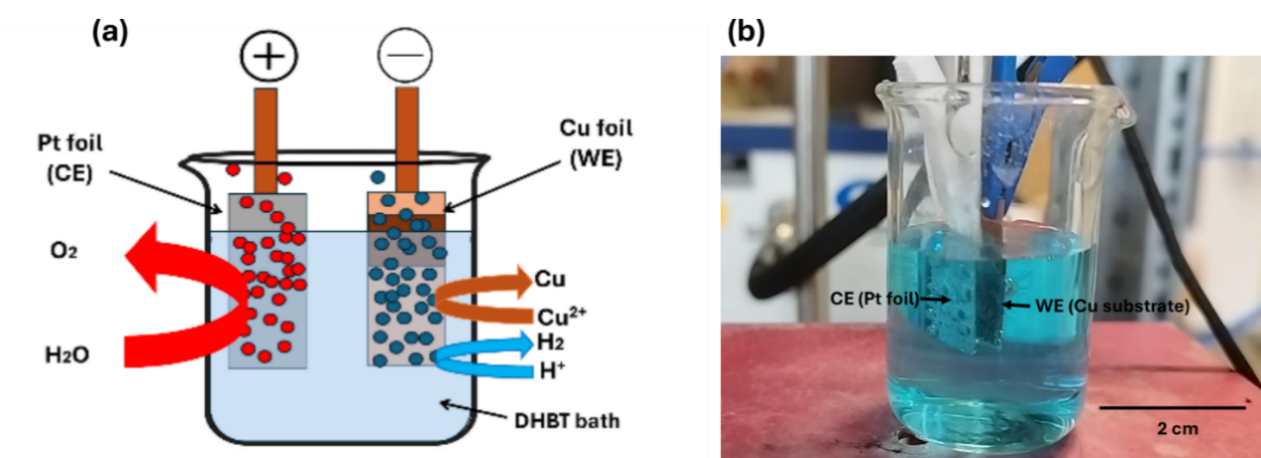


**Figure S8.** A schematic representation (a) and digital photograph (b) of the standard DHBT setup


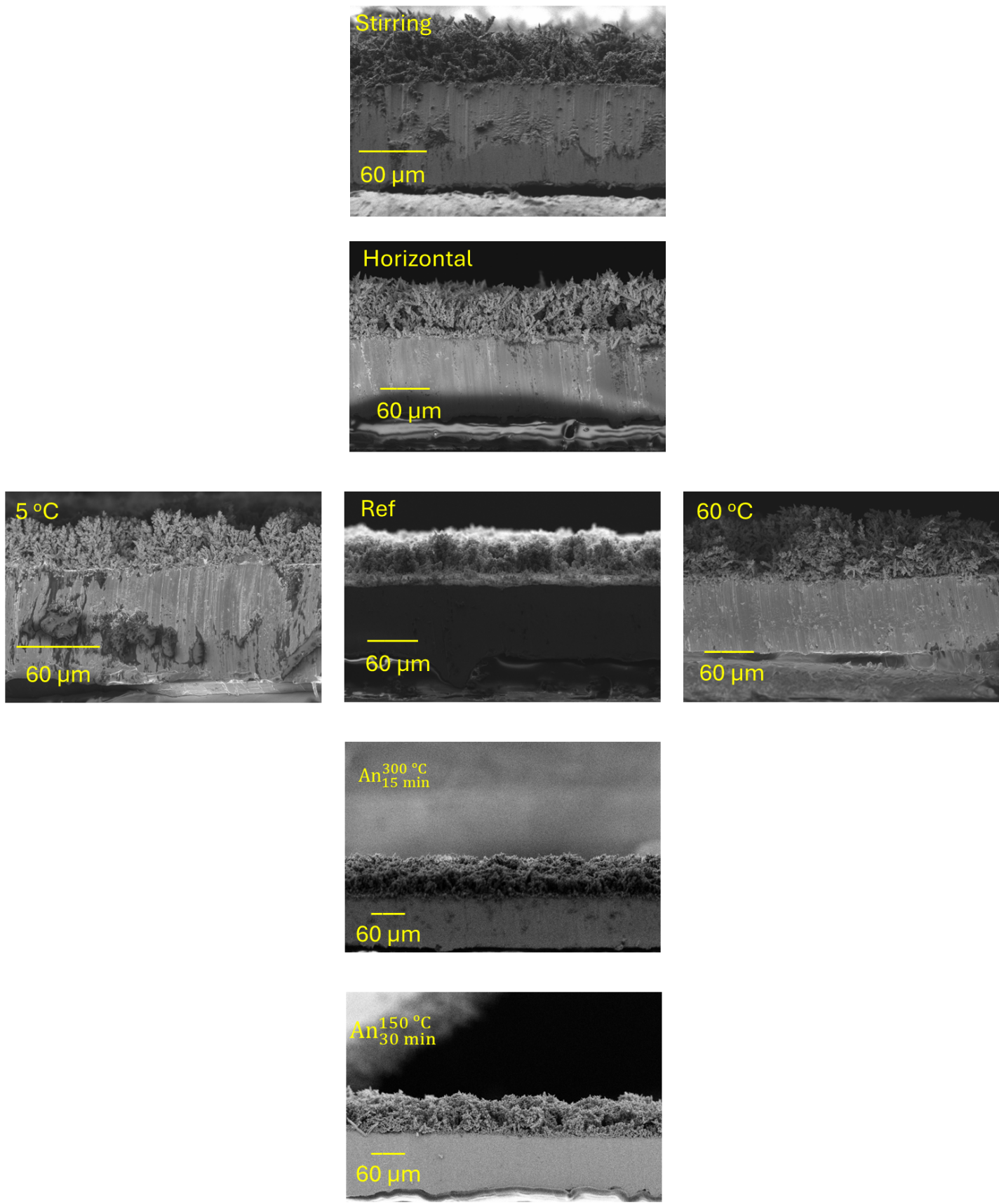


**Figure S9.**Examples of the cross-sectional SEM images of the physical effect samples, each collected at an appropriate magnification


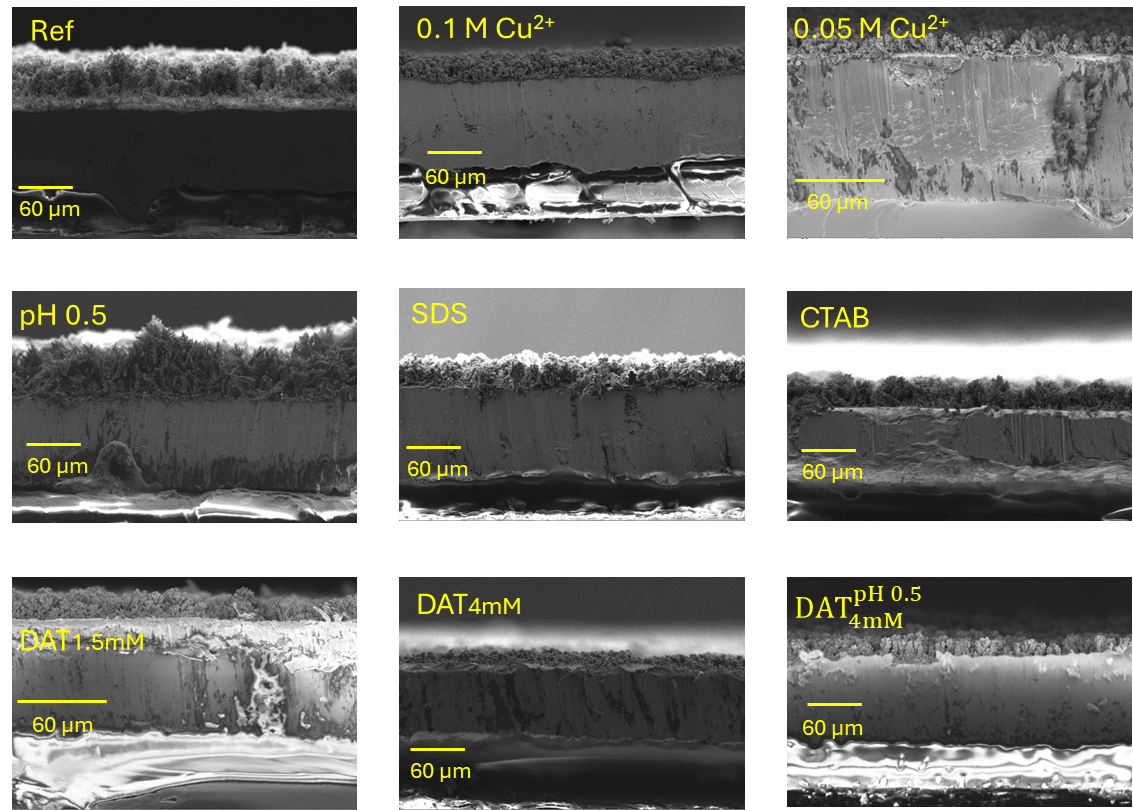


**Figure S10.** Examples of the cross-sectional SEM images of the chemical effect series samples, each collected at an appropriate magnification

**Figure S11.**Schematic representation of the DHBT process without (a) and with (b) surfactant addition to the bath. The illustration highlights the presence of surfactant molecules on the bubble surface, influencing bubble size and the resulting copper foam morphology

**Table S1.** Detailed correlation of the DHBT conditions and various properties of the electrodeposited Cu foams: deposited Cu mass, electrodeposition Faradaic efficiency (deposition FE), pore diameter, foam thickness, and the shape and size of surface nano-dendrites. An upward arrow (↑) signifies an increase in the property, a downward arrow (↓) indicates a decrease in the property, and a dash (–) represents no clear correlation between the property and the applied DHBT condition.

| DHBT parameter | Deposited mass | Deposition FE | Pore diameter | Thickness | nano-dendrite shape | nano-dendrite size |
| --- | --- | --- | --- | --- | --- | --- |
| DC + time↑ | ↑ | ↑ | ↑ | ↑ | dendrites | ↑ |
| DC + current density↑ | ↓ | ↓ | ↓ | ↓ | dendrites | ↓ |
| PC + pulse-to-pause ratio↓ | ↑ | ↑ | ↓ᵃ^)^ | ↓ | cauliflower | - |
| RC + ∆C↑ | ↓ | ↓ | ↓ | ↓ | cauliflower | - |
| AC + frequency↑ | ↑ | ↑ | ↑ | - | dendrites | ↑ |
| AC + amplitude↑ | - | - | ↑ | - | dendrites | - |
| Mechanical stirring | - | - | - | - | dendrites | ↑ |
| Horizontal alignment | ↑ | ↑ | ↑ | ↑ | dendrites | ↑ |
| Temperature↓ | ↓ | ↓ | ↓ | ↓ | dendrites | ↓ |
| Annealing | - | - | ↑ | ↑ | dendrites | ↓ |
| SDS & CTAB | ↓ | ↓ | ↓ | ↓ | dendrites | ↓ |
| DAT | - | - | ↓ | ↓ | dendrites or cauliflowersᵇ^)^ | - |
| Cu²⁺ concentration↑ | ↑ | ↑ | ↑ | ↑ | dendrites | ↑ |
| pH↑ | ↑ | ↑ | ↑ | ↑ | dendrites | ↑ |

^a)^Depends on the pulse-to-pause ratio. At very low pulse-to-pause ratios, no honeycomb-like structures were obtained; ^b)^Depends on DAT concentration.

**
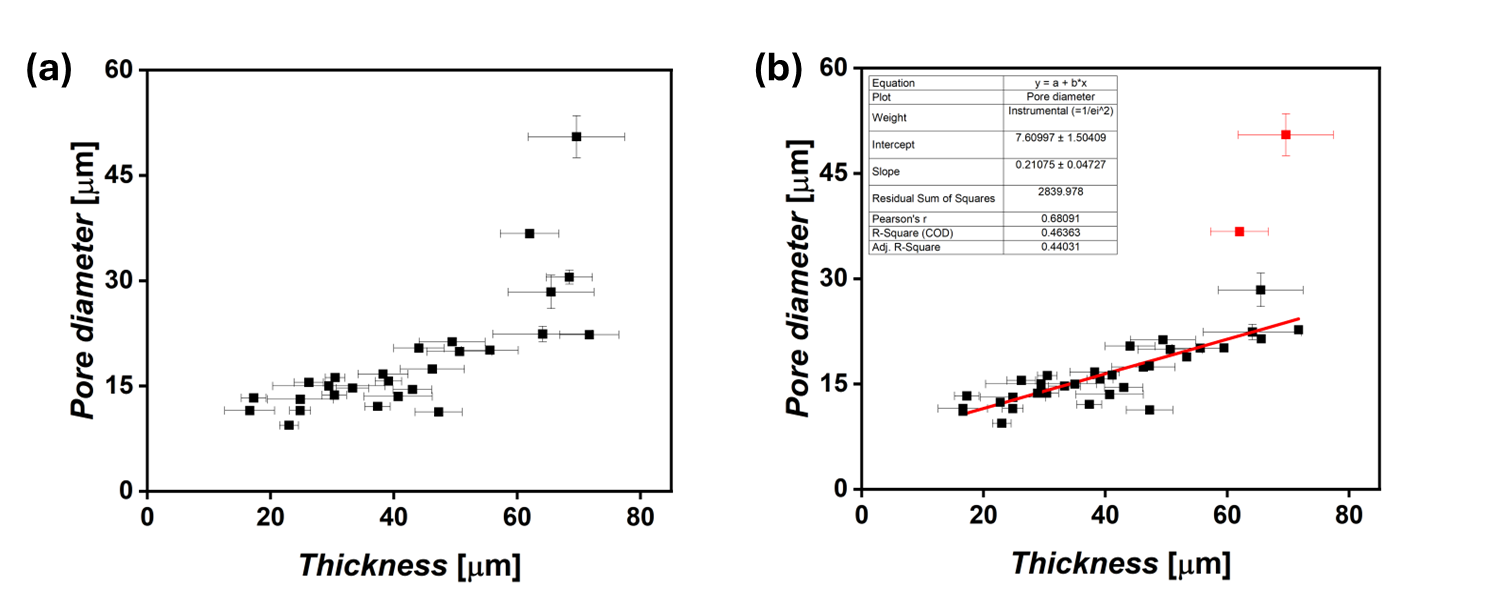
**

**Figure S12.** Relationship between foam thickness and pore diameter for the Cu foam samples investigated in this study (a), and the corresponding linear fit (b), with outliers shown in red


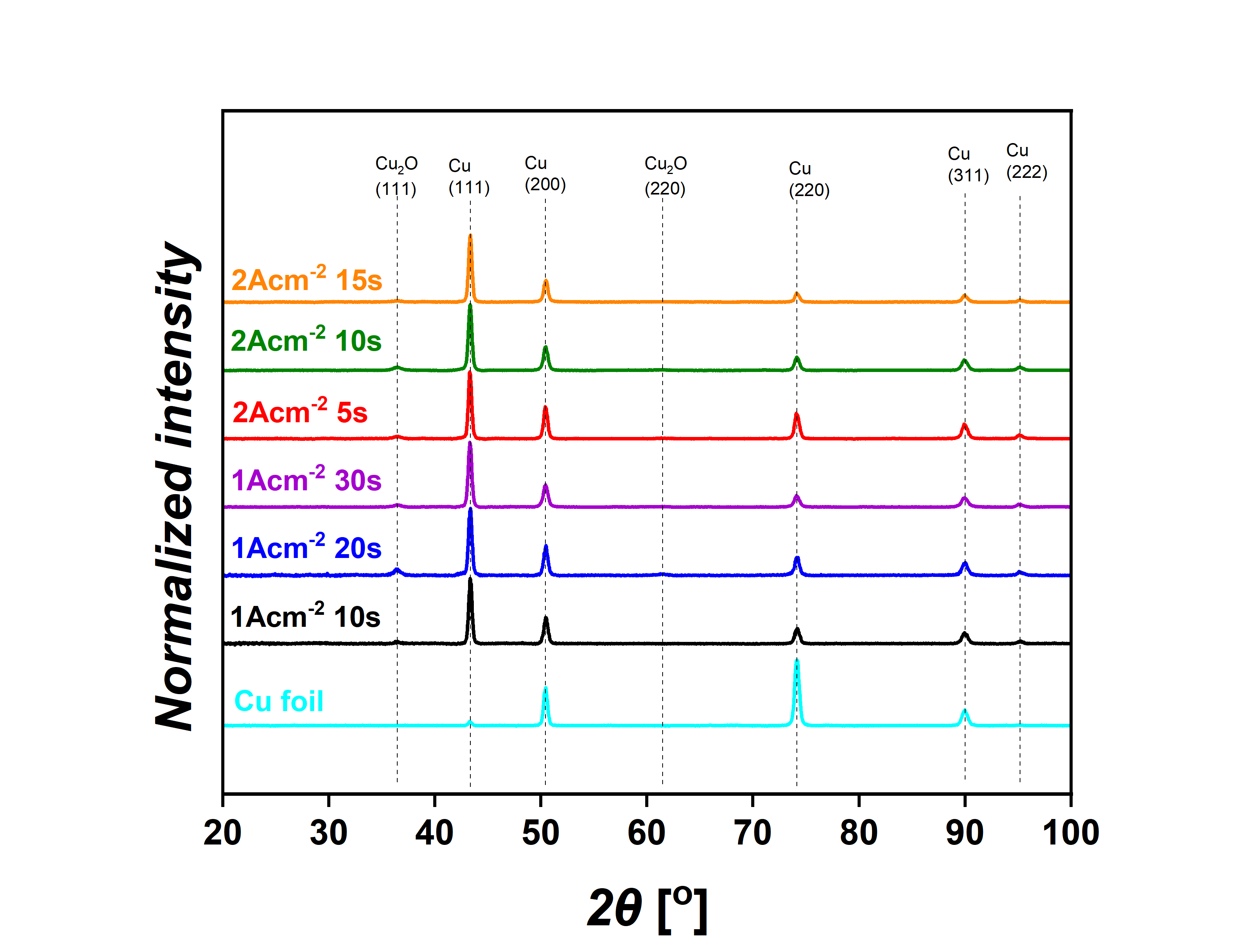


**Figure S13.** XRD patterns of the DHBT current-time series and the Cu foil used as a substrate for DHBT


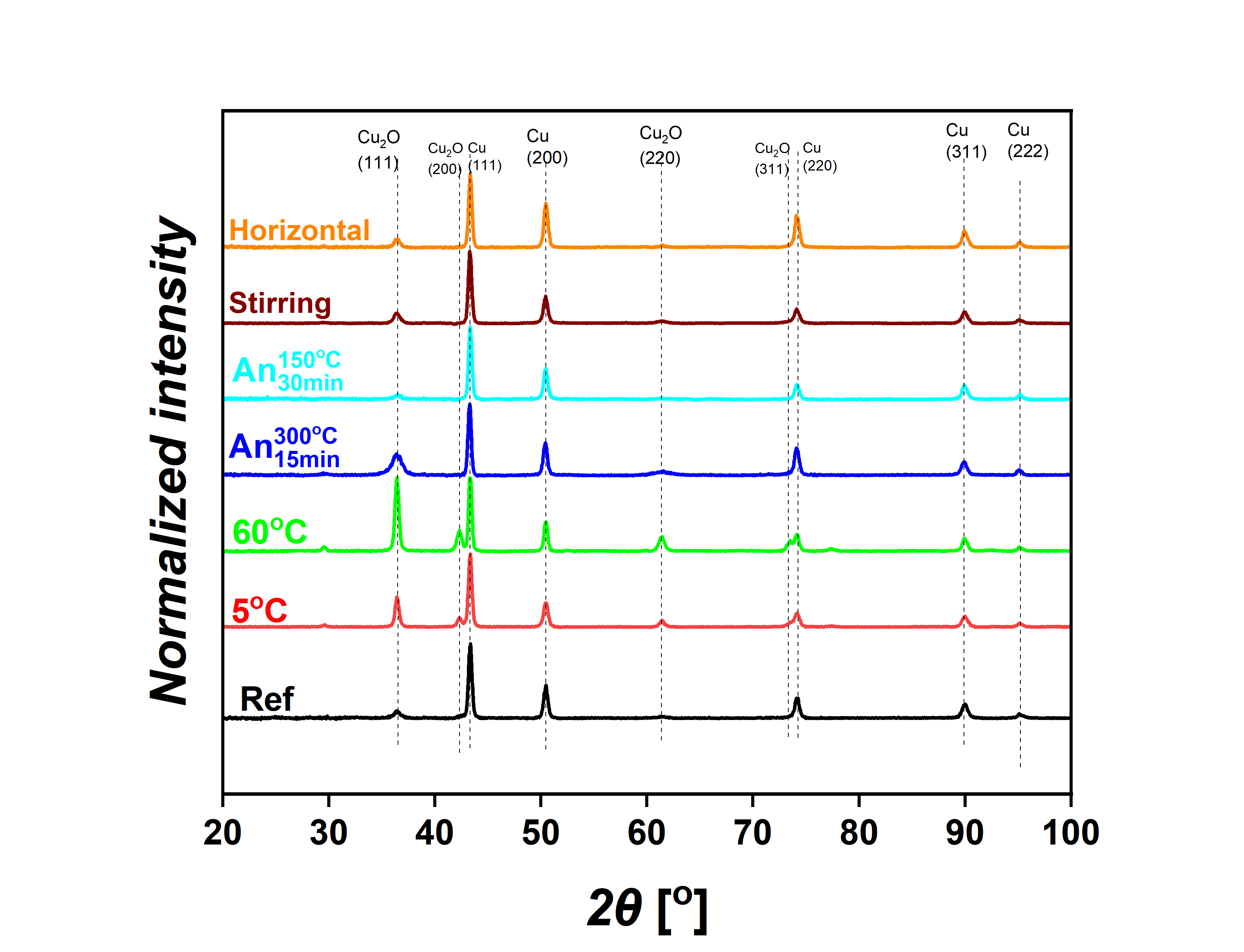


**Figure S14.** XRD patterns of the physical DHBT effect series


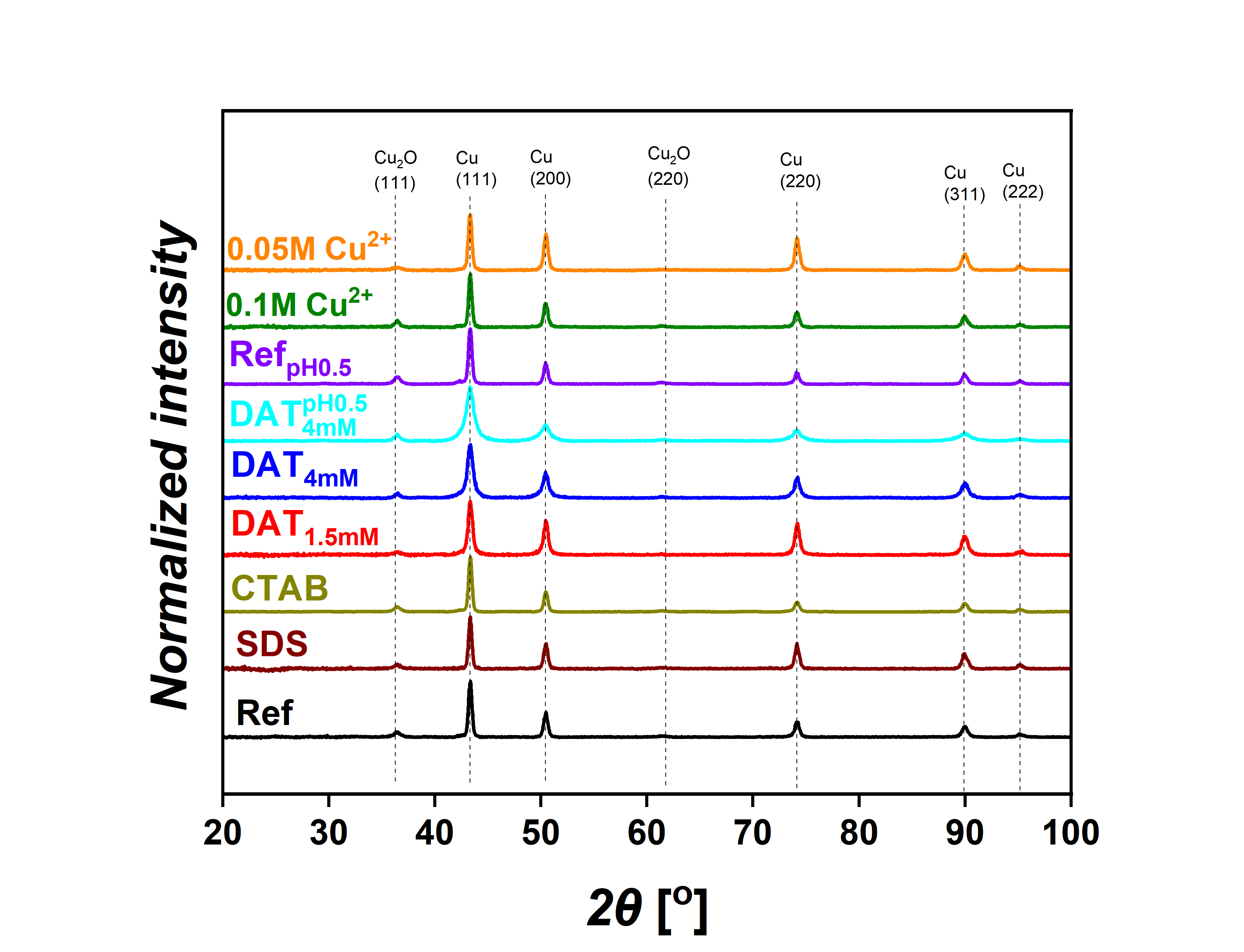


**Figure S15.** XRD patterns of the chemical DHBT effect series

**Table S2.** Summarized XRD results of the Cu foam samples synthesized in this study: the crystallite size, micro-strain, and dominant Cu phase

| Sample | | Crystallite size  [nm] | | Micro-strain  [x 10⁻³] | | Dominant Cu phase | |
| --- | --- | --- | --- | --- | --- | --- | --- |
| 1A 10s | | 82 | | 1.4 | | Cu | |
| 1A 20s | | 92 | | 1.7 | | Cu | |
| 1A 30s | | 41 | | 1.1 | | Cu | |
| 2A 5s | | 139 | | 1.7 | | Cu | |
| 2A 10s | | 50 | | 1.2 | | Cu | |
| 2A 15s | | 46 | | 0.7 | | Cu | |
| PC₁:₁₀ | | 107 | | 1.4 | | Cu | |
| PC₅:₁₀ | | 63 | | 1.4 | | Cu | |
| PC₂₀:₁₀ | | 36 | | 0.6 | | Cu | |
| PC₂₀:₄₀ | | 50 | | 1.4 | | Cu | |
| PC₂₀:₂₀₀ | | 116 | | 1.6 | | Cu | |
| RC₁ₘc | | 29 | | 0.5 | | Cu | |
| RC₃ₘc | | 37 | | 1 | | Cu | |
| RC₆ₘc | | 60 | | 1.7 | | Cu | |
| $\mathrm{AC}_{1.2A}^{0.5Hz}$ | | 58 | | 1.4 | | Cu | |
| $\mathrm{AC}_{1.5A}^{0.5Hz}$ | | 69 | | 1.2 | | Cu | |
| $\mathrm{AC}_{2A}^{0.5Hz}$ | | 87 | | 1.6 | | Cu | |
| $\mathrm{AC}_{1.5A}^{1000Hz}$ | | 58 | | 1.8 | | Cu | |
| $\mathrm{AC}_{1.5A}^{5000Hz}$ | | 50 | | 0.6 | | Cu | |
| 5°C | | 41 | | 0.9 | | Cu | |
| 60°C | | 99 | | 1.1 | | Cu | |
| stirring | | 50 | | 1.3 | | Cu | |
| Horizontal | | 53 | | 0.9 | | Cu | |
| $\mathrm{An}_{30min}^{150℃}$ | | 36 | | 0.3 | | Cu | |
| $\mathrm{An}_{15min}^{300℃}$ | | 66 | | 1.0 | | Cu | |
| SDS | | 66 | | 1.2 | | Cu | |
| CTAB | | 46 | | 0.7 | | Cu | |
| DAT₁.₅_mM_ | | 20 | | 0 | | Cu | |
| DAT_4mM_ | | 11 | | -1.5 | | Cu | |
| $\mathrm{DAT}_{4mM}^{pH0.5H}$ | | 7 | | -1.2 | | Cu | |
| 0.1 M Cu²⁺ | | 50 | | 1.0 | | Cu | |
| 0.05 M Cu²⁺ | | 48 | | 1.2 | | Cu | |
| pH 0.5 | | 53 | | 0.9 | | Cu | |


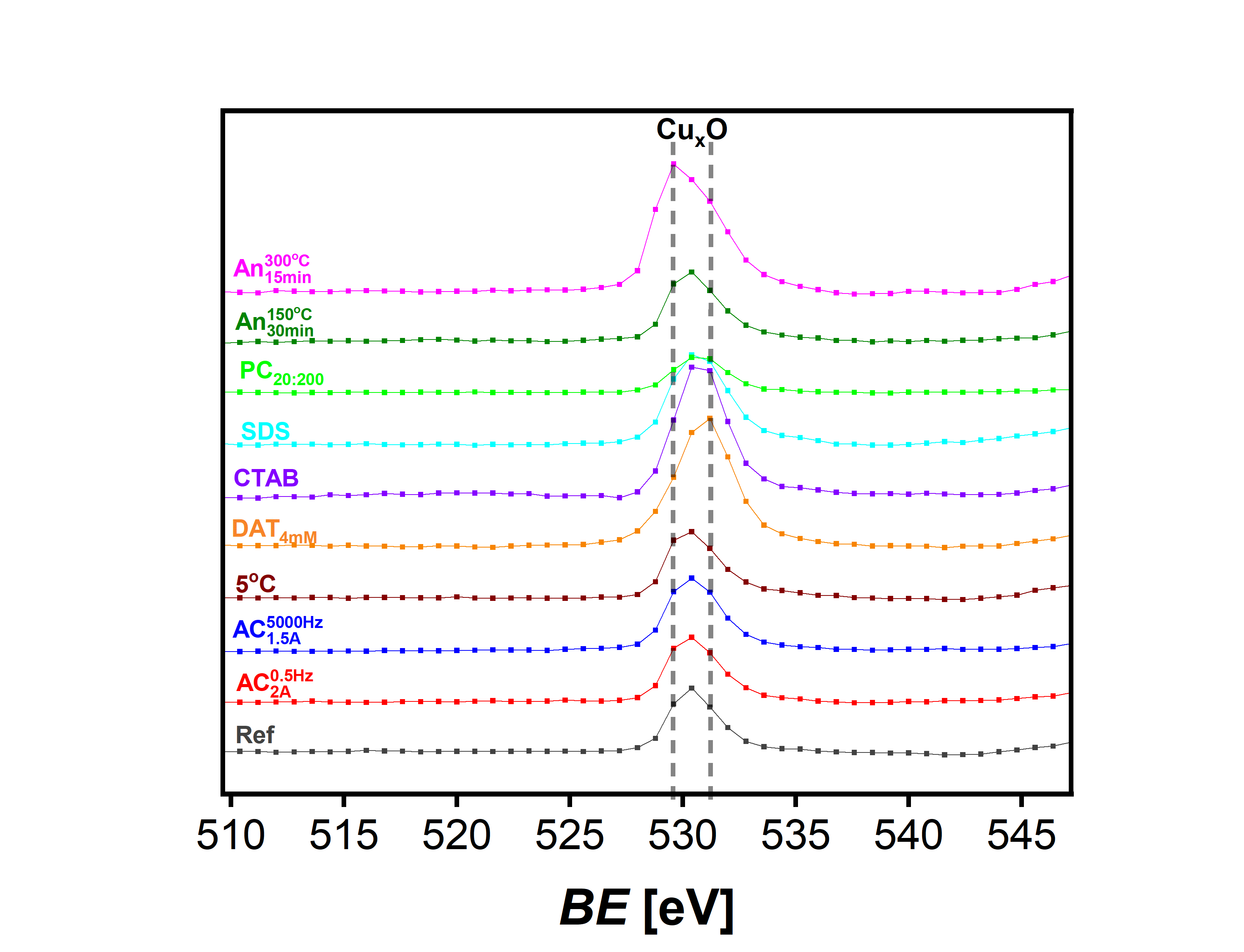


**Figure S16.** XPS spectra for O 1s region of Cu foam samples


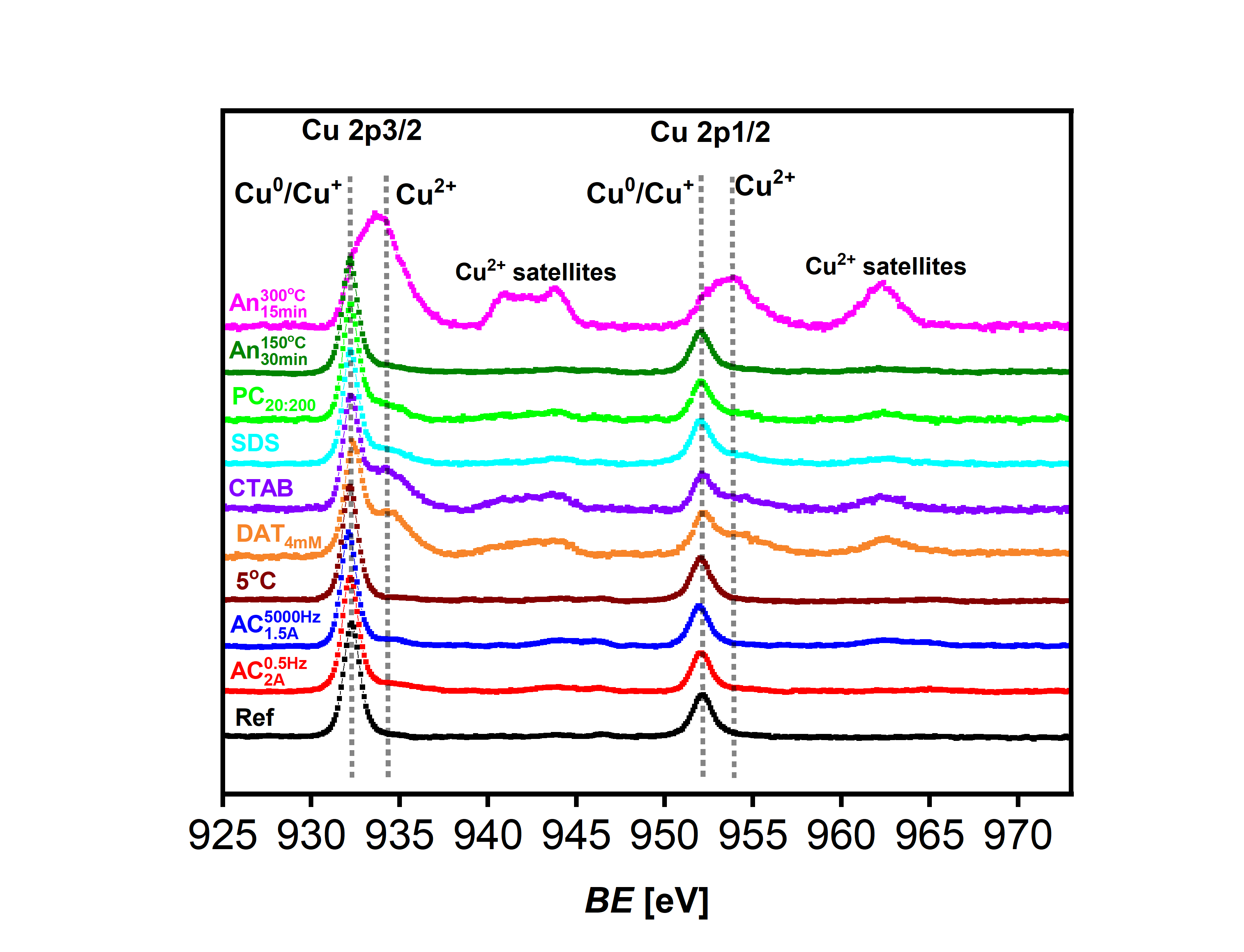


**Figure S17.** XPS spectra for Cu 2p region of Cu foam samples


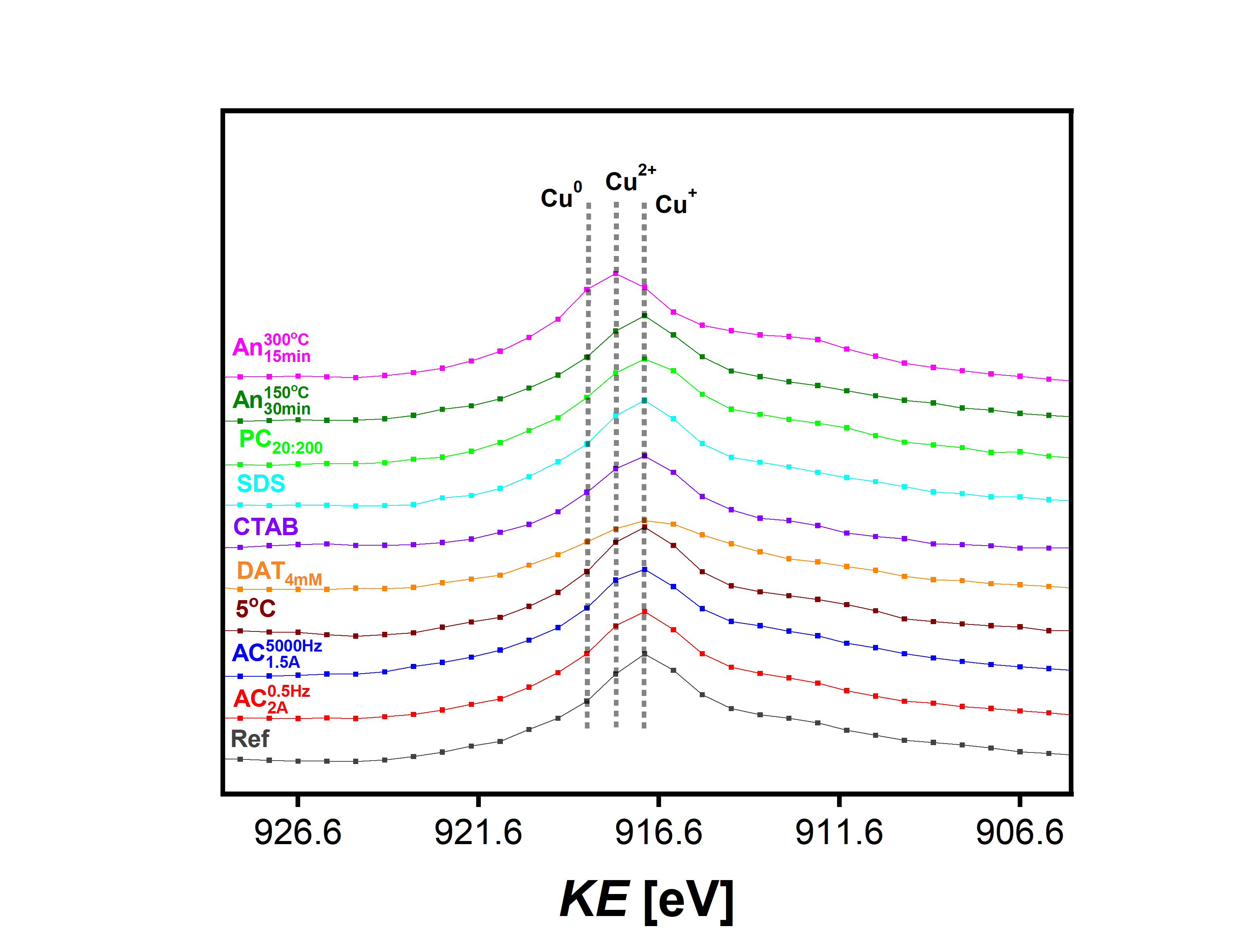


**Figure S18.** XPS spectra for Cu LMM Auger region of Cu foam samples


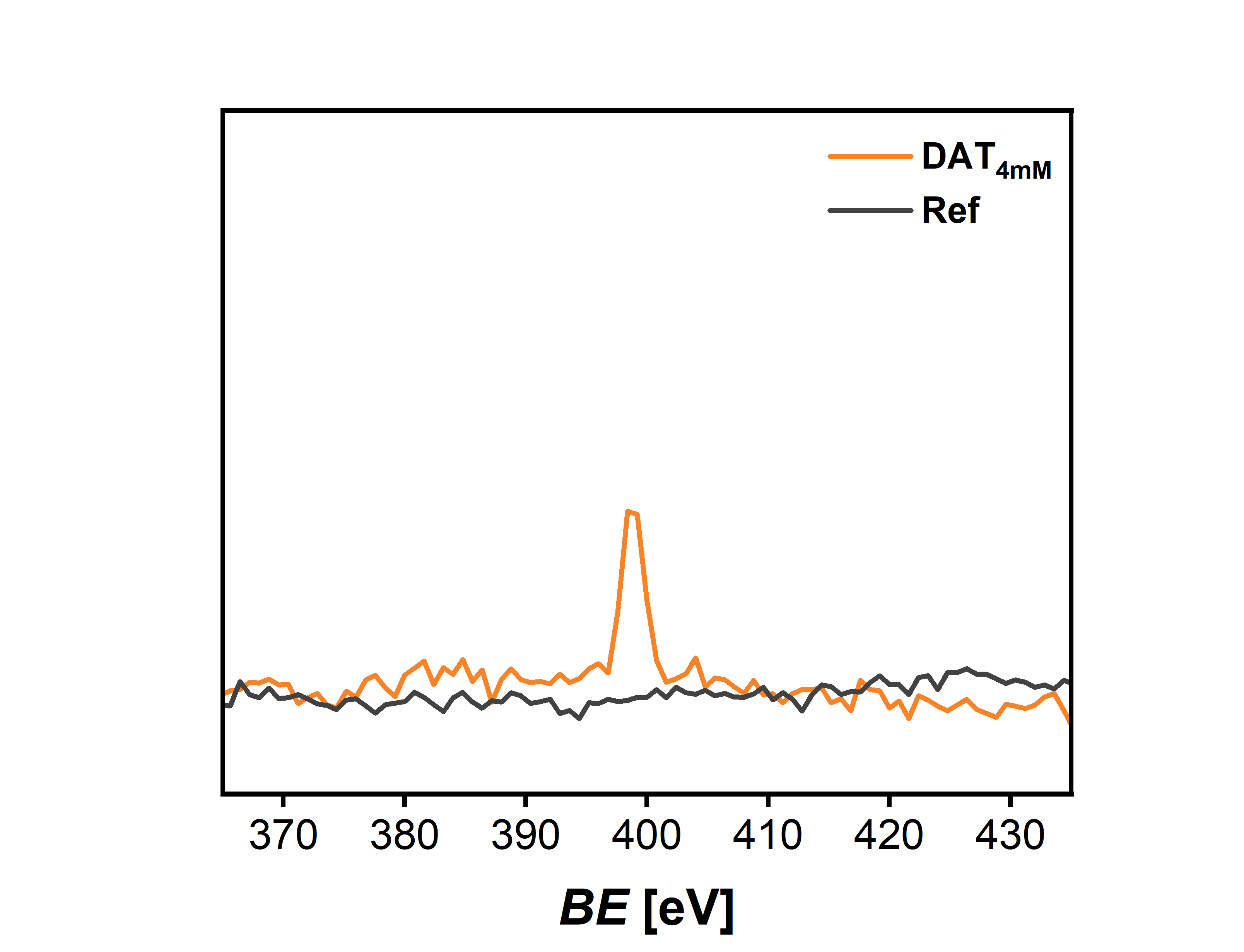


**Figure S19.** XPS spectra for N 1s region of DAT_4mM_ and Ref samples


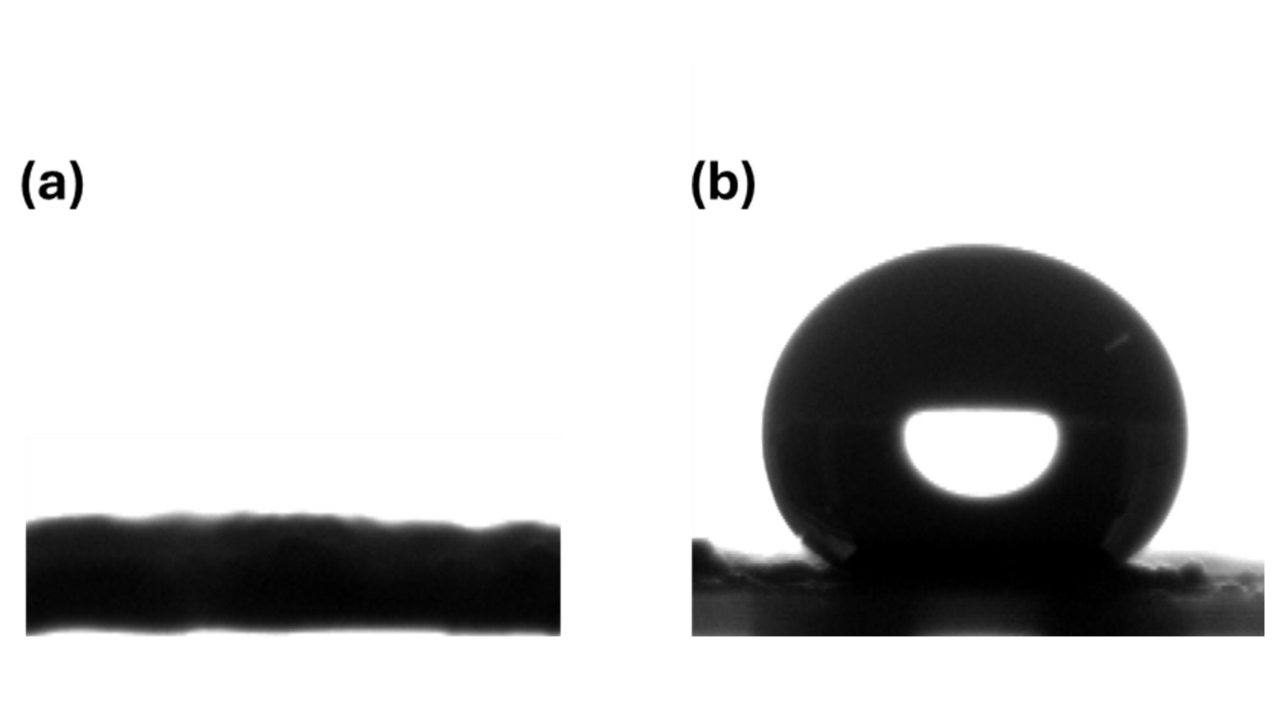


**Figure S20.** Contact angle images of the DC_30C cm_^-2^ sample before (a) and after (b) ionomer infiltration

**
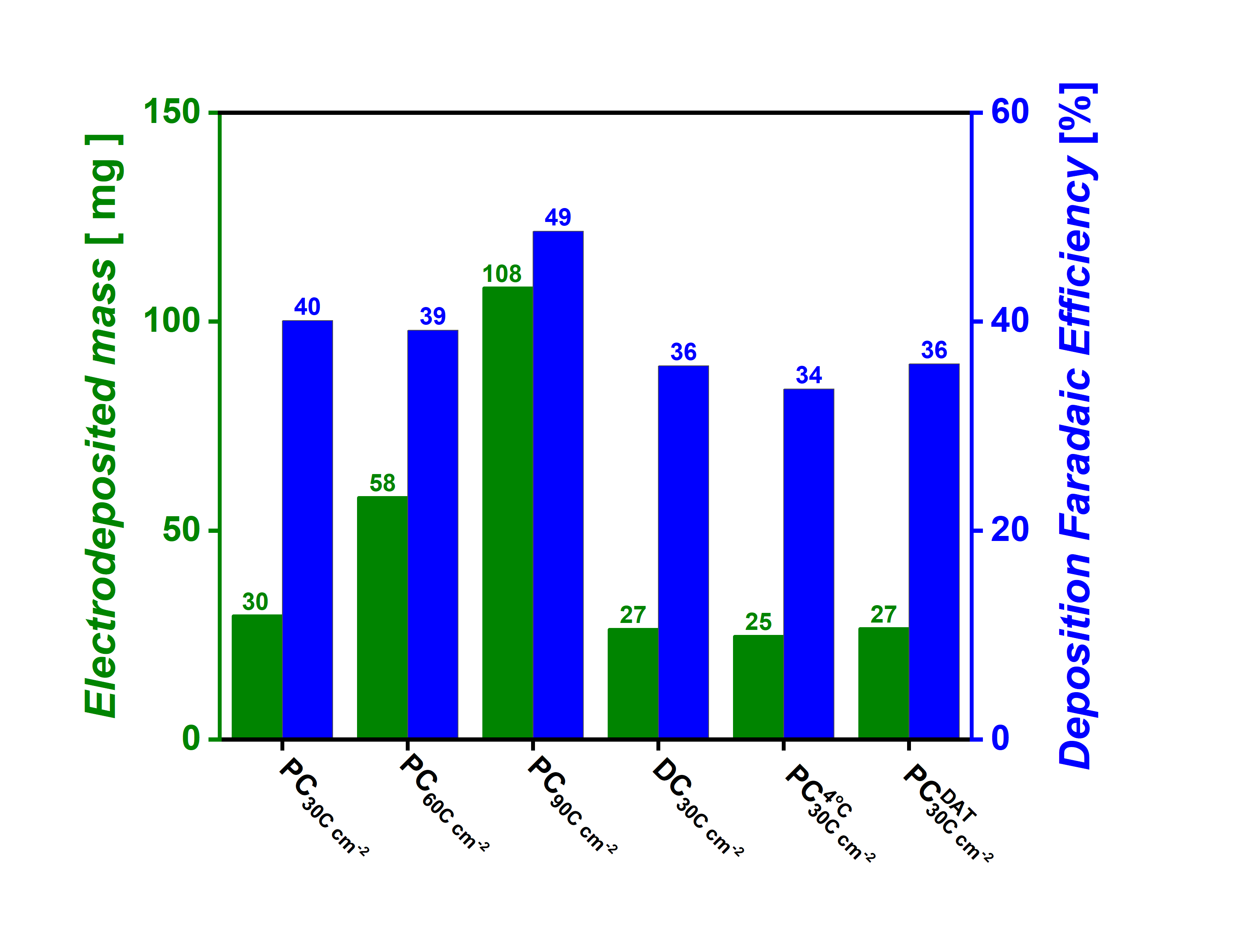
**

**Figure S21.** Gravimetric analysis of the Cu foam GDEs, showing the electrodeposited mass (green axis) and corresponding deposition Faradaic efficiencies (blue axis)

**
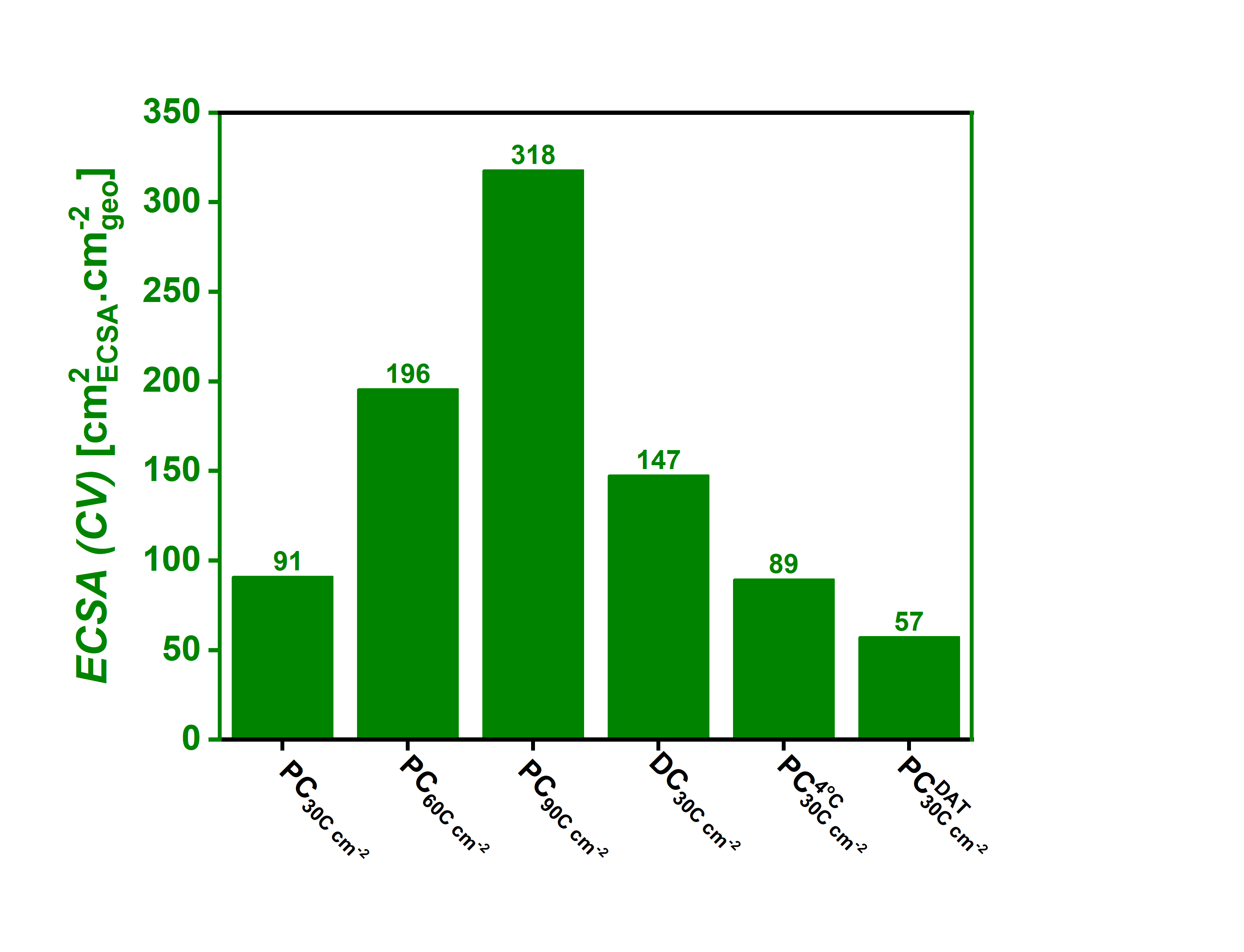
**

**Figure S22.** CV-based ECSA values of Cu foam GDE samples

^
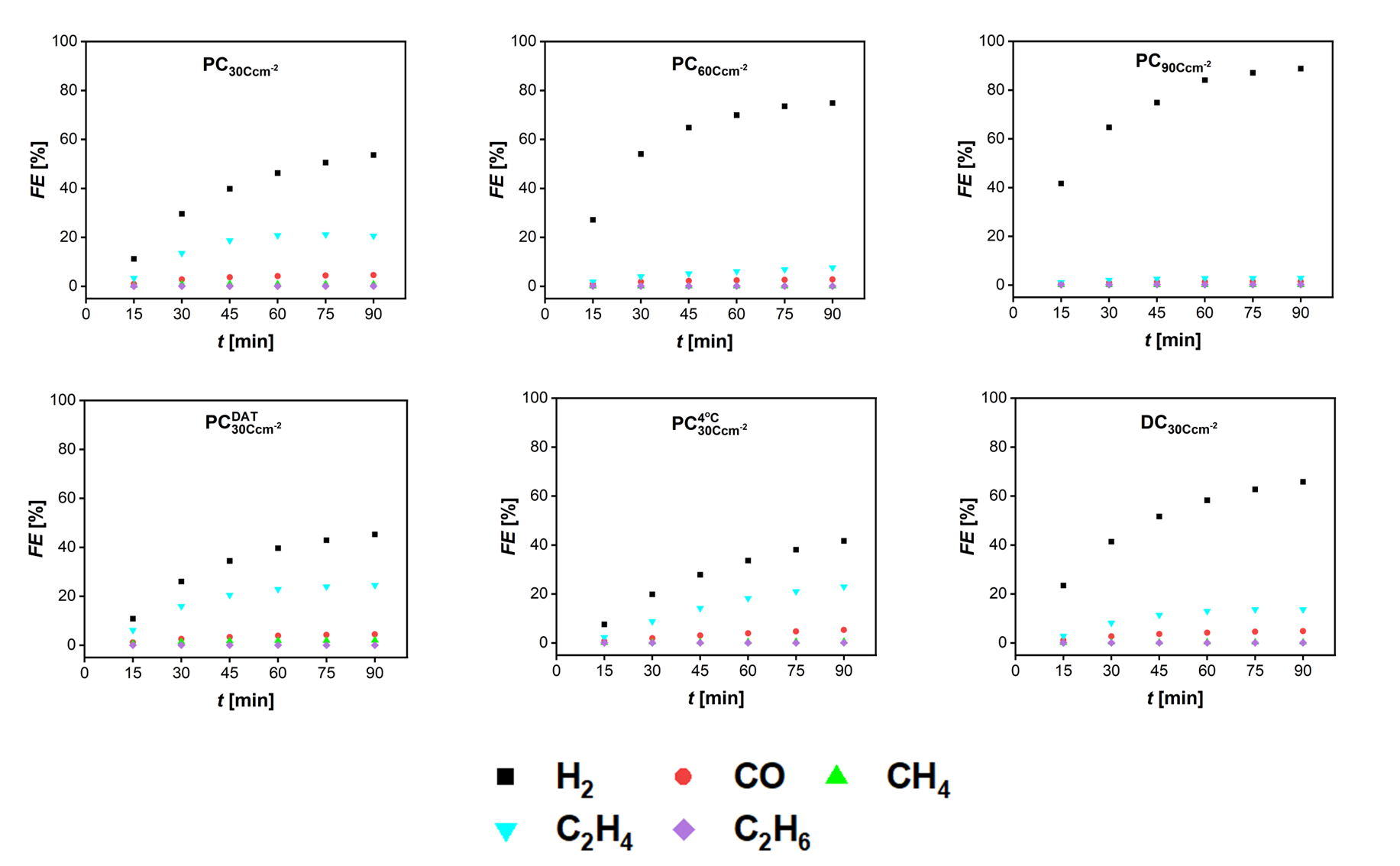
^

**Figure S23.** Faradic efficiencies of CO_2_RR gaseous products for the Cu foam GDEs measured every 15 minutes during chronopotentiometric runs at 200 mA cm^-2^ for 1.5 hours

^
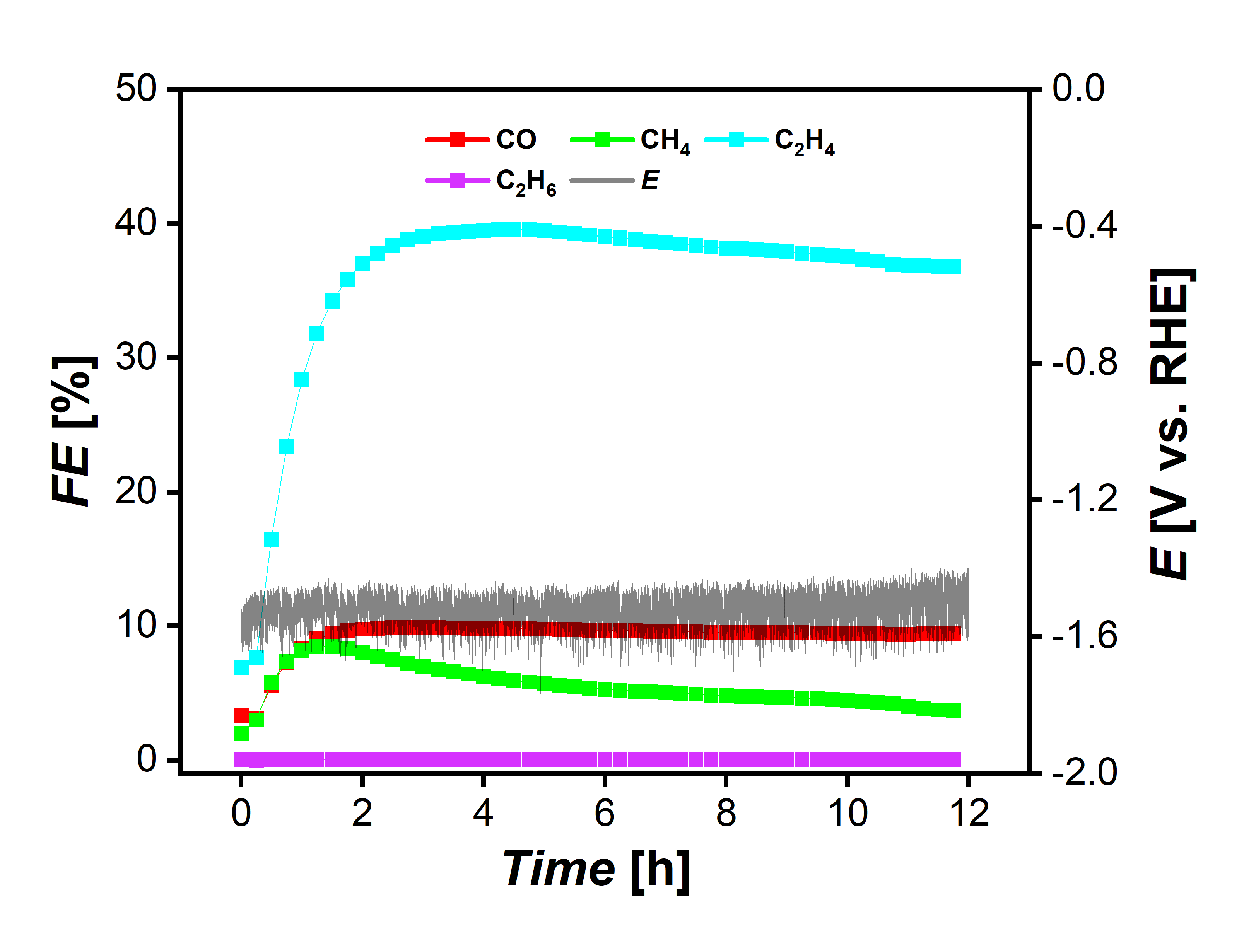
^

**Figure S24.** Faradic efficiencies of CO_2_RR gaseous products of the DAT-modified GDE and the measured potential, recorded for 12 h at an applied current density of 200 mA cm^-2^

*
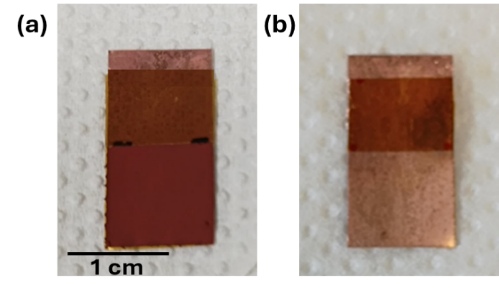
*

**Figure S25.** Digital photographs of a Cu foam sample after DHBT (a) and the Cu substrate before DHBT (b)


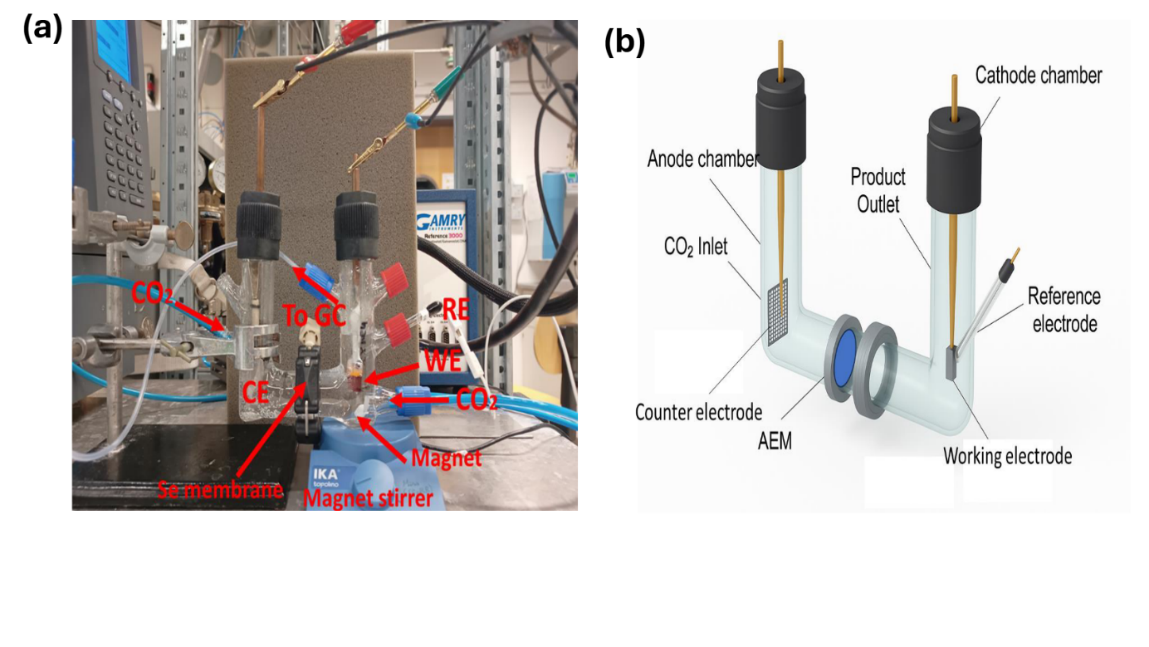


**Figure S26.** A digital photograph (a) and schematic representation (b) of the experimental setup of the H-cell used for the ECSA measurements of model Cu foams of 1 cm^2^ geometric area


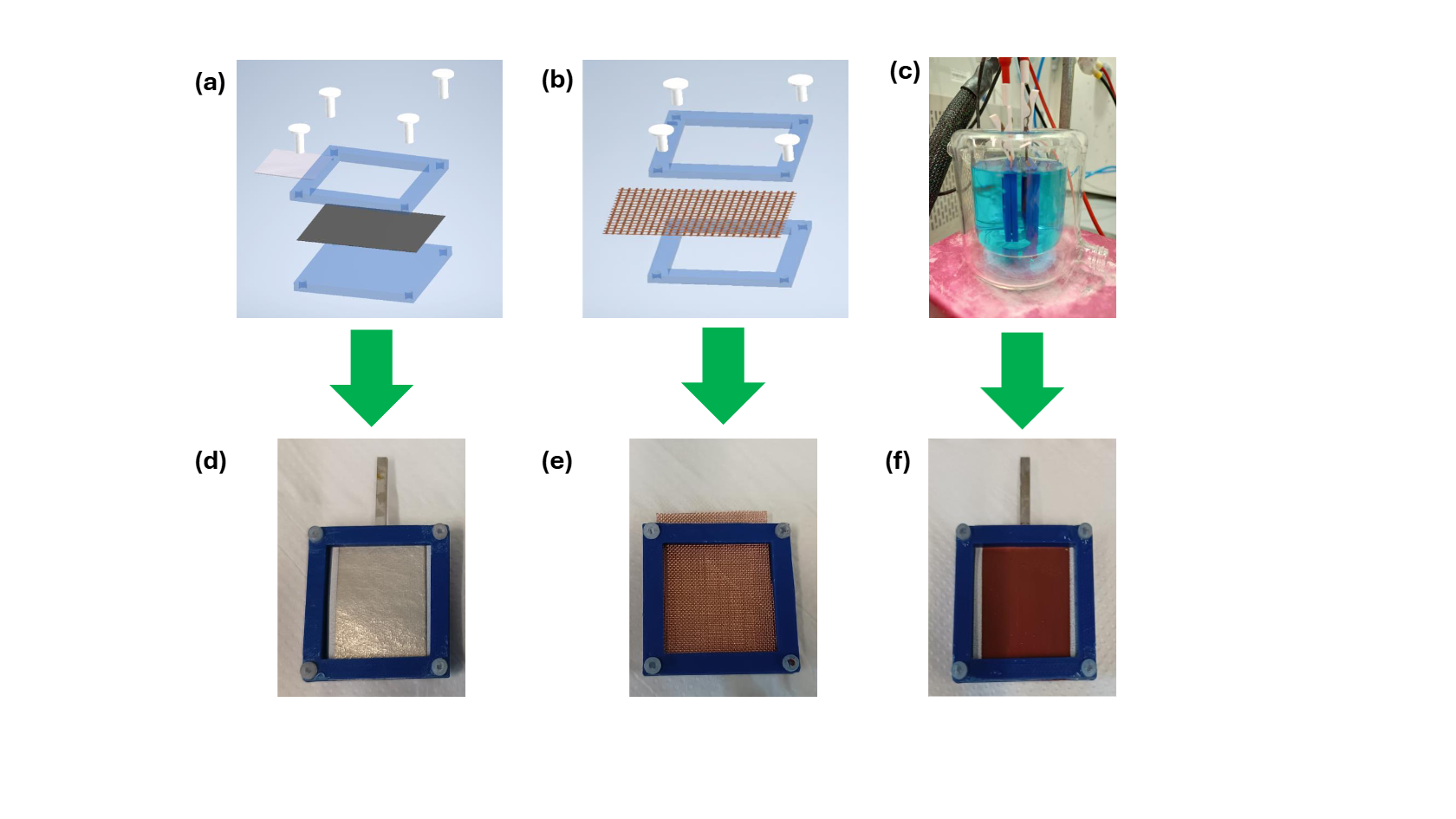


**Figure S27.** Schematic representations of the 3D-printed WE (a) and CE (b) holders with the respective digital photos of the full assembled electrodes (d and e), a digital photo of the DHBT configuration (c), and a digital photo of a Cu-foam GDE (f)


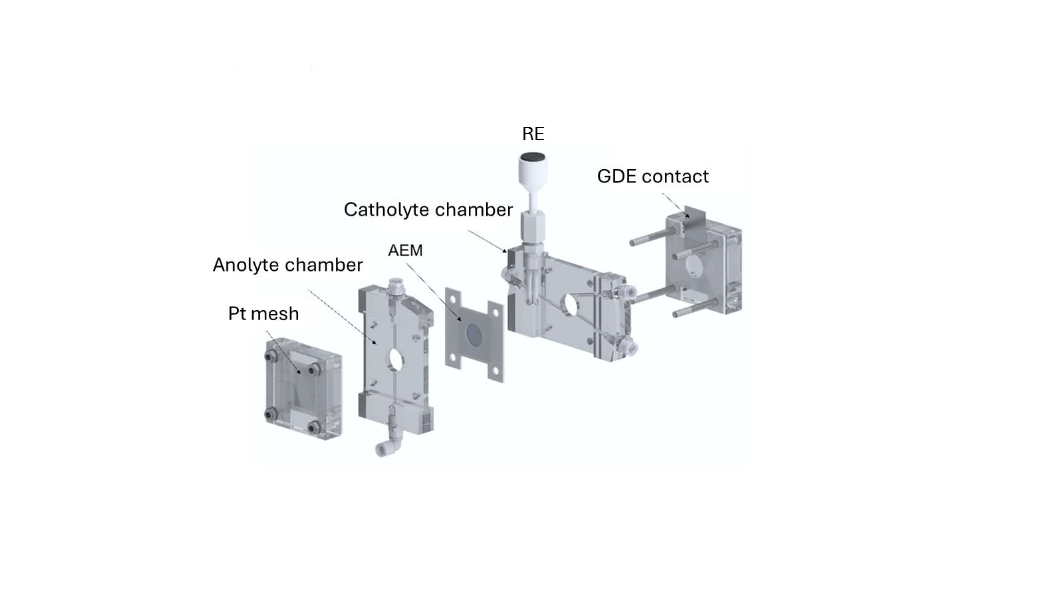


**Figure S28.** A schematic representation of the flow cell used for CO_2_ electrolysis on Cu foam GDEs. Reproduced under terms of the CC-BY 3.0 license from ref.^[1]^ 2024, H. Hoffmann, M. Kutter, and J. Osiewacz, et al., published by Royal Society of Chemistry


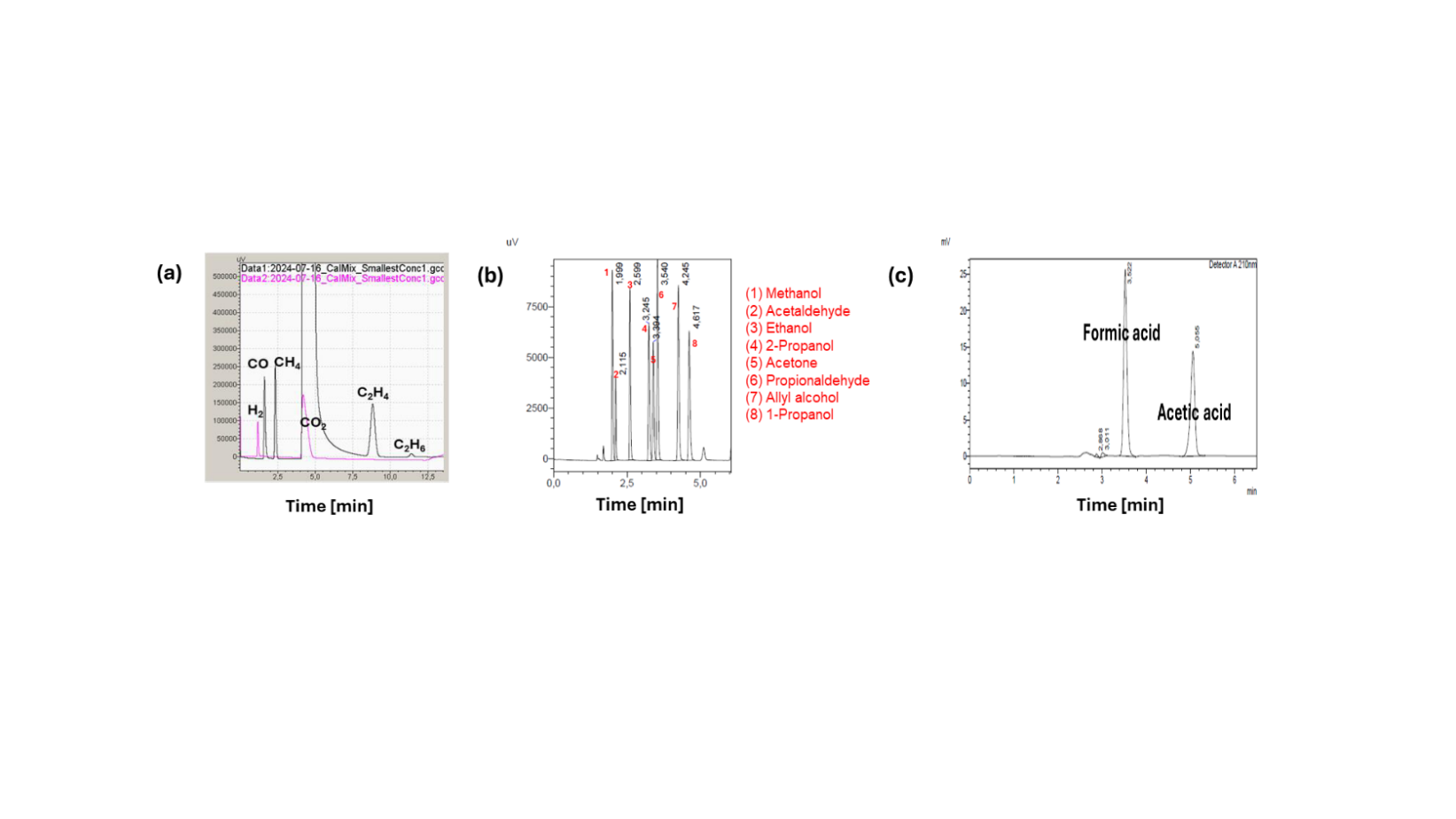


**Figure S29.** Chromatograms from online GC (a), offline GC (b), and HPLC (c), each illustrating the CO_2_RR products for which the respective system is calibrated


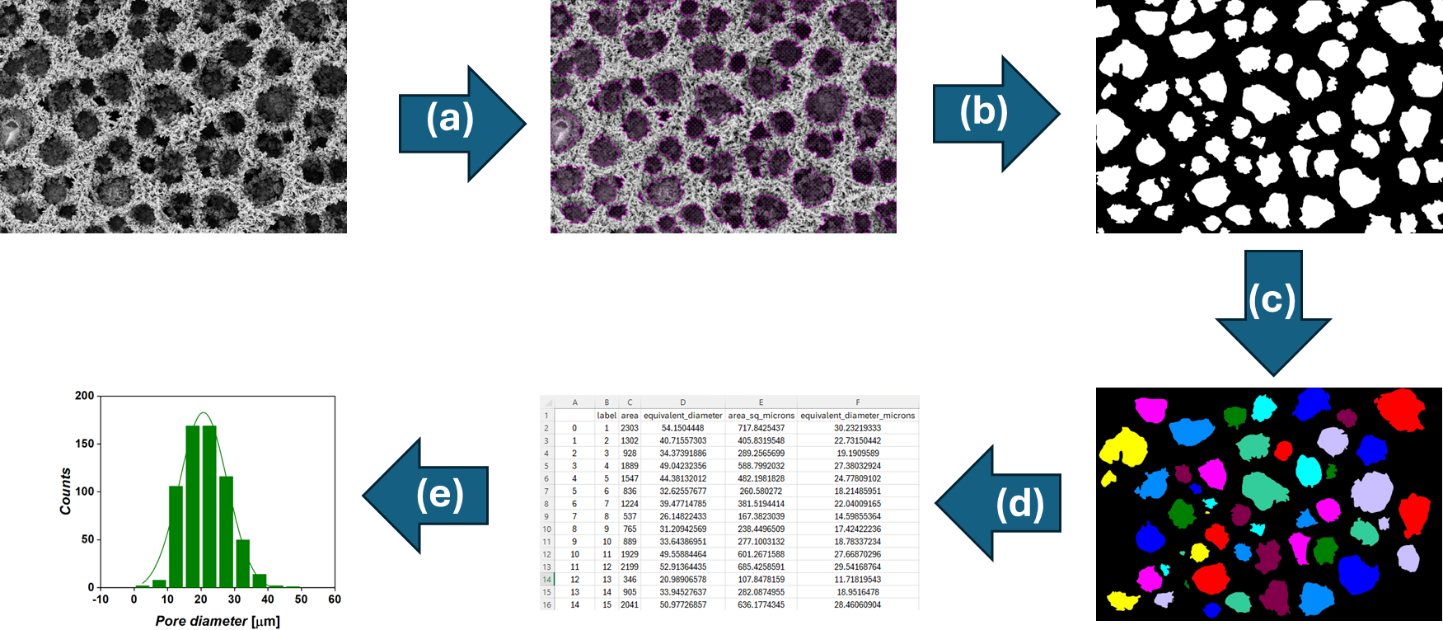


**Figure S30.** Workflow for determining the pore diameter distribution: (a) annotation of the original images; (b) extraction of annotated pixels; (c) exclusion of edge-touching pores; (d) calculation of pore area and diameter; and (e) generation of the final pore diameter distribution

**References**

[1] H. Hoffmann, M. Kutter, and J. Osiewacz, et al., “Highly selective Ag foam gas diffusion electrodes for CO_2_ electroreduction by pulsed hydrogen bubble templation,” EES Catalysis, vol. 2, no. 1, pp. 286–299, 2024.
